# Discovery, Molecular Mechanisms, and Industrial Applications of Cold-Active Enzymes

**DOI:** 10.3389/fmicb.2016.01408

**Published:** 2016-09-09

**Authors:** Margarita Santiago, César A. Ramírez-Sarmiento, Ricardo A. Zamora, Loreto P. Parra

**Affiliations:** ^1^Department of Chemical Engineering and Biotechnology, Centre for Biochemical Engineering and Biotechnology, Universidad de ChileSantiago, Chile; ^2^Schools of Engineering, Medicine and Biological Sciences, Institute for Biological and Medical Engineering, Pontificia Universidad Católica de ChileSantiago, Chile; ^3^Departamento de Biología, Facultad de Ciencias, Universidad de ChileSantiago, Chile; ^4^Department of Chemical and Bioprocesses Engineering, School of Engineering, Pontificia Universidad Católica de ChileSantiago, Chile

**Keywords:** cold-active enzymes, psychrophiles, biocatalysis, extremophiles, protein structure and function, protein engineering, biotechnological applications

## Abstract

Cold-active enzymes constitute an attractive resource for biotechnological applications. Their high catalytic activity at temperatures below 25°C makes them excellent biocatalysts that eliminate the need of heating processes hampering the quality, sustainability, and cost-effectiveness of industrial production. Here we provide a review of the isolation and characterization of novel cold-active enzymes from microorganisms inhabiting different environments, including a revision of the latest techniques that have been used for accomplishing these paramount tasks. We address the progress made in the overexpression and purification of cold-adapted enzymes, the evolutionary and molecular basis of their high activity at low temperatures and the experimental and computational techniques used for their identification, along with protein engineering endeavors based on these observations to improve some of the properties of cold-adapted enzymes to better suit specific applications. We finally focus on examples of the evaluation of their potential use as biocatalysts under conditions that reproduce the challenges imposed by the use of solvents and additives in industrial processes and of the successful use of cold-adapted enzymes in biotechnological and industrial applications.

## Introduction

In the context of global needs for sustainability and clean manufacturing technologies, biocatalysts are an attractive alternative for the achievement of chemical transformations (Wohlgemuth, 2010; Bornscheuer et al., 2012). Enzymes are non-toxic, biodegradable, and efficient/selective biocatalysts with outstanding catalytic properties, offering high levels of safety, low energy consumption, and an overall environmentally friendly production procedure (Saha and Demirjian, 2001; Dunn, 2012; Wang M. et al., 2012). A high interest has been displayed in enzymes from organisms living on extreme ecosystems, because they work under harsh environments, which are conditions mostly found in industrial processes. Among extremophiles, microorganisms living in cold environments have become a very interesting source for the identification and isolation of novel cold-active enzymes (Russell, 2000; D'Amico et al., 2002a; Feller, 2003, 2010). The use of enzymes that remain active at low temperatures has a great potential for industrial biocatalysis in terms of energy savings by lowering the required temperature of a reaction without sacrificing enzyme activity. Cold-active enzymes can also prevent undesirable chemical reactions occurring at higher temperatures, while simultaneously offering an amenable procedure for their rapid heat-inactivation due to their structural thermolability, which is of special interest in food industry for eliminating the use of chemical-based inactivation (Russell, 1998; Gerday et al., 2000; Georlette et al., 2004; Margesin and Feller, 2010). Although most cold-active enzymes have been isolated from psychrophiles and psychrotolerant microorganisms, some enzymes displaying high activity at low temperatures have also been obtained from mesophilic and even from thermophilic organisms.

## Microorganisms have colonized cold places on earth

Despite the harsh conditions that cold environments present for human life, microorganisms have colonized cold places on Earth. Depending on their optimal growth temperature, these microorganisms can be psychrophilic or psychrotolerants. Psychrophilic organisms are able to grow at low temperatures, between −20 and 10°C, and unable to grow at temperatures higher that 15°C. Unlike psychrophiles, psychrotolerant organisms grow optimally at 20–25°C but also have a high metabolic activity and growth capacity at temperatures below 0°C (Pikuta et al., 2007). Typically, psychrotolerant microorganisms are found in terrestrial cold environments and psychrophiles in marine ecosystems. Microorganisms living on these cold places are mainly bacteria, yeasts, fungi and algae, and this biodiversity has been extensively reviewed (Cowan et al., 2007; Yumoto, 2013).

Constantly cold environments (< 5°C) cover ~80% of the Earth's biosphere and include mainly the Polar Regions, deep water and marine sediments of the oceans, and glaciers of high mountains (Pikuta et al., 2007; Huston, 2008). Polar regions account for 15% of the Earth's surface and include the Antarctic and the Arctic Circle with their polar ice sheets, glaciers, and permafrost (Cowan et al., 2007; Pikuta et al., 2007). To have an idea about the temperatures of this region, an example is the Antarctic air, which has annual temperatures below 0°C and during winter the temperature can reach −80°C (Cowan et al., 2007). Permafrost represents more than 20% of terrestrial soils (Deming, 2002) and it contains a large number of viable microorganisms which have retained their life over geological times (Rivkina et al., 2004). Organisms living in permafrost are mostly psychrotolerant and not psychrophiles (Morita, 1975). Deep water and marine sediments of the oceans cover 75% of the Earth's surface. They have an average temperature of 3°C, complete absence of light, high pressures and low nutrient availability, however numerous microorganisms have been identified and isolated from these ecosystems, the majority of them psychrophiles (Cowan et al., 2007). Glaciers, on other continents than the Polar Regions, cover an area of 15,861,766 km^2^. Here, microorganisms live in the liquid veins at ice grain inter-junctions and in the thin liquid film on the surfaces of mineral grains, which contain substrates for their survival (Miteva, 2008).

## Cold-active enzyme discovery

The most routine approach for discovering novel enzymes is the culture of microorganisms that express a protein of interest. This culture-dependent methodology has been successful for the isolation and characterization of many biocatalysts (Yang and Ding, 2014). Culture-independent techniques have emerge to increase the rate of enzyme discovery, since the microorganisms that can be cultured under laboratory conditions represent only a minor fraction (1–5%) of the microbial diversity, and therefore of their enzymes (Ekkers et al., 2012). For microorganisms from extreme environments a second problem arises when cultivation is attempted, as the harsh conditions that extremophiles need to grow increases the difficulty of obtaining enough biomass to have good DNA yields for cloning effectiveness (Ferrer et al., 2007). Some techniques to address this obstacle and improve the cultivation of cold-adapted microorganisms have recently been reviewed (Vester et al., 2015). Metagenomics is the main culture-independent approach and involves DNA extraction of an environmental sample followed by the construction of metagenome libraries for the isolation of target genes (Temperton and Giovannoni, 2012). Another approach, where no environmental sample is needed, is to use the vast information available in genome databases, which provides the possibility to identify novel enzymes by computational genomics (Gong et al., 2013). Considering that the access to extreme environments like constantly cold regions is not easy, genome mining emerges as a huge opportunity for the discovery of novel cold-adapted enzymes. However, to date it has not been used as the preferred alternative, maybe because only a few genomes of psychrophiles have been deposited in public databases.

Cold-active enzymes isolated by metagenomic approaches have been recently reviewed (Cavicchioli et al., 2011; Vester et al., 2015). Therefore, here we focus on cold-active enzymes derived from cultivated microorganisms and in some cases from synthetic genes.

### Natural hosts and diversity of cold-active enzymes

**Figure 1:**
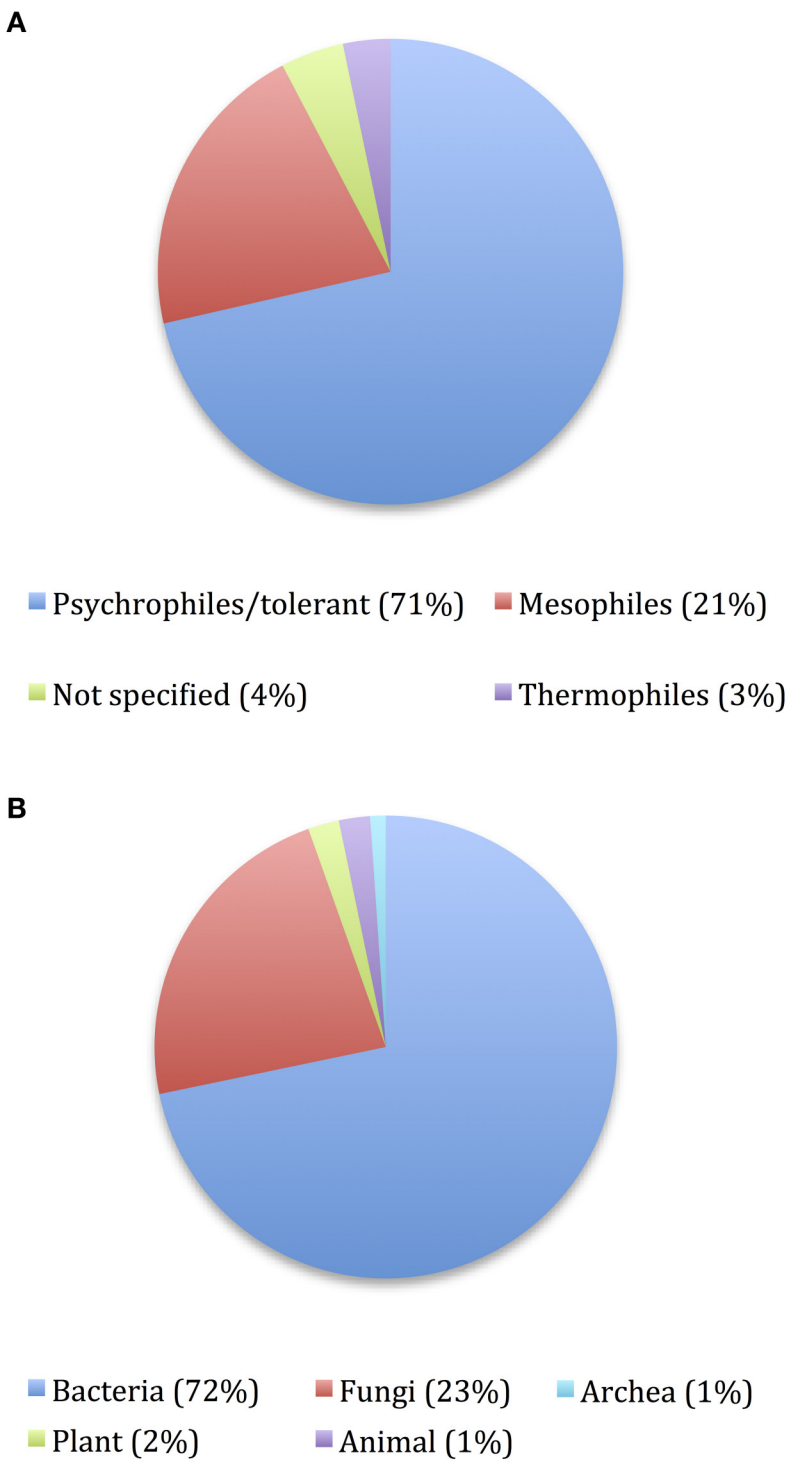
**Pie charts showing the distribution of cold-active enzymes reported in Table 1 in two different situations: (A) Nature of organism source and (B) Organism source**.

We have reviewed 92 cold-adapted enzymes that were successfully expressed in a heterologous host reported between 2010 and June 2016, which are detailed in Table 1. These enzymes were obtained mainly from psychrophilic or psychrotolerant organisms and bacteria or fungi (Figures 1A,B, respectively). These microorganisms were isolated from different and diverse environments, mainly from Polar Regions and marine environments. As explained later in this review, efficient catalysis at low temperatures requires an increase in protein flexibility, and therefore a reduction on enzyme stability. However, an interesting example of a cold-active enzyme isolated from a psycrophilic organism that had an unexpected high thermostability was reported for the superoxide dismutase DaSOD from *Deschampsia antarctica* (Rojas-Contreras et al., 2015). The optimal temperature of this enzyme is 20°C, it retains 80% of activity at 0°C and has detectable activity at −20°C, but also DaSOD possess high thermostability, its activity was not affected at 80°C, and the half-life time was 35 min at 100°C.

**Table 1:** **Source of cold-adapted enzymes microorganisms (published from 2010 to June 2016)**.

**Class**	**Enzyme**	**Origin of sample**	**Organism source**	**Molecular technique**	**Heterologous expression host**	**Expression vector**	***T*_opt_ (% residual activity at specific temperature)**	**pH_opt_**	**Kinetics parameters (substrate)**	**References**
Hydrolase	Xylanase	NS	*Flavobacterium johnsoniae*	Specific primers	*Flavobacterium johnsoniae*	Fj29	30 (50% at 4°)	8	K_m_ 8.41 mg/ml k_cat_ 17.95 s^−1^ (Birchwood)	Chen et al., 2013
Hydrolase	Esterase	Soil sample	*Pseudomonas* sp. S9	Genomic DNA library/phenotype screening/specific primers	*E. coli* TOP10	pBAD/Myc-His A	35 (40% at 10)	9	K_m_ 0.162 mM k_cat_ 3.31 s^−1^(p-NP butyrate)	Wicka et al., 2016
Hydrolase	β-galactosidase	Antarctic soil	*Paracoccus* sp. 32d	Genomic DNA library/phenotype screening/specific primers	*E. coli* LMG	pBAD/Myc -His A	40 (ND)	7.5	K_m_ 4.28 mM k_cat_ 140 s^−1^ (lactose)	Wierzbicka-Wos et al., 2011
Hydrolase	β -galactosidase	Antarctic soil	*Arthrobacter* sp. 32cB	Degenerated primers/genome walking	*E. coli* LMG194	pBAD/Myc-His A	28 (42% at 10°)	8	K_m_ 1.52 mM K_cat_ 30.55 s^−1^ (lactose)	Pawlak-Szukalska et al., 2014
Hydrolase	α-amylase	Antarctic	*Geomyces pannorum*	Degenerated primers/TAIL-PCR	*Aspergillus oryzae*	pBC12FNHA2	40 (20% at 0)	5	K_m_ 3.22 mg/ml V_max_3,33 mg/min ml (soluble starch)	Mao et al., 2015
Hydrolase	β-glucosidase	Konjac field	*Paenibacillus xylanilyticus* KJ-03	Genomic DNA library/phenotype screening/specific primers	*E. coli* BL21 (DE3)	pCold I	20 (72% at 10°)	7	K_m_ 1.19 mM k_cat_ 16.87 s^−1^ (pNPβG)	Park et al., 2013
Hydrolase	Glucanase	NS	*Eisenia fetida*	Specific primers from a related sequenced genome	*E. coli* ArcticExpress RT (DE3)	pColdI	40 (38% at 10°)	5.5	ND	Ueda et al., 2014
Hydrolase	Esterase	Marine sediment	*Microbulbifer thermotolerans* DAU221	Genomic DNA library/phenotype screening/specific primers	*E. coli* BL21 (DE3)	pColdI	46 (10% at 1)	8	K_m_ 0.099 mM V_max_ 550 μmol/min/mg (pNP-butyrate)	Lee, 2016
Hydrolase	β-galactosidase	Frozen soil	*Rahnella* sp. R3	Specific primers from a conserved region/TAIL PCR	*E. coli* BL21 (DE3)	pColdI	35 (27% at 4)	6.5	K_m_ 1.5 mM k_cat_ 3 s^−1^ (lactose)	Fan et al., 2015
Hydrolase	Lipase	Antarctic	*Psychrobacter* sp.	Specific primers	*E. coli* BL21 (DE3)	pColdI + pG-KJE8	35 (30% at 5°)	8	ND	Shuo-shuo et al., 2011
Hydrolase	Nudix hydrolase MutT	Fish	*Aliivibrio salmonicida*	Specific primers	*E. coli* BL21 AI	pDest14	12 (ND)	7.5	K_m_ 0.0029 mM k_cat_ 0.713 s^−1^ (8-oxo-dGTP)	Lian et al., 2015
Hydrolase	Inulinase	Lead-zinc-rich soil	*Arthrobacter* sp. MN	Degenerated primers/TAIL-PCR	*E. coli* BL21 (DE3)	pEASY-E1	35 (16% at 0 °C)	8	K_m_ 8.2 mM K_cat_t5.75 s^−1^ (inulina)	Zhou et al., 2015
Hydrolase	Esterase	NS	*Streptomyces coelicolor A3(2)*	Specific primers	*E. coli* BL21 (DE3)	pET16b	35 (25% at 10°)	8.5	K_m_ 2.5 mg ml^−1^ k_cat_ 0.83 s^−1^ (succinylated casein)	Brault et al., 2012
Hydrolase	Esterase	Permafrost	*Psychrobacter cryohalolentis* K5T	Specific primers	*E. coli* BL21(DE3)pLysS	pET20b	25 (70% at 5°)		ND	Petrovskaya et al., 2015
Hydrolase	β–xylanase	Marine environment	*Saccharophagus degradans* 2-40	Specific primers	*E. coli* BL21 (DE3)	pET21a	30 (ND)	7	K_m_ 10.4 mg/mL K_cat_ ND (birchwood xylan)	Ko et al., 2016
Hydrolase	Esterase	Intestine of righteye flounder	*Acinetobacter venetianus* V28	Genomic DNA library/phenotype screening/specific primers	*E. coli* BL21 (DE3)	pET22a(+)	40 (70% at 5°)	9	ND	Kim, 2012
Hydrolase	Esterase	Intestines/stomach of an Atlantic hagfish (Myxine glutinosa)	*Rhodococcus* sp. AW25M09	Specific primers	*E. coli* BL21 (DE3)	pET22b	30 (50% at 10°)	11	K_m_ 0.753 mM K_cat_ 1.63 s^−1^ (pNP-butanoate)	De Santi et al., 2014
Hydrolase	Xylanase	Marine invertebrate *Halocynthia aurantium*	*Glaciecola mesophila* KMM241	Specific primers from a related sequenced genome	*E. coli* BL21(DE3)	pET22b	35 (8% at 0°)	6	K_m_ 5.82 mg ml^−1^ k_cat_ 609 s^−1^ (Beech wood xylan)	Guo et al., 2013
Hydrolase	Esterase	Intestine of a blood clam	*Photobacterium* sp. MA1-3	Genomic DNA library/phenotype screening/specific primers	*E. coli* BL21 (DE3)	pET22b(+)	30 (45% at 5°)	8	ND	Kim et al., 2013
Hydrolase	Esterase	Intestine of silver whiting	*Salinisphaera* sp. P7-4	Genomic DNA library/phenotype screening/specific primers	*E. coli* BL21 (DE3)	pET22b(+)	25 (ND)	9	ND	Kim et al., 2011
Hydrolase	Lipase	Soil	*Sorangium cellulosum*	Specific primers	*E. coli* BL21 (DE3)	pET22b(+)	30 (35% at 0°)	8	K_m_ 0.174 mM k_cat_ 29s^−1^ (*p*-NP acetate)	Cheng et al., 2011
Hydrolase	Protease	ANTARCTIC	*Pseudoalteromonas* sp.	Degenerated primer/genome walking	*E. coli* BL21 (DE3)	pET22b(+)	25 (ND)	8	K_m_ 0.27 mM k_cat_ 199 s^−1^ (p-NP valerate)	Acevedo et al., 2013
Hydrolase	Xylanase	Soil sample	*Sorangium cellulosum* So9733-	Degenerate primers/TAIL PCR	*E. coli* BL21 (DE3)	pET22b(+)	30–35°C (13.7% at 0°C)	7	K_m_ 25.77 mg/ml k_cat_ 6.84 s^−1^ (Beechwood xylan)	Wang S. Y. et al., 2012
Hydrolase	α-glucosidase	Culture collection from Anhui University	*Pseudoalteromonas* sp. K8	Degenerated primers	*E. coli* BL21 (DE3)	pET22b(+)	30 (30% at 0°)	8.5	K_m_ 0.27 mM k_cat_ 15 s^−1^ (pNPαG)	Li et al., 2016b
Hydrolase	Lipase	Antarctic seawater	*Shewanella frigidimarina* NCIMB 400	Degenerated primers	*E. coli* BL21 (DE3)	pET22b(+)	25 (35% at 10°)	8	ND	Parra et al., 2015
Hydrolase	Protease	Antarctic seawater	*Pseudoalteromonas haloplanktis* TAC125	Protein sequence/specific primers	*E. coli* BL21 (DE3)	pET22b(+)	15 (20% at 5°)	8	ND	de Pascale et al., 2010
Hydrolase	Xylanase	DNA of goat rumen fluid	–	Degenerate primer/TAIL PCR	*E. coli* BL21 (DE3)	pET22b(+)	30 (10% at 0°)	6.5	K_m_1.8 mg ml^−1^ k_cat_ 584 s^−1^ (Beechwood xylan)	Wang et al., 2011
Hydrolase	β-galactosidase	NS	*Pyrococcus furiosus*	Specific primers	*E. coli* BL21 (DE3)	pET24a(+)	90 (8% at 0°)	7	ND	Dong et al., 2014
Hydrolase	Esterase	Sea floor	*Thalassospira* sp. GB04J01	Specific primers	*E. coli* BL21 (DE3)	pET26b	45 (20% at 10)	8.5	K_m_ 0.94 mM k_cat_ 47.7 s^−1^ (pNP-acetate)	De Santi et al., 2016
Hydrolase	Pullulanase	Soil sample	*Exiguobacterium* sp. *SH3*	Specific primers	*E. coli* BL21 (DE3)/*B. subtilis* WB600	pET26b(+) pHY300PLK	45 (30% at 10)	8.5	K_m_ 2.8 mg/ml K_cat_t37s^−1^ (pullulan)	Rajaei et al., 2015
Hydrolase	Esterase	NS	*Pseudomonas mandelii*	Specific primers	*E. coli* BL21 (DE3)	pET28a	40 (ND)	8.5	K_m_ 0.21 mM k_cat_ 3.4 s^−1^ (*p*-NP acetate)	Lee et al., 2013
Hydrolase	Xylanase	Sediment sample from a soda lake	*Bacillus* sp. SN5	Genomic DNA library/phenotype screening/specific primers	*E. coli* BL21 (DE3)	pET28a	40 (29% at 5°)	7	K_m_ 0.6 mg/ml k_cat_ ND (beechwood xylan)	Bai et al., 2012
Hydrolase	β-glucosidase	Antarctic soil	*Exiguobacterium antarcticum* B7	Specific primers	*E. coli* Rosetta	pET28a	30 (25% at 5°)	7	K_m_ 1.07 mM k_cat_ 32.98s^−1^ (pNPβG)	Crespim et al., 2016
Hydrolase	Pullulanase	Soil of fruit market garbage dump	*Paenibacillus polymyxa* Nws-pp2	Degenerated primers	*E. coli* BL21(DE3)	pET28a	35 (40% at 10°)	6	K_m_ 15.25 mg/ml V_max_20.1 U/mg (pullulan)	Wei et al., 2015
Hydrolase	Glycogen branching enzyme	CGMCC	*Rhizomucor miehei*	Degenerate primers/RACE PCR	*E. coli* BL21 (DE3)	pET28a (+)	25 (ND)	7.5	ND	Wu et al., 2014
Hydrolase	Lipase	Deep-sea sediments	*Psychrobacter* sp. *C18*	Genomic DNA library/phenotype screening/specific primers	*E. coli* BL21 (DE3)	pET28a(+)	30 (18% at 0°)	8	ND	Chen et al., 2010
Hydrolase	β-mannanase	Slag of a phosphate rock-stacking site	*Sphingomonas* sp. JB13	Degenerate primer/TAIL-PCR	*E. coli* BL21 (DE3)	pET28a(+)	40 (20% at 10°C)	6.5	K_m_ 5 mg ml^−1^ k_cat_ 211.9 s^−1^ (locust bean gum)	Zhou et al., 2012
Hydrolase	Endoglucanase	Lake sediment	*Paenibacillus* sp. *IHB B 3084*	Specific primers	*E. coli* BL21 (DE3)	pET28a(+)	40 (70% at 5°)	5	K_m_ 40.5 mg/ml V_max_ 0.692 IU/ml (CMC)	Dhar et al., 2015
Hydrolase	Esterase	Sediment of soda lake	*Alkalibacterium* sp. SL3	TAIL-PCR	*E. coli* BL21 (DE3)	pET28a(+)	30 (70% at 0°)	9	K_m_ 0.15 mM k_cat_ 307.69s^−1^ (pNP-acetate)	Wang et al., 2016
Hydrolase	β-glucanase	Deep-sea sediment	*Pseudomonas* sp. MM15	Genomic DNA library/phenotype screening/specific primers	*E. coli* BL21 (DE3)	pET28a+	30 (70% at 10°)	4.5	ND	Yang and Dang, 2011
Hydrolase	β-amylase	NS	*Arabidopsis thaliana*	Specific primers	*E. coli* BL21 (DE3)	pET29a	30 (20% at 0°)	6	ND	Monroe et al., 2014
Hydrolase	Lipase	Glacier soil	Acinetobacter sp. XMZ-26	Degenerated/genome walking	*E. coli* BL21 (DE3)	pET30a(+)	15 (39% at 0°)	10	K_m_0.075 mM k_cat_ 561s^−1^ (*p*-NP octanoate)	Zheng et al., 2011
Hydrolase	β-glucosidase	Gut of longhorned beetle (Batocera horsfieldi) larvae	*Serratia* sp. TN49	Degenerate primer/TAIL-PCR	*E. coli* BL21 (DE3)	pET30a(+)	35 (25% at 10°)	7.5	K_m_ 7.79 mM k_cat_ 22.6 s^−1^ (pNPG)	Zhou et al., 2011
Hydrolase	Lipase	CGMCC	*Stenotrophomonas maltophilia* GS11	Specific primers	*E. coli* BL21 (DE3)	pET30a(+)	35 (55% at 5)	8	ND	Li et al., 2016a
Hydrolase	Lipase	Siberian cryopeg	*Psychrobacter cryohalolentis K5*	Specific primers	*E. coli* BL21 (DE3)	pET32a	25 (60% at 5°)	8.5	ND	Novototskaya-Vlasova et al., 2013b
Hydrolase	β-mannanase	Soil	*Bacillus subtilis* Bs5	Specific primers from a related sequenced genome	*E. coli* Rosetta_gami (DE3)	pET32a	35 (ND)	5	ND	Huang et al., 2012
Hydrolase	Esterase	Siberian permafrost	*Psychrobacter cryohalolentis K5*T**	Specific primers	*E. coli* BL21 (DE3)	pET32a(+)	35 (82% at 0°)	8.5	ND	Novototskaya-Vlasova et al., 2012
Hydrolase	Lipase	Siberian cryopeg	*Psychrobacter cryohalolentis K5*	Specific primers	*E. coli* BL21 (DE3)	pET32a(+)	25 (80% at 5°)	9	ND	Novototskaya-Vlasova et al., 2013a
Hydrolase	Esterase	Seawater	*Photobacterium* sp. *strain J15*	Degenerated primers	*E. coli* Rosetta-gami (DE3) pLysS	pET32b(+)	20 (50% at 4)	8	ND	Shakiba et al., 2016
Hydrolase	Alkaline phosphatase	Mantle tissue of the marine mussel	*Cobetia marina*	Specific primers	*E. coli* Rosetta (DE3)	pET40b (+)	40 (ND)	9.5	K_m_ 0.3 mM K_cat_t24,000 s^−1^ (pN-phosphate)	Golotin et al., 2015
Hydrolase	α-galactosidase	Marine environment	*Pseudoalteromonas* sp. *KMM 701*	Specific primers	*E. coli* Rosetta(DE3)	pET40b(+)	20 (ND)	7	K_m_ 0.412 mM k_cat_ 0.588 s^−1^ (pNP-αGal)	Bakunina et al., 2014; Balabanova et al., 2010
Hydrolase	Lipase	Dirty and cool tream water	*Pseudomonas* sp. TK-3	Genomic DNA library/phenotype screening/specific primers	*E. coli* BL21 (DE3)	pET47b	20 (30% at 5 °C)	8	ND	Tanaka et al., 2012
Hydrolase	Protease	NS	S*hewanella arctica*	Genomic DNA library/phenotype screening/specific primers	*E. coli* Tuner (DE3) pLacl	pETBlue1	60 (20% at 0°)	8	K_m_ 0.175% (w/v) k_cat_ 5.186 s^−1^ (casein)	Qoura et al., 2015
Hydrolase	Lipase	NS	*Candida albicans*	Specific primers	*P. pastoris*	pGAPZaA	15 (50% at 5°)	5	K_m_0.27 mM k_cat_ 551 s^−1^ (*p*-NP caprylate)	Lan et al., 2011
Hydrolase	β-mannosidase	NS	*Aspergillus niger* CBS 513.88	Synthetized from known sequence	*P. pastoris* X33	pGAPzaA	45 (22% at 0°)	5	K_m_ 2.87 mg/ml k_cat_ 492.29 s^−1^ (guar gum)	Zhao W. et al., 2011
Hydrolase	Lipase	NS	*Malassezia globose*	Synthetized from known sequence	*P. pastoris X-33*	pGAPZαA	15 (50% at 5 °C)	6	ND	Xu et al., 2015
Hydrolase	Lipase	NS	*Bacillus* sp.	Genomic DNA library/phenotype screening/specific primers	*E. coli* JM109	pGEM-T	35 (55% at 10°)	8	K_m_ 3.3 mM K_cat_t2.4 x 10^−5^ s^−1^ (pNP laurate)	Khurana et al., 2015
Hydrolase	β-galactosidase	NS	*Lactococcus lactis*	Specific primers	*E. coli* NovaBlue (DE3)	pGEMT-Easy	15-55 (60% at 5°)	6-7.5	K_m_ 0.82 mM k_cat_ 102 s^−1^ (lactose)	Vincent et al., 2013
Hydrolase	Phytase	NS	*Bacillus licheniformis*	Specific primers	*E. coli* BL21 (DE3)	pGEMT-Easy Vector	75 (40% at 4°)	7	K_m_ 178 μM K_cat_ 1163.5 s^−1^ (phytic acid)	Borgi et al., 2014
Hydrolase	α-amylase	Surface seawater	*Zunongwangia profunda*	Specific primers	*E. coli* BL21 (DE3)	pGEX-6P-1	35 (39% at 0°)	7	K_m_ 2.3 mM K_cat_ 329.58 s^−1^ (soluble starch)	Qin et al., 2014
Hydrolase	Esterase	Deep seawater	*Psychrobacter pacificensis*	Genomic DNA library/phenotype screening/specific primers	*E. coli* BL21 (DE3)	pGEX-6p-1	25 (70% at 10 °C)	7.5	K_m_ 0.034 mM K_cat_t5.75 s^−1^ (p-NP butyrate)	Wu et al., 2015
Hydrolase	Esterase	Deep-sea sediments	*Psychrobacter celer* 3Pb1	Genomic DNA library/phenotype screening/specific primers	*E. coli* BL21 (DE3)	pGEX-6p-1	35 (41% at 0 °C)	7.5	K_m_ 0.033 mM k_cat_ 9.21 s^−1^ (p-NP butyrate)	Wu et al., 2013b
Hydrolase	Esterase	Sediments in the Gulf of Mexico	*Psychrobacter pacificensis*	Genomic DNA library/phenotype screening/specific primers	*E. coli* BL21 (DE3)	pGEX-6p-1	25 (55% at 0°C)	7.5	K_m_ 0.7667 mM k_cat_ 3.92 s^−1^ (p-NP butyrate)	Wu et al., 2013a
Hydrolase	Xylanase	Seawater	*Zunongwangia profunda*	Specific primers	*E. coli* BL21 (DE3)	pGEX-6p-1	30 (23% at 0°)	6.5	K_m_ 1.15 mg/ml K_cat_ 80.33 s^−1^ (beechwood xylan)	Liu et al., 2014
Hydrolase	Esterase	Marine environment	*Serratia* sp.	Specific primers from a related sequenced genome	*E. coli* BL21 (DE3)	pGEX-6P-1	10 (92% at 0)	8.5	K_m_ 0.074 mM k_cat_ 2339 s^−1^ (pNP-acetate)	Jiang et al., 2016
Hydrolase	Esterase	Surface seawater	*Zunongwangia profunda*	Specific primers	*E. coli* BL21 (DE3)	pGEX-6P-1	30 (75% at 0)	8	K_m_ 0.121 mM K_cat_ 110 s^−1^ (pNP-butyrate)	Rahman et al., 2016
Hydrolase	β-galactosidase	Antarctica deep lake	*Halorubrum lacusprofundi*	Specific primers	*Halobacterium* sp. NRC-1	pKJ408	50 (10% at 0°)	6.5	ND	Karan et al., 2013
Hydrolase	Trypsin	Antarctic	*Euphausia superba*	Peptide sequence/degenerated and specific primres/RACE PCR	*E. coli TB1*	pMAL-c2E	50 (ND)	9	K_m_ ND k_cat_ 6 s-1 (BAPNA)	Olivera-Nappa et al., 2013
Hydrolase	Lipase	Antarctic	*Penicillium expansum*	Degenerated primers/genome walking	*E. coli Origami B (DE3)*	pMAL-c5E	10 (ND)	8	ND	Mohammed et al., 2013
Hydrolase	Chitosanase	Fresh water lake	*Janthinobacterium* sp. strain 4239	Genomic DNA library/phenotype screening/specific primers	*E. coli* DH10B	pMGJ1042	45 (30% at 10°)	5	ND	Johnsen et al., 2010
Hydrolase	Xylanase	Beech stump	*Bispora antennata*	Degenerated primers/TAIL-PCR	*P. pastoris* (GS115)	pPIC9	35 (21% at 0°)	5.5	K_m_ 1.65 mg/ml V_max_ 236 mmol/min/mg (birchwood xylan)	Liu et al., 2015
Hydrolase	Pectin methylesterase	Wastewater of food processing	*Penicillium chrysogenum* F46	Specific primers from a related sequenced genome	*P. pastoris* GS115	pPIC9	40 (52% at 10°)	5	K_m_ 0.55 mg/ml V_max_ 15.78 mmol/min/mg (pectin)	Pan et al., 2014
Hydrolase	Polygalacturonase	Desert sand	*Achaetomium* sp. Xz8	Degenerate primers/TAIL-PCR	*P. pastoris* GS115	pPIC9	45 (10% at 0°)	6	K_m_ 0.32 g/l V_max_ 97,951 mmol/min/mg (polygalacturonic acid)	Tu et al., 2013
Hydrolase	Lipase	CGMCC	*Rhizomucor endophyticus*	Degenerated primers/RACE	*P. pastoris* GS115	pPIC9 K	40 (75% at 0)	6	K_m_ 2.3 mM k_cat_ 0.891 s^−1^ (pNP-caprylate)	Yan et al., 2016
Hydrolase	Lipase	NS	*Candida Parapsilosis*	Specific primers	*P. pastoris* GS115	pPIC9K	35 (45% at 5°)	6.5	ND	Neang et al., 2014
Hydrolase	Lipase	NS	*Candida tropicalis*	Specific primers	*P. pastoris* GS115	pPIC9K	45 (36% at 5°)	6.5	ND	Neang et al., 2014
Hydrolase	Pullulanase	Sea water	*Shewanella arctica*	Genomic DNA library/phenotype screening/specific primers	*E. coli* M15	pQE-30	35 (25% at 10)	7	K_m_ 0,1% K_cat_ 86,9 s^−1^ (pullulan)	Elleuche et al., 2015
Hydrolase	Lipase	Soil at a car service area	*Staphylococcus epidermidis* AT2	Specific primers	*E. coli* (DE3) pLacI	pTrcHis2-TOPO	25 (ND)	8	ND	Kamarudin et al., 2014
Hydrolase	β−galactosidase	Artic	*Alkalilactibacillus ikkense*	Genomic DNA library/phenotype screening/specific primers	*E. coli* TOP10	pUC18	20 (60% at 0°)	8	ND	Schmidt and Stougaard, 2010
Hydrolase	Esterase	Human saliva	*Lactobacillus plantarum* WCFS1	Specific primers	*E. coli* BL21 (DE3)	pURI3-TEV + pGRO7	5 (ND)	6	ND	Esteban-Torres et al., 2014b
Hydrolase	Esterase	Human saliva	*Lactobacillus plantarum* WCFS1	Specific primers	*E. coli* BL21 (DE3)	pURI3-TEV + pGRO7	20 (90% at 5°)	6.5	ND	Esteban-Torres et al., 2014a
Hydrolase	Protease	Compost	*Bacillus* sp. B001	Degenerate primers/genomic DNA digestion and self-ligation/reverse PCR	*B. subtilis* WB600	pWB980	60 (ND)	10	K_m_0.44 mM k_cat_ 4181 s^−1^ (casein)	Deng et al., 2011
Hydrolase	Lipase	Antarctic soil	*Geomyces* sp. *P7*	Inverse PCR	*S. cerevisiae* (BJ5465)	pYES 2.1	35 (15% at 0°)	8	K_m_ 8.5 mM k_cat_ 118s^−1^ (*p*-NP acetate)	Florczak et al., 2013
Hydrolase	β-glucosidase	Root surface of the salt marsh grass *Spartina anglica*	*Marinomonas* MWYL1	Synthetized from known sequence	*E. coli* DH5a	pYPX251	40 (20% at 5°C)	7	K_m_ 0.9 mg ml^−1^ k_cat_ 475.4 s^−1^ (oNPGlc)	Zhao W. et al., 2012
Isomerase	Arabinose isomerase	Provided by other laboratory	*Shewanella* sp. ANA-3	Specific primers	*E. coli* BL21 (DE3)	pET15b	15 (90% at 4°)	5.5-6.5	K_m_ 33.7 mM V_max_ 164 mmole/s/mg (L-arabinose)	Rhimi et al., 2011
Ligase	Glutathione synthetase	Antarctic sea	*Pseudoalteromonas haloplanktis*	Specific primers	*E. coli* BL21 (DE3)	pET28a(+)	15 (ND)	7.8	K_m_ 0.25 mM k_cat_ 1.93s^−1^ (γ-glutamylcysteine)	Albino et al., 2012
Oxidoreductase	Superoxide dismutase	Antarctic	*Deschampsia antarctica*	Specific primers	*E. coli* BL21-SI	NS	20 (80% at 0°)	7	ND	Rojas-Contreras et al., 2015
Oxidoreductase	Nitroreductase	Urinary tract	*Staphylococcus saprophyticus*	Specific primers	*E. coli* BL21 (DE3)	pET14b	20 (80% at 3°)	7.5	K_m_ 0.0498 mM k_cat_ 2.2 s^−1^ (NFZ)	Çelik and Yetis, 2012
Oxidoreductase	Glutaredoxin	Antarctic sea ice	*Pseudoalteromonas* sp. *AN178*	Specific primers for Grx from the genera	*E. coli* BL21 (DE3)	pET28a (+)	30 (25.5% at 0°C)	8	K_m_ 0.46mM V_max_ 14.3 nmol/mL/min (HED)	Wang Q. et al., 2014
Oxidoreductase	Glycine oxidase	Marine sediment sand	*Bacillus lichentformis*	Specific primers	*E. coli* BL21 (DE3)	pGEX-6p-1	40 (60% at 0°)	8.5	K_m_ 11.22 mM k_cat_ 0.08 s^−1^ (glyphosate)	Zhang et al., 2016
Transferase	Serine hydroxymethyl transferase	Arctic polar sea ice	*Psychromonas ingrahamii*	Synthesized from known sequence	*E. coli HMS174 (DE3)*	pET28a	30 (ND)	7.2	K_m_ 1, 6 mM k_cat_ 1.78 s^−1^ (L-allo-threonine)	Angelaccio et al., 2012
Transferase	Glutathione S-transferase	Antarctic sea ice	*Pseudoalteromonas* sp. ANT506	Degenerated primers	*E. coli* BL21 (DE3)	pET28a (+)	40 (14.2% at 0°)	7	K_m_ 1.01 mM K_cat_ ND (glutathione)	Shi et al., 2014

BAPNA, Nα-benzoyl-L-arginine 4-nitroanilide; CGMCC, China General Microbiological Culture Collection Center; CMC, carboxymethyl cellulose; dGTP, Deoxyguanosine triphosphate; HED, hydroxyethyl disulfide; ND, not determined; NS, not specified; pNP, p-nitrophenol; pNP-αGal, p-nitrophenyl-α-D-galactopyranoside; oNPGlc, 2-Nitrophenyl-b-D –glucopyranoside; pNPαG, 4-Nitrophenyl-α-D-glucopyranoside; pNPβG, 4-Nitrophenyl β-D-glucopyranoside; NFZ, nitrofurazone; RACE, Rapid amplification of cDNA ends; TAIL-PCR, Thermal Asymmetric Interlaced PCR.

There are good examples of cold-active enzymes isolated from mesophilic organisms. Most of the time, a high activity at low temperatures is unexpected during the characterization of the catalytic properties of a mesophilic enzyme. This was the case of a *Candida albicans* lipase (Lan et al., 2011), which shows a low sequence identity with those of known lipases from psychrophilic organisms, but has an optimal temperature of 15°C. Other example is the lipase from *Staphylococcus epidermidis*, isolated from a car service area, with an optimal temperature of 25°C (Kamarudin et al., 2014). Other interesting case was reported by Monroe et al. (2014), where the cold-active properties from β-amylase 3 from *Arabidopsis* were inferred from the fact that this enzyme was more active during nighttime, compared to β-amylase 1 that had the opposite behavior. Both enzymes were overexpressed and purified from *Escherichia coli* confirming that they were differentially thermal adapted. β-amylase 3 had a lower optimal temperature, greater residual activity at low temperatures and less thermal stability than β-amylase 1.

More surprising is to discover a thermophilic enzyme with high activity at low temperatures. This was the case of a β-galactosidase isolated from *Pyrococcus furiosus* (Dong et al., 2014) with optimal activity at 90°C (130 U/mg). The enzyme was still active at 0°C, retaining 8% of its activity. Despite the decrease in activity compare to its optimal temperature, the lactase activity of *P. furiosus* at 0°C was still 40% of the optimal activity from the main β-galactosidase use in the food industry (28 U/mg at 50°C and pH 7.0) from *K. marxianus*. In addition, the lactase activity of *P. furiosus* at 0°C was 31% of the optimal activity of a cold-active β-galactosidase from *Arthrobacter psychrolactophilus* strain F2 (33 U/mg at 10°C and pH 8.0).

## Gene cloning and recombinant expression systems for cold-active enzymes

The usual approach to obtain sufficient enzyme yield for purification, characterization, and final use consists of the recombinant expression of enzymes in a heterologous host. Mesophilic hosts are the most commonly used systems for heterologous expression of genes encoding cold-active enzymes (Table 1). However, the optimal growth temperature of these microorganisms is not compatible with the temperature that cold-active enzymes need to properly fold in order to retain their structure and functional activity (Bjerga et al., 2016). One alternative to circumvent these folding issues in *E. coli* is to lower the incubation temperatures of the cell culture to 18°C after induction (Feller et al., 1998), although this also decreases the host growth rate and thus the synthesis rate of heterologous enzyme is also reduced. Here, we briefly summarize the standard strategies for the expression of cold-active enzymes, which have been largely used for most of the enzymes reviewed in Table 1, followed by a more extensive revision of novel strategies for improving the expression of cold-active enzymes aiming to enhance their solulibility, protein yield, and proper folding.

The starting point of most of the reviewed enzymes was the isolation of a cold-adapted organism with an interesting enzymatic activity. The main cloning strategy was the design of specific primers for gene amplification using the genomic DNA of the strain as template, (~48% of enzymes in Table 1). This is only possible if the genome of the species (or a very close relative) has been sequenced or the gene has been deposited in Gene Bank, and also if the microorganism can be properly cultured in order to obtain its genomic material. If the organism is not available or impossible to grow, the alternative is to synthesize the gene with an optimal codon usage for the host; this was the case of four cold-adapted enzymes described in Table 1 (Zhao W. et al., 2011, 2012; Angelaccio et al., 2012; Xu et al., 2015).

When the gene sequences were not available, the preferred cloning strategy was the creation of a genomic library, with subsequent clone screening, followed by sequencing the candidate clone to finally obtain a sequence that can be inserted into an expression vector (~21% of enzymes in Table 1). Degenerated primers for partial gene amplification, complemented with TAIL PCR, genome walking, RACE or inverse PCR, were used to a lesser extent.

**Figure 2:**
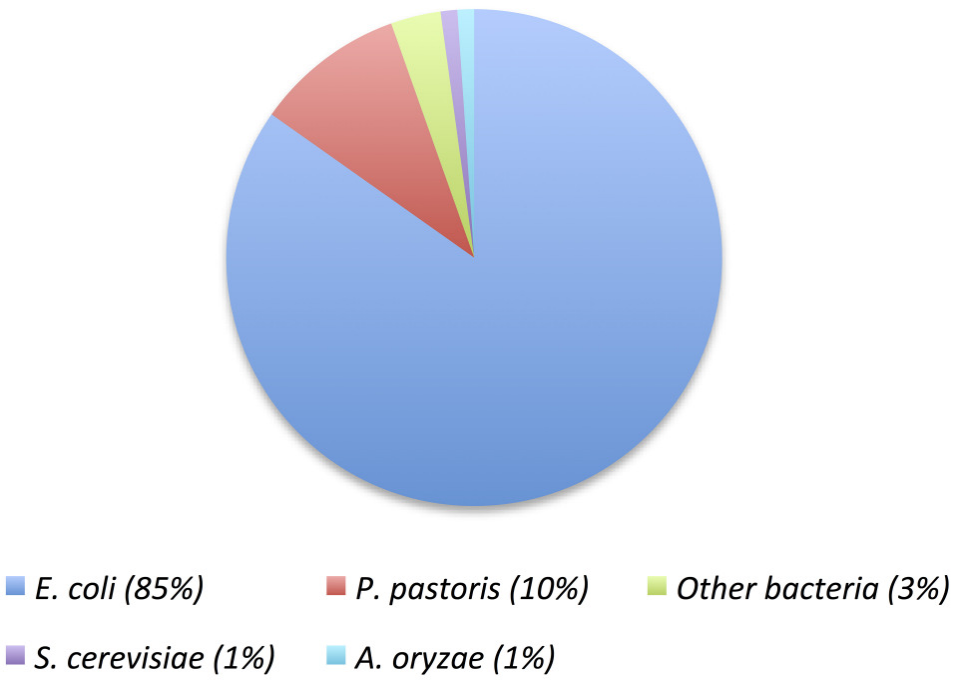
**Pie chart showing the distribution of heterologous hosts used for the expression of cold-active enzymes reported in Table 1**.

The selected expression host was by far *E. coli* (Figure 2). Different genotypes were used, but in most cases BL21 (DE3) was the preferred strain. As we will see below, only one of these enzymes was expressed in an optimized strain for cold-active enzymes, ArcticExpress. Nevertheless, other expression hosts have been used, such as *Halobacteriun* sp. for the expression of a cold-adapted hydrolase, and *Pichia pastoris*, used as the expression host for 9 proteins including various fungal enzymes. Other expression hosts that were rarely used are shown in Table 1.

**Figure 3:**
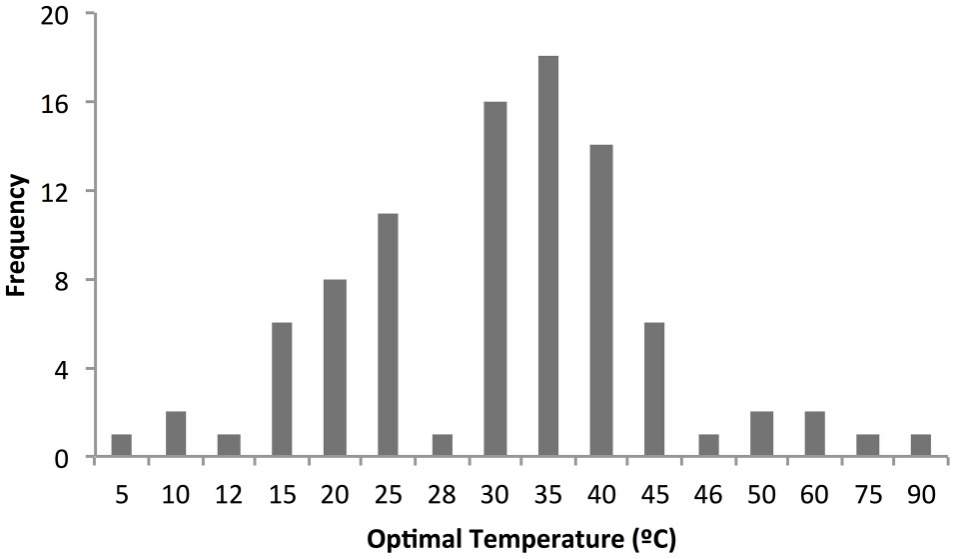
**Graphical representation of the distribution of the optimal temperatures of cold-active enzymes**. The optimal temperature reported for enzymes from Table 1 is represented in a frequency plot noticing that temperatures are distributed between 5 and 90°C and the majority of the enzymes have a T_opt_ between 20 and 45°C.

Half of the cold-adapted genes were cloned in plasmids from the pET system for their expression. Only five of the genes were cloned in pCold vectors, whose advantages are described later in this review. Fusion constructs were also used for cloning 10 genes, eight in pGEX-6P-1, which allow the fusion expression of proteins to GST, and two in pMAL-c, which express proteins fusion to MBP. Other vectors are detailed in Table 1. Concerning enzyme purification, for more than half of the enzymes from Table 1 the purification process was aided by fusion to a His tag. The majority of the enzymes were overproduced in the cytoplasm in a soluble form (72%). Only 15% were secreted and 8% were insoluble. Only two enzymes were purified from the periplasm and one was expressed in the outer membrane through fusion with an autotransporter domain (Petrovskaya et al., 2015; Table 1). Almost all enzymes were characterized, providing data from their optimal temperature (T_opt_), optimal pH (pH_opt_) and kinetic parameters like *k*_cat_ and *K*_m_. The distribution of the optimal temperatures of the enzymes is displayed in Figure 3, and shows that T_opt_ are distributed between 5 and 90°C, with 80% of the enzymes having a T_opt_ between 20 and 45°C.

Is important to underline that for *in vitro* characterization of enzymes, T_opt_ is obtained by measuring the enzyme activity at fixed temperatures and conditions, so it is likely that these numbers provide an approximate value for T_opt_. Nevertheless, the distribution of T_opt_ displayed in Figure 3 has a fundamental meaning, as this parameter often reflects the temperature of the environmental niches inhabited by their source organisms: albeit their source organisms are either psychrophilic or psychrotolerant (Figure 1A), the ability of these enzymes to remain active in the cold is the result of either complete or incomplete evolutionary adaptations of their structure and sequence for functioning at low temperatures, with enzymes from psychrotolerant being often identified as examples of incomplete evolution (Georlette et al., 2004). Regardless of the degree of completeness of their cold-adaptations, these enzymes are evidently cold-active, as demonstrated by the retention of an important percentage of their activity between 0 and 10°C for almost all of the enzymes in Table 1.

**Figure 4:**
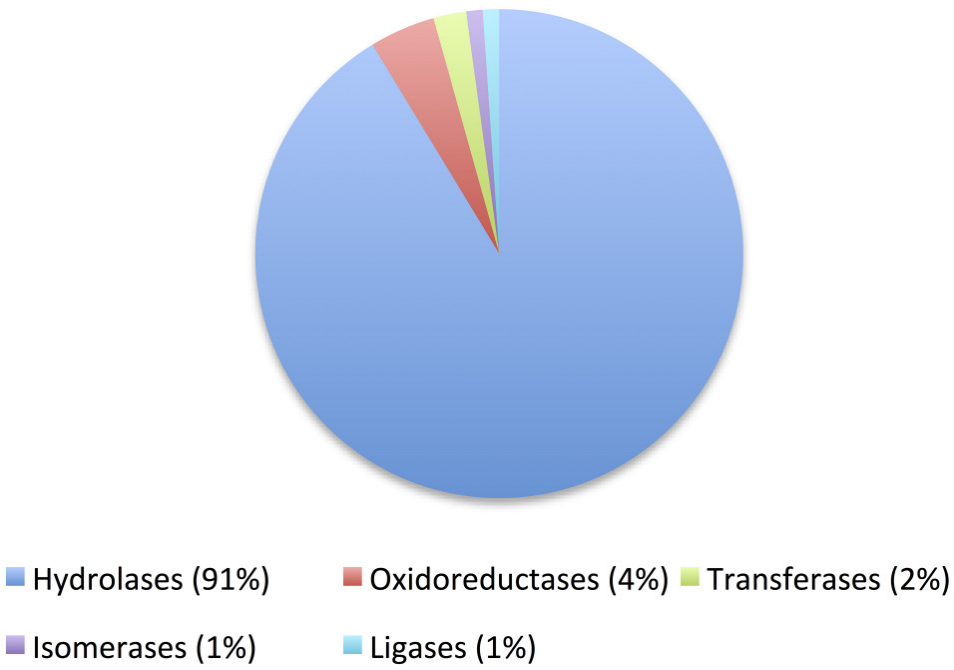
**Pie chart showing the distribution of enzymes classes of cold-active enzymes reported in Table 1**.

By far hydrolases were the preferred class for cold-enzyme discovery (Figure 4). Unsurprisingly, cold-adapted hydrolases are the most frequent proteins for which their three-dimensional structures have been solved (Table 2). Among them, lipases and esterases were the favorites (18 and 20% of enzymes in Table 1, respectively), which is the same case reported recently for cold-active enzymes obtained by metagenomic approaches where all the proteins were hydrolases (30% lipases and 30% esterases) except one (Vester et al., 2015).

**Table 2:** **Solved structures of cold adapted enzymes deposited in the Protein Data Bank**.

**Enzyme**	**Classification**	**Source organism**	**PDB ID**	**References**
Alkaline phosphatase	Hydrolase	*Shewanella* sp.	3A52	Tsuruta et al., 2010
Alkaline phosphatase	Hydrolase	*Vibrio* sp.	3E2D	Helland et al., 2009
Alkaline phosphatase	Hydrolase	Antartic bacterium TAB5	2IUC	Wang et al., 2007
Alkaline phosphatase	Hydrolase	*Pandalus borealis*	1K7H	de Backer et al., 2002
Amidase	Hydrolase	*Nesterenkonia* sp.	3HXK	Nel et al., 2011
Aminopeptidase	Hydrolase	*Colwellia psychrerythraea*	3CIA	Bauvois et al., 2008
Cellulase	Hydrolase	*Pseudoalteromonas haloplanktis*	1TVN, 1TVP	Violot et al., 2005
Chitinase	Hydrolase	*Moritella marina*	4MB3, 4MB4, 4MB5	Malecki et al., 2013
Elastase	Hydrolase	*Salmo salar*	1ELT	Berglund et al., 1995
Endonuclease I	Hydrolase	*Aliivibrio salmonicida*	2PU3	Altermark et al., 2008
Esterase	Hydrolase	*Thalassospira* sp.	4V2I	De Santi et al., 2016
Esterase	Hydrolase	Arctic metagenomic library	4AO6	Fu et al., 2013
Esterase	Hydrolase	*Oleispira antarctica*	3I6Y, 3S8Y	Lemak et al., 2012
Esterase	Hydrolase	*Pseudoalteromonas* sp.	3HP4	Brzuszkiewicz et al., 2009
Lipase	Hydrolase	*Proteus mirabilis*	4GW3, 4GXN	Korman and Bowie, 2012
Lipase	Hydrolase	*Photobacterium lipolyticum*	2ORY	Jung et al., 2008
Lysozyme	Hydrolase	*Bombyx mori*	1GD6	Matsuura et al., 2002
Pepsin	Hydrolase	*Gadus morhua*	1AM5	Karlsen et al., 1998
Peptidase	Hydrolase	*Serratia* sp.	2B6N	Helland et al., 2006
Protease	Hydrolase	*Flavobacterium* sp.	3U1R	Zhang et al., 2011
Protease	Hydrolase	*Pseudomonas* sp.	1G9K, 1H71	Aghajari et al., 2003
Protein tyrosine phosphatase	Hydrolase	*Shewanella* sp.	1V73	Tsuruta et al., 2005
Pyrophosphatase	Hydrolase	*Oleispira antarctica*	3I4Q	Kube et al., 2013
S-formylglutathione hydrolase	Hydrolase	*Pseudoalteromonas haloplanktis*	3LS2	Alterio et al., 2010
Serine protease	Hydrolase	*Bacillus subtilis*	2GKO	Almog et al., 2009
Serine protease	Hydrolase	*Vibrio* sp.	1S2N, 1SH7	Arnórsdóttir et al., 2005
Trypsin	Hydrolase	*Oncorhynchus keta*	1MBQ	Toyota et al., 2002
Trypsin	Hydrolase	*Salmo salar*	2TBS	Smalås et al., 1994
Uracil-DNA N-glycosylase	Hydrolase	*Gadus morhua*	1OKB	Leiros et al., 2003
Xylanase	Hydrolase	*Aegilops speltoides*	5AY7, 5D4Y	Zheng et al., 2016
Xylanase	Hydrolase	*Pseudoalteromonas haloplanktis*	1H12, 1H13, 1H14	Van Petegem et al., 2003
α-amylase	Hydrolase	*Pseudoalteromonas haloplanktis*	1B0I	Aghajari et al., 1998
β-galactosidase	Hydrolase	*Arthrobacter* sp.	1YQ2	Skalova et al., 2005
β-glucanase	Hydrolase	*Eisenia fetida*	3WC3	Arimori et al., 2013
β-glucosidase	Hydrolase	*Exiguobacterium antarcticum*	5DT5, 5DT7	Zanphorlin et al., 2016
β-glucosidase	Hydrolase	*Micrococcus antarcticus*	3W53	Miao et al., 2016
β-lactamase	Hydrolase	*Pseudomonas fluorescens*	2QZ6	Michaux et al., 2008
Prolyl isomerase	Isomerase	*Cenarcheaum symbiosum*	2RQS	Jaremko et al., 2011
Sedoheptulose 7-phosphate isomerase	Isomerase	*Colwellia psychrerythraea*	5BY2	Do et al., 2015b
Triose phosphate isomerase	Isomerase	*Moritella marina*	1AW1, 1AW2	Alvarez et al., 1998
3-octaprenyl-4-hydroxybenzoate carboxylase	Lyase	*Colwellia psychrerythraea*	4RHE, 4RHF	Do et al., 2015a
Citrate synthase	Lyase	*Arthrobacter* sp.	1A59	Russell et al., 1998
Ectoine synthase	Lyase	*Sphingopyxis alaskensis*	5BY5, 5BXX	Widderich et al., 2016
Tryptophan synthase	Lyase	*Shewanella frigidimarina*	3VND	Mitsuya et al., 2014
Catalase	Oxidoreductase	*Aliivibrio salmonicida*	2ISA	Riise et al., 2007
Ectoine hydroxylase	Oxidoreductase	*Sphingopyxis alaskensis*	4Q5O, 4MHR, 4MHU	Höppner et al., 2014
Isocitrate dehydrogenase	Oxidoreductase	*Desulfotalea psychrophila*	2UXQ, 2UXR	Fedøy et al., 2007
L-leucine dehydrogenase	Oxidoreductase	*Flavobacterium frigidimaris*	2YY7	Yoneda et al., 2010
Lactate dehydrogenase	Oxidoreductase	*Champsocephalus gunnari*	2V65	Coquelle et al., 2007
Leucine dehydrogenase	Oxidoreductase	*Sporosarcina psychrophila*	3VPX	Zhao Y. et al., 2012
Malate dehydrogenase	Oxidoreductase	*Aquaspirillium arcticum*	1B8P, 1B8U, 1B8V	Kim et al., 1999
Phenylalanine hydroxylase	Oxidoreductase	*Colwellia psychrerythraea*	2V27, 2V28	Leiros et al., 2007
Superoxide dismutase	Oxidoreductase	*Pseudoalteromonas haloplanktis*	3LJF, 3LJ9, 3LIO	Merlino et al., 2010
Superoxide dismutase	Oxidoreductase	*Aliivibrio salmonicida*	2W7W	Pedersen et al., 2009
Adenylate kinase	Transferase	*Marinibacillus marinus*	3FB4	Davlieva and Shamoo, 2009
Adenylate kinase	Transferase	*Sporosarcina globispora*	1S3G	Bae and Phillips, 2004
Aminotransferase	Transferase	*Psychrobacter* sp.	4RKC, 4RKD	Bujacz et al., 2015
Aspartate carbamoyltransferase	Transferase	*Moritella profunda*	2BE7	De Vos et al., 2007
Serine hydroxymethyltransferase	Transferase	*Psychromonas ingrahamii*	4P3M	Angelaccio et al., 2014

### Novel strategies for improving the expression of cold-active enzymes

**Figure 5:**
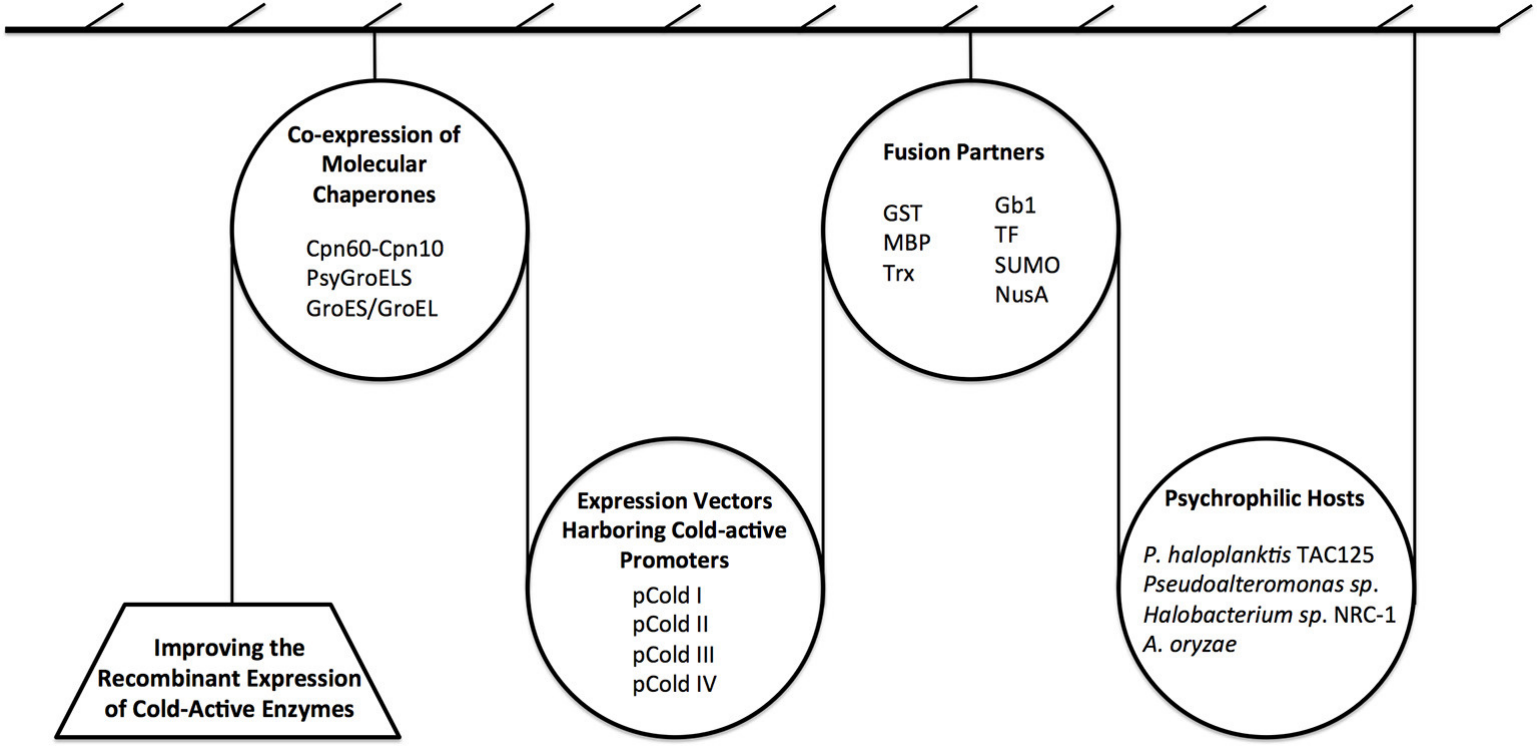
**Overview of novel strategies for improving the recombinant expression of cold-active enzymes**. Currently, the main approaches to produce enzymes at low temperatures include the use of: molecular chaperones, cold-active promoters, fusion partners, and psychrophilic hosts. A combination of the above strategies can also be used.

Several strategies have been suggested to promote proper expression and folding of cold-active enzymes expressed in heterologous host, increasing their solubility, activity, and yield. These strategies are summarized in Figure 5 and include the use of: (i) molecular chaperones; (ii) cold-active promoters; (iii) fusion partners; (iv) psychrophilic hosts, and (v) a combination of these strategies.

i) Molecular Chaperones

Molecular chaperones are ubiquitous proteins that help newly synthesized polypeptides and denatured proteins to reach their native conformation. They are widely distributed in bacteria, yeast, plants, and animals (Evstigneeva et al., 2001). Originally, they were discovered because their encoding genes were expressed under heat shock conditions, therefore this family of molecules was named heat shock proteins (HSP). However, genes encoding molecular chaperones are also induced under other stressful conditions including UV irradiation, hypoxia, and chemical challenges, among others (Whitley et al., 1999). There are various chaperones families, which are named by their molecular size ranging from HSP40 to HSP100 and the small HSPs (Hartl et al., 2011). In *E. coli*, chaperones with quaternary structure are also known as chaperonins and include the GroEL/GroEL systems, whereas monomeric chaperones include the DnaK/DnaJ systems (Bukau et al., 2000; de Marco, 2007).

In 2004, Ferrer and coworkers reported that expressing chaperones Cpn60 and Cpn10 from the psychrophilic bacterium *Oleispira antarctica* RB8 in *E. coli*, lowered its minimal growth temperature below 15°C (Ferrer et al., 2003, 2004a). They used this chaperone-*E. coli* system to express a heat-labile esterase, reporting for the first time a successfully expression system for heat-sensitive proteins. They demonstrated that the low temperature improved proper folding of the enzyme, enhancing its specific activity 180-fold in comparison to the enzyme purified from the usual *E. coli* strain grown at 37°C (Ferrer et al., 2004b). Today, a competent *E. coli* strain that co-express cold-active chaperones Cpn60 and Cpn10 is commercialized by Agilent Technologies under the name of ArcticExpress.

Kim et al. (2015) co-expressed a cold active esterase together with PsyGroELS, a chaperonin from the psychrophilic bacterium *Psychrobacter* sp. PAMC21119, in an *E. coli* strain. The expression was performed at 10°C and they compared the enzyme activity using the previously reported chaperones Cpn60 and Cpn10, finding better results with PsyGroELS for this particular enzyme. They conclude that PsyGroELS not only confers cold-tolerance to *E. coli*, but also is effective for co-expression of stable psychrophilic proteins.

Another example of chaperone co-expression was recently described by Esteban-Torres et al. (2014a) using GroES/GroEL chaperones. First, they cloned the cold-active esterase lp_2631 into the pURI3-TEV expression vector for protein production, but the recombinant protein was expressed as inclusion bodies when *E. coli* BL21 (DE3) was used as host. To solve this, they used the plasmid pGro7 that produces GroES/GroEL chaperones. When Lp_2631 was co-expressed with the molecular chaperones in the *E. coli* host, the protein was expressed in the soluble fraction of the cells.

ii) Cold-active promoters

Quing and coworkers developed cold-shock expression vectors (pColdI-IV) harboring the cspA promoter from CspA, the major cold shock protein of *E. coli*, allowing high expression of several genes upon induction by cold-shock (Qing et al., 2004). They reported that pCold vectors are highly complementary to the widely used pET vectors for the expression of 38 genes. pCold vectors have been used to functionally express various proteins in *E. coli* at low temperature, most of them from mesophilic organisms including human proteins that were difficult to obtain with other systems (Hayashi and Kojima, 2008). Surprisingly only a few examples have been described for the expression of enzymes from psychrophilic organisms. One of them corresponds to the pCold I vector, used to functionally express a cold-active β-galactosidase (rBglAp) that was found to be extremely heat-labile in *E. coli* (Nakagawa et al., 2007).

Shuo-shuo and coworkers cloned the cold-active lipase gene Lip-948, from the Antarctic psychrotrophic bacterium *Psychrobacter* sp. *G* into the plasmid pColdI and transformed it into *E. coli* BL21, obtaining substantive expression of lipase LIP-948 with a yield of 39% of total protein, most of which was present as inclusion bodies (Shuo-shuo et al., 2011). Co-expression of pColdI-Lip-948 with chaperone pTf16 and pGro7 decreased the amount of insoluble LIP-948, while the soluble expression was enhanced when pColdI-Lip-948 was co-expressed with “chaperone team” plasmids (pKJE7, pG-Tf2, pG-KJE8), respectively. LIP-948 was most efficiently expressed in soluble form when it was co-expressed with pG-KJE8, which was up to 19.8% of intracellular soluble proteins. Also, pCold vectors have been used for the expression of proteins with fusion partners, as it is described below.

iii) Fusion Partners

Fusion partners are solubility-enhancing tags used to increase both the solubility and expression level of recombinant enzyme expression (Hayashi and Kojima, 2010). They are located at the N- or C-terminus of the target protein and in some cases a specific cleavage site is placed between the tag and the target protein to allow their excision after purification. The most common fusion partners correspond to glutathione-*S*-transferase (GST), maltose-binding protein (MBP), thioredoxin (Trx), Gβ-1 domain of protein G (Gb1), nascent chain chaperone trigger factor (TF), small ubiquitin-like modifier (SUMO), and N-utilizing substance A (NusA).

Regarding expression of cold-adapted enzymes using fusion partners, Trx fusion tag has been used to obtain high yield of soluble psychrophilic yeast proteins in *E. coli* host (Illias et al., 2014). Moreover, the effects of seven different N-terminal fusion partners were studied to improve the solubility of proteins from the psychrophilic fish *Vibrio salmonicida* in *E. coli*. Among the fusion partners, MBP and NusA showed to be the best for expression yield and protein solubility (Niiranen et al., 2007). In addition, two different expression host strains and three cell culture incubation temperatures were used. Concerning the host strain, *E. coli* BL21-AI was shown to be superior to BL21(DE3)RIL CodonPlus for protein expression, but the product solubility was not affected by the choice of host. In terms of the incubation temperature for protein expression, the protein yield increased with temperature, although the effect on solubility was the contrary in most cases. They also concluded that small proteins were easier to express.

Another example for the soluble expression of a cold-active enzyme using MBP as the fusion partner was reported for a lipase from marine Antarctic origin (Parra et al., 2008). First, the expression system *E. coli* BL21(D3E)/pET22b(+) was used but the protein was obtained as inclusion bodies. After using the expression system *E. coli* TB1/pMAL-c2E, which expressed a fusion MBP-lipase protein, the enzyme was obtained in a soluble an active form. Hayashi and Kojima (2008) used the pCold I vector and modified it in order to express proteins fusioned to a GST tag. They were able to successfully express 9 proteins which they could not obtain using a conventional *E. coli* expression system. Later, the same authors used the pCold-GST system to successfully express 78 proteins from mesophilic organisms, showing that the primary sequence length of these proteins was not correlated with the expression level in the soluble fraction. They also developed three other cold-shock vectors using the fusion partners GB1, Trx, and MBP, showing that all systems were successful in obtaining soluble fusion proteins, with the pCold-GST system being the preferred and the pCold-MBP system the second choice. Furthermore, they reported that the use of a C-terminal 6 proline tag was successful in inhibiting the degradation of the protein during protein expression and purification, therefore being useful for enzyme stabilization (Hayashi and Kojima, 2010).

iv) Psychrophilic hosts

To overcome the decrease in protein yield and overall process productivity when *E. coli* strain is cultured at low temperatures, psychrophilic bacteria have been used as expression hosts (Parrilli et al., 2008b).

The most studied psychrophilic host is *Pseudoalteromonas haloplanktis* TAC125, which uses a modified *E. coli* cloning vector with psychrophilic molecular signals. This host was reported for the expression of a cold-adapted α-amylase as secretion carrier for extra-cellular protein targeting (Cusano et al., 2006a,b). Later, authors developed a *P. haloplanktis* TAC125 mutant strain that secreted a reduced number of exo-proteases, therefore reducing the extra-cellular proteolytic activity (Parrilli et al., 2008a).

In other study, an expression vector derived from psychrophilic bacterium *Pseudoalteromonas* sp. BSi20429 was constructed and *Pseudoalteromonas* sp. SM20429 was used as the psychrophilic bacterial strain. The system was first reported using a mesophilic promoter from *E. coli* and used for the active expression of a cold-adapted cellulase at 25–30°C (Zhao D. et al., 2011). Later, the mesophilic promoter was replaced by another from *Pseudoalteromonas* sp. BSi20429 that acted as a strong promoter at low temperatures and was also inducible by xylan, thus enabling the recombinant expression at lower temperatures. Multiple cloning sites and a His tag were also added to the expression vector, making these system useful for expressing *Pseudoalteromonas* enzymes that could not be maturely expressed in *E. coli* (Yu et al., 2015).

Another interesting study is an example to overcome the barrier of studying polyextremophilic enzymes. For halophilic enzymes, a high salt concentration is a requirement to obtain an active protein during overexpression in heterologous hosts. Karan et al. (2013) purified and characterized a halophilic and cold-active β-galactosidase from the cold-adapted haloarchaeon, *H. lacusprofundi*. They used the haloarchaeon, *Halobacterium* sp. NRC-1 strain as host in combination with a cold-shock protein gene promoter, *csp*D2, also from the host. They produced the recombinant β-galactosidase at 20-fold higher levels compared to *H. lacusprofundi*.

Finally, an example of a eukaryotic expression system for genes codifying cold-active enzymes comes from the work performed by Mao et al. (2015), who developed a novel uracil-deficient *Aspergillus oryzae* host for heterologous expression. This system was used to express an α-amylase from the psychrophilic fungus *Geomyces pannorum*.

v) Combination of the above strategies

Combination of these strategies has also been successfully used for the expression and purification of cold-adapted enzymes. A cold-adapted endo-1,4-β-glucanase from the earthworm *Eisenia fetida* was cloned in the pColdI vector and successfully expressed using the host strain ArcticExpress RT (DE3) (Ueda et al., 2014). Bjerga and Williamson (2015) optimized an expression system for cold-adapted proteins based on the pCold-II vector. They expressed five genes derived from metagenomic DNA from marine Arctic sediments and used three hosts strains including BL21 CodonPlus(DE3)RIL, ArcticExpress(DE3)RIL, and Rosetta2(DE3)pLysS, obtaining the best results using the latter. The yields of soluble protein were increased using fusion partners like MBP, TF, TRX, and SUMO, reporting the best results using large fusion partners like MBP and TF (Bjerga and Williamson, 2015).

## Evolutionary and molecular mechanisms of the cold-adaptation of enzymes

The ability of unicellular organisms to thrive in cold environments requires a vast array of adaptations in all levels, which enables to compensate for the perturbations stressed by these extreme environments. These adaptations cover from changes in the lipid composition of the cell membrane (Russell and Fukunaga, 1990) to sequence and structure changes in enzymes ensuring the efficiency of all biochemical reactions (Gerday et al., 2000).

The critical role of thermal adaptations on an enzyme's ability to remain highly active in the cold is easily understood if we consider that the metabolic and growth rates of psychrophilic and psychrotolerant species near the freezing point of water are higher than those of mesophilic organisms at the same temperature (Mohr and Krawiec, 1980; Knoblauch et al., 1999). To achieve this, enzyme function must be tuned in order to cope with the inherent temperature-dependent reduction of chemical rates and enable life in cold environments. Structurally, enzymes also require modification of their thermal stability and the dynamics of their three-dimensional structure in order to compensate for the freezing effects of low temperatures (Feller and Gerday, 2003), while at the same time avoiding catastrophic cold-induced unfolding events that impede proper function (Ramírez-Sarmiento et al., 2013). As thermal adaptations in enzymes are achieved by amino acid substitutions, insertions and deletions, the evolution and molecular basis of these adaptations in cold-adapted enzymes can be extracted mainly based on the comparison of their structural and functional features against mesophilic and thermophilic homologs. Here, we cover the main catalytic features of cold-adapted enzymes and the evolutionary and molecular mechanisms that allow these adaptations.

### Functional adaptations for high catalytic activity at low temperatures

The main mechanistic goal of the evolutionary adaptations in cold-active enzymes is to maintain a high catalytic activity at low temperatures. These activities are required to sustain metabolic activity in extremely cold environments, in some cases even near −20°C (Rivkina et al., 2000).

At very low temperatures the kinetic energy is insufficient to allow overcoming the kinetic barriers associated with an enzymatic reaction (Siddiqui and Cavicchioli, 2006). Nevertheless, cold adapted enzymes generally have optimum temperatures of activity and higher reaction rates at lower temperatures than their mesophilic homologs. We can rationalize this behavior if we examine the temperature dependence of the rate of chemical reactions as envisioned by the Arrhenius equation (Laidler, 1984):

(1) $$\begin{array}{l} k_{\text{cat}}=Ae^{{-E}_{a}/\left(RT\right)} \end{array}$$

Where *k*_cat_ is the catalytic rate, *E*_a_ is the activation energy of the reaction, *R* is the gas constant, *T* is temperature and *A* is a collision frequency factor. This equation illustrates how the catalytic rate depends on the temperature, such that it decreases upon decreasing temperature. For example, the catalytic rate of a mesophilic enzyme with *Ea* values ranging 50–75 kJ·mol^−1^ decreases 2–3-fold upon lowering the temperature every 10°C (Tattersall et al., 2012).

As suggested by the Arrhenius equation, the detrimental effect of lowering the temperature on the catalytic turnover can be countered by decreasing the activation energy, such that the thermal dependence of the catalytic reaction is reduced. Extensive reviews have been made about the changes in activation energies of chemical reactions catalyzed by psychrophilic enzymes and their mesophilic and thermophilic homologs, consistently showing that the activation energies are in fact decreased in cold-adapted enzymes (Lonhienne et al., 2001; Matsuura et al., 2002; D'Amico et al., 2002b, 2003a,b; Mavromatis et al., 2003; Garsoux et al., 2004; Liang et al., 2004; Fedøy et al., 2007; Leiros et al., 2007; Lian et al., 2015).

The temperature-dependence of reaction rates given by the Arrhenius equation explains the increase in *k*_cat_ needed for catalyzing reactions at low temperature under saturating substrate concentrations, whereas substrate binding represented by the Michaelis-Menten constant (*K*_m_) could also play an important role in increasing the catalytic efficiency (defined as *k*_cat_/*K*_m_) of cold-adapted enzymes at low temperatures (Feller and Gerday, 1997; D'Amico et al., 2002a). We can rationalize how these changes in the kinetic parameters are tuned within a cold-adapted enzyme through the transition state theory, in which equilibrium between the ground (ES) and a stable activated (ES^‡^) enzyme-substrate complex is assumed:

(2) $$\begin{array}{l} E+S⇌ES⇌{ES}^{‡}⇀E+P \end{array}$$

In this scenario, the temperature dependence of the catalytic rate can be related to the free energy of activation (ΔG^‡^) between the ground and activated that has to be overcome during an enzymatic reaction using the Eyring equation (Eyring, 1935):

(3) $$\begin{array}{l} k_{\text{cat}}=\frac{k_{\text{B}}T}{h}e^{\frac{{-△G}^{‡}}{RT}}=\frac{k_{\text{B}}T}{h}e^{\left(\frac{{△S}^{‡}}{R}-\frac{{△H}^{‡}}{RT}\right)} \end{array}$$

Where *k*_B_ is the Boltzmann constant, *h* is the Planck constant and Δ*H*^‡^ and Δ*S*^‡^ are the enthalpic and entropic contributions to the free energy of activation, respectively.

The comparative analysis of the kinetic parameters of a large number of psychrophilic enzymes has shown that their *k*_cat_ at low temperatures is similar to those observed for mesophilic enzymes at warm temperatures (D'Amico et al., 2002a; Siddiqui and Cavicchioli, 2006). In that scenario, either Δ*H*^‡^ must decrease or Δ*S*^‡^ must increase, as it is clear from Equation (3) (Lonhienne et al., 2000).

**Figure 6:**
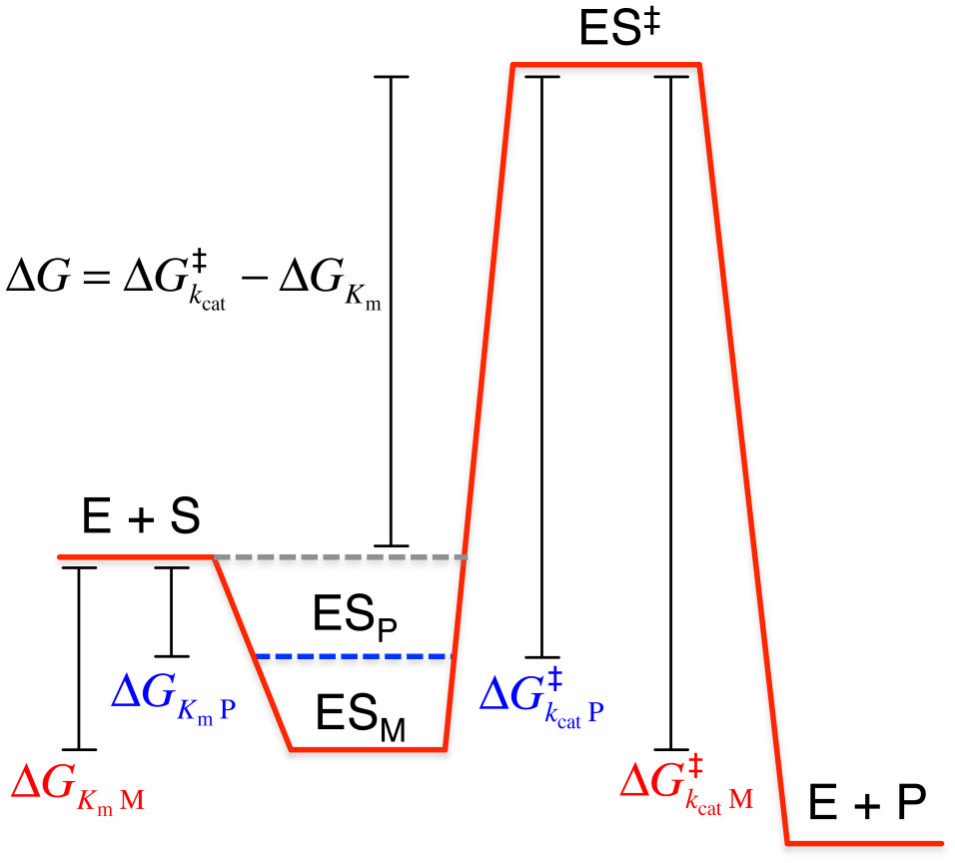
**Free energy changes between psychrophilic and mesophilic enzymes along the enzyme reaction coordinate from substrates (S) to products (P), according to the transition state theory**. The energy of the enzyme-substrate complex for the psychrophilic enzyme (ES_P_) is higher than for the mesophilic homolog (ES_M_), due to changes on the free energy of activation caused by decreasing the number of interactions broken to reach the transition state (enthalpic contribution) and increasing the protein flexibility (entropic compensation). These free energy changes lead to an increase in *k*_cat_ and a concomitant increase in *K*_m_. ^‡^, transition state.

The contribution of Δ*H*^‡^ can be understood in terms of the interactions that are broken while transitioning from the ground enzyme-substrate complex to the transition state of the reaction (Figure 6). Thus, a decrease of the enthalpic contribution translates into a reduction of the number of interactions that must be broken during this process (Siddiqui and Cavicchioli, 2006). This enthalpy decrease for psychrophilic enzymes is consistent with the decrease of the activation energy of the reactions catalyzed by these enzymes, as Δ*H*^‡^ = *E*_a_ − *RT* (Lonhienne et al., 2000).

Compensation of the change of enthalpic contributions for the formation of the transition state is achieved by an antagonist change in Δ*S*^‡^, which explains why *k*_cat_ does not increase exponentially upon changing Δ*H*^‡^ as a product of the cold adaptation of psychrophilic enzymes (Lonhienne et al., 2000). This compesation is such that the activation entropy difference between a mesophilic and a psychrophilic enzyme is always negative and the absolute value of their entropy difference, *T*Δ(Δ*S*^‡^), is always large (Lonhienne et al., 2000). This entropic compensation can be conceptualized in the context of the protein structure as an increase in flexibility of regions of the protein covering the enzyme's active site or other extensive changes in flexibility throughout the protein (Gerday et al., 1997). Extensive evidence from enzyme kinetics has shown that this is true for all cold-adapted enzymes studied so far (Siddiqui and Cavicchioli, 2006). Moreover, as a result of these changes in entropy and enthalpy, a small reduction of the free energy of activation and an increase of the conformational distribution of the ground state of the enzyme-substrate complex occurs (Figure 6). This trade-off between activity and stability is what leads to a small reduction of the free energy of activation in cold-adapted enzymes.

The enthalpic-entropic changes experienced by psychrophilic enzymes and represented in Figure 6 have two different consequences. First, increasing the flexibility of an enzyme through changes in plasticity of the active site leads to increased substrate promiscuity (Nobeli et al., 2009) because substrates with small variations in size and conformation can now fit into the more accesible binding site (Struvay and Feller, 2012; Feller, 2013) as it has been demonstrated for cold-adapted *Sporosarcina psychrophila* acylaminoacyl peptidase (Brunialti et al., 2011), *Shewanella gelidimarina* nitrate reductase, *Psychromonas ingrahamii* serine hydroxymethyltransferase (Angelaccio et al., 2012) and *Psychrobacter* sp. aminotransferase (Bujacz et al., 2015). This broader substrate utilization can be advantageous for protein engineering strategies focused in enhancing the specificity toward chemical reactions of biotechnological interest (Zhang et al., 2016). Second, these free energy changes, particularly the enthalpic changes, cause a decrease in substrate binding affinity. In this context, cold-active enzymes increase their *k*_cat_ at the expense of an increase in *K*_m_ (Feller and Gerday, 2003). In fact, stepwise single and multiple mutations engineered on a psychrophilic α-amylase to reconstruct the amino acid substitutions found in a mesophilic homolog exhibit a striking correlation of *k*_cat_ and *K*_m_, such that both decrease concomitanly upon increasing the number of mesophilic residues in the cold-adapted enzyme (Cipolla et al., 2011). Nevertheless, some enzymes from psychrophilic organisms that operate under subsaturating substrate concentrations within the cytoplasm exhibit a decrease in this kinetic parameter as an evolutionary strategy for cold adaptation (Bentahir et al., 2000; Hoyoux et al., 2001; Lonhienne et al., 2001).

In summary, cold-adapted enzymes generally exhibit an increase of their catalytic rate (*k*_cat_) allowed by a decrease in enthalpy due to a reduced number of protein-ligand interactions and an increase in entropy due to changes in their stability and flexibility, which can also lead to advantageous properties such as substrate promiscuity. In the following section we rationalize how these changes in stability and flexibility are embodied in the primary, secondary, tertiary (and sometimes quaternary) structure of these enzymes.

### Sequence and structure changes enabling high enzymatic activities at low temperatures

Thermophilic enzymes are known for having a higher thermostability than mesophilic enzymes and for being poor biocatalysts at room temperature (Gerday et al., 2000). Such thermostability, which is required to withstand heat denaturation at high temperatures, leads to increased conformational rigidity at temperatures where mesophilic enzymes usually catalyze their reactions (Závodszky et al., 1998). Interestingly, the conformational fluctuations are similar when comparing mesophilic and thermophilic enzymes at their respective optimal activity temperatures in which both *K*_m_ and *k*_cat_ are also optimal, the so-called “corresponding state” hypothesis (Závodszky et al., 1998). This evidence led to conclude that evolutionary adaptations, in the form of sequence and structure changes, allow a balance between protein stability and conformational flexibility that are responsible of proper function in the environmental niche's temperature of the source organism. In consistency with this idea, it has been argued that the plasticity or flexibility of cold-adapted enzymes is what enables their high specific activity at low temperatures and with a low energy cost (Gerday et al., 2000). It is now broadly accepted that the trade-off between thermostability and activity, and in particular the balance between stability and flexibility, is what evolves in enzymes in order to suit different environmental niches: for enzyme catalysis to be efficient at low temperatures protein flexibility must be increased, otherwise the reduced thermal fluctuations will diminish the conformational mobility and consequently compromise catalytic efficiency (Arnold et al., 2001). Moreover, it has been suggested that the encounter of cold-active enzymes with optimal activities at temperatures higher than their physiological conditions is evidence of an incomplete evolutionary adaptation to low temperatures (Georlette et al., 2004).

**Figure 7:**
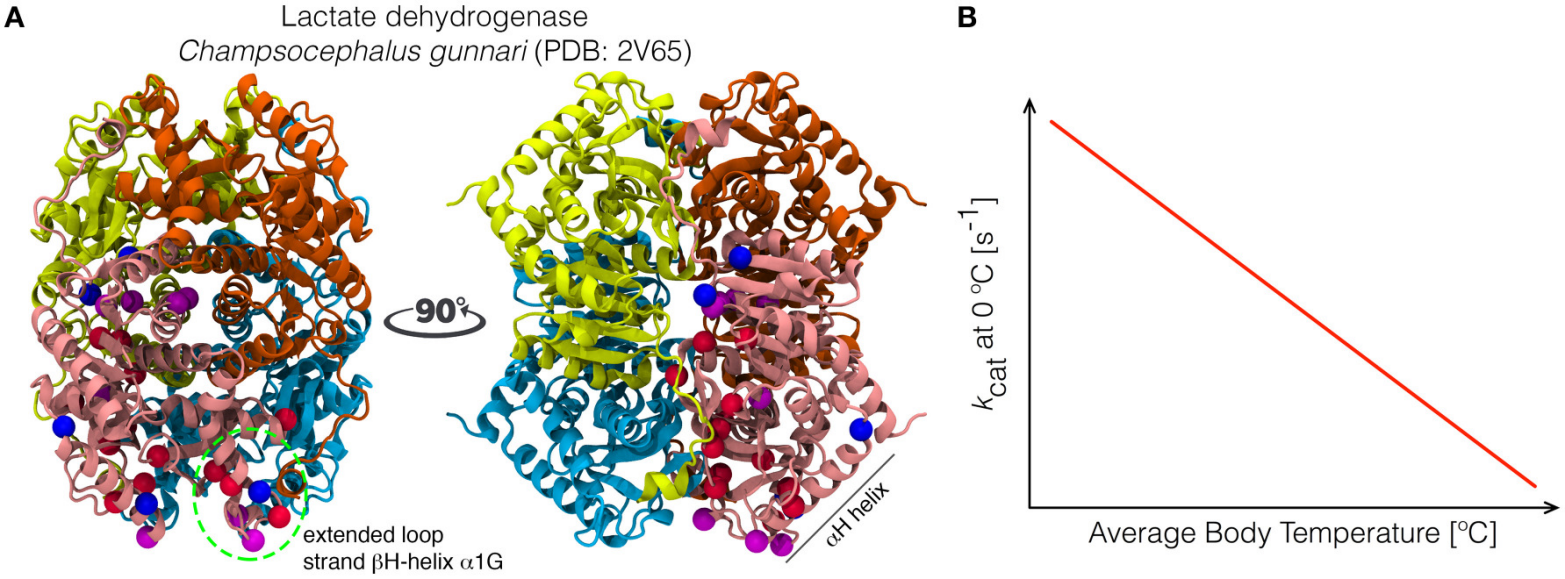
**Localized mutations are responsible for the temperature adaptations of lactate dehydrogenases in notothenioid fishes. (A)** Three-dimensional structure of the tetramer of lactate dehydrogenase from the Antarctic fish *C. gunnari*, showing the position of the mutations responsible for the changes between orthologs of these enzymes in thermal stability (measured as residual activity upon incubation at 50°C) and catalytic activity at low temperatures. The localization of these mutations compared to the consensus sequence are indicated as blue, magenta and red spheres for proteins with low, mild and high thermal stability, respectively. Most of them are located in structural elements (labeled in **A**) surrounding the active site. **(B)** The effect of mutations in the different positions indicated in A lead to changes in the catalytic rate of these enzymes in the cold, due to increased flexibility of regions neighboring the active-site, such that enzymes from notothenioids with lower body temperatures exhibit higher catalytic activities, as represented by the lineal regression shown in red (*y* = −4.6 × [s] + 231 [s^−1^]). Modified from Fields and Somero (1998).

Some of the first and most detailed evidences of this apparent increase in conformational flexibility came from the study of A4 lactate dehydrogenases (A4-LDH) from nine Antarctic and three South American notothenioid teleosts, which inhabited niches with temperatures ranging from −1.8 to 10°C (Fields and Somero, 1998). Enzyme activity assays revealed that the catalytic rate of A4-LDH from teleosts inhabiting the coldest environments were higher at 0°C than their homologs, with *k*_cat_ decreasing linearly as a function of average body temperature. More importantly, deduction of their amino acid sequences from RT-PCR and DNA sequencing showed that most of the minimal residue substitutions between A4-LDH that led to these catalytic differences were not distributed randomly, but located in two regions in the vicinity of the active site (helix αH and an extended loop connecting an helix with catalytic residues) whose conformational changes are rate-limiting steps for catalysis (Figure 7). Their results suggested that the observed substitutions increased the flexibility of these regions, leading to more rapid conformational changes and thus increasing *k*_cat_ (Fields and Somero, 1998).

Similar suggestions of the increased flexibility of cold-adapted enzymes were made based on gene cloning, protein purification and sequence analysis, combined with homology modeling of several enzymes generated using already crystallized mesophilic and thermophilic enzymes as templates (Russell, 2000). Such analysis led to the identification of interesting amino acid substitutions consistently found to occur in several cold-adapted enzymes when compared to their mesophilic and thermophilic homologs, such as the reduction of the number of surface salt bridges due to replacement of basic residues by glutamine or asparagine, changes in the distribution of surface charges, a reduced hydrophobicity of the protein core due to substitutions of bulky aromatic residues by more flexible nonpolar residues, a decrease in the number of hydrogen bonds in the protein structure, an increase in length of loop regions, among others (Davail et al., 1994; Feller et al., 1994; Smalås et al., 1994; Feller and Gerday, 1997; Russell, 2000). However, confirmation of these changes through the resolution of crystal structures of cold-adapted enzymes was lacking, mostly due to the difficulty of crystallizing these proteins (Russell, 2000).

The first crystal structures of cold-adapted enzymes were obtained for elastase (Berglund et al., 1995) and trypsin (Smalås et al., 1994) of Atlantic salmon, whereas the first solved crystal structures of bacterial psychrophilic enzymes corresponded to α-amylase (Aghajari et al., 1998), triose phosphate isomerase (Alvarez et al., 1998), and citrate synthase (Russell et al., 1998). Currently there are more than 50 different cold-adapted enzymes deposited in the Protein Data Bank (Berman et al., 2000), most of them coming from psychrophilic bacteria. The accession codes of most of these solved structures (excluding repeated structures of the same enzymes with ligands or mutations) are provided in Table 2, including a few remarkable examples of the use of metagenomic libraries to collect novel cold-adapted enzymes (Fu et al., 2013). It is worth noting that most of these enzymes correspond to hydrolases (Table 2), which comes as no surprise given that these are the most identified and studied cold-adapted enzymes for biotechnological and industrial applications, as we will see later on in this review.

Comparison of these deposited structures against mesophilic and thermophilic homologs confirmed that only minor structural modifications are needed to adapt warm-adapted enzymes to cold temperatures and that active-site residues involved in the reaction mechanisms are strictly conserved between homologous enzymes adapted to different temperatures (D'Amico et al., 2002a). Such comparisons allow determining the preferred amino acid exchanges and the localization of these changes within the protein structure, the variety of evolutionary strategies toward cold adaptation and also enable the reconstruction of the evolutionary steps that mediate temperature adaptations in the laboratory via rational design (Tsigos et al., 2001; Mavromatis et al., 2003).

In terms of changes in protein sequence, a systematic comparative analysis of multiple sequence and structure alignments containing 21 psychrophilic enzymes belonging to different structural families and 427 homologous mesophilic and thermophilic allowed to create a distance matrix of residue substitutions often found to allow adaptation to low temperatures: charged residues Arg and Glu tend to be replaced at exposed sites on α-helices by Lys and Ala, respectively; Val is replaced by Ala at buried regions in α-helices; and the content of Ala and Asn increases whereas Arg decreases in exposed sites (Gianese et al., 2001). Very similar results were recently obtained using archaeal genome analysis combined with high-throughput homology modeling (Saunders et al., 2003) and also using proteome-wide approaches on six completely sequenced species of psychrophilic and mesophilic bacteria (Metpally and Reddy, 2009). However, the location of these and other substitutions and the number of substitutions vary on a great extent depending on the enzyme under examination, meaning that each protein family adopts different structural strategies to adapt to low temperatures (Gianese et al., 2002).

**Figure 8:**
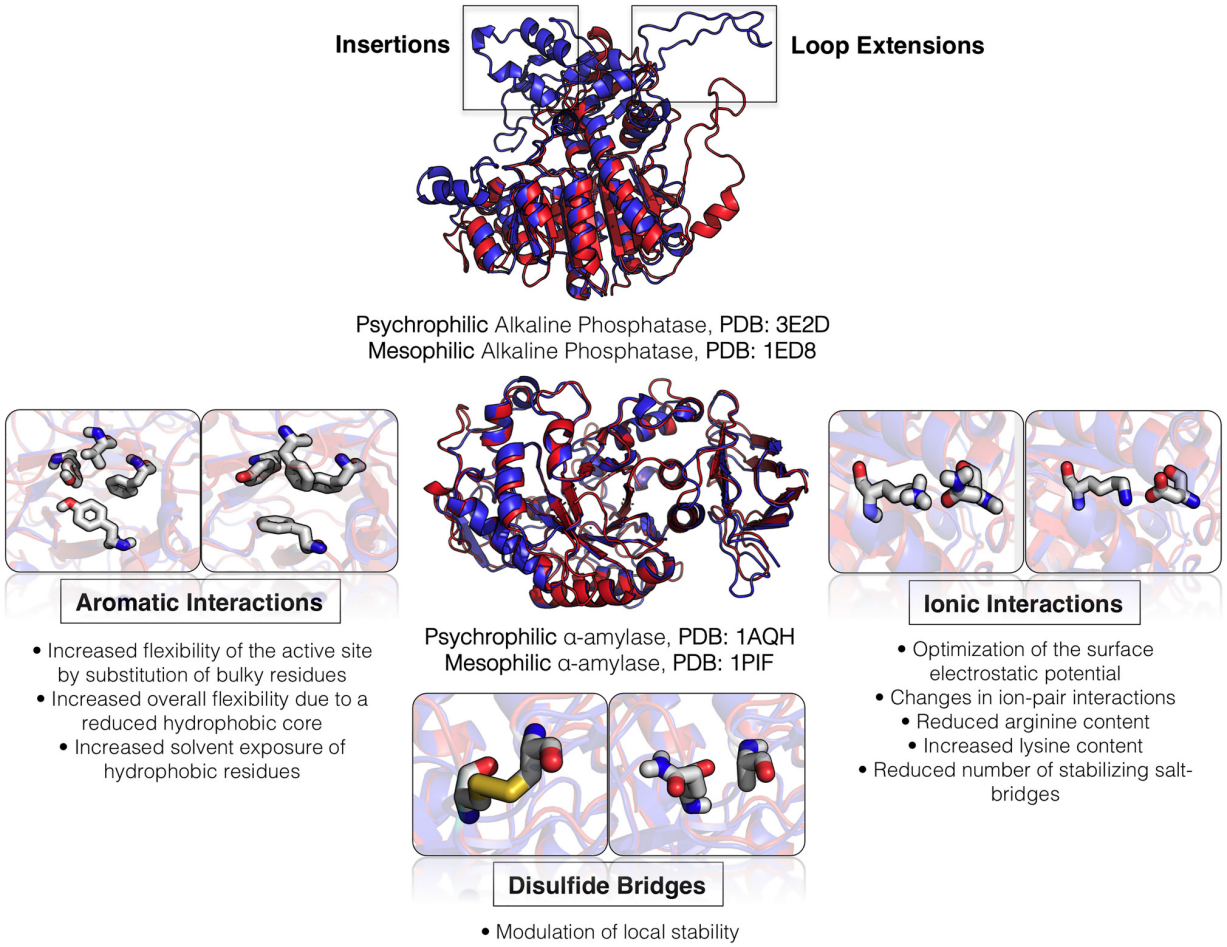
**Representative scheme of the most typical modifications in cold-adapted enzymes**. Psychrophilic and mesophilic alkaline phosphatases are compared to represent changes in the number of insertions and loop extensions, whereas psychrophilic and mesophilic α-amylases are used for visualizing changes in amino acid sequence related to the modification of several properties, listed below each type of amino acid changes. Modified from Helland et al. (2009) and Cipolla et al. (2011).

For example, in the case of *M. marina* triose phosphate isomerase, a single substitution of an alanine located within a loop that contacts the phosphate moiety of its substrate by a serine that is conserved in mesophilic enzymes is sufficient to increase the thermal stability and decrease the catalytic activity at low temperatures (Alvarez et al., 1998). The same is applicable in some cases for tuning mesophilic enzymes in order to sustain catalytic activities in the cold, as exemplified by the rationally designed single-point mutation I137M of *Bacillus subtilis* LipJ (Goomber et al., 2016b). Most frequently, evolutionary changes are related to multiple changes that lead to a more accessible and/or a more flexible active site due to substitution of bulky residues, insertions and deletions (Russell et al., 1998; Kim et al., 1999; Schrøder Leiros et al., 2000; Toyota et al., 2002; Aghajari et al., 2003; Van Petegem et al., 2003; Tsuruta et al., 2005, 2008; Leiros et al., 2007; Riise et al., 2007; Jung et al., 2008; Merlino et al., 2010; Jaremko et al., 2011; Malecki et al., 2013; Zheng et al., 2016), which in some cases are accompanied by the introduction of discrete amino acid substitutions in the active site that thermodynamically favor protein-ligand interactions at low temperatures, thus decreasing *K*_m_ (Lonhienne et al., 2001). Finally, the most extensive changes involve large portions throughout the protein structure and are related to optimization of the surface electrostatic potential to allow better interactions with the solvent and changes in ion-pair interactions (Bell et al., 2002; de Backer et al., 2002; Leiros et al., 2003; Bae and Phillips, 2004; Kumar and Nussinov, 2004; Arnórsdóttir et al., 2005; Helland et al., 2006; De Vos et al., 2007; Fedøy et al., 2007; Wang et al., 2007; Michaux et al., 2008; Pedersen et al., 2009; Alterio et al., 2010; Arimori et al., 2013; Bujacz et al., 2015), reduction of the number of hydrogen bonds (Matsuura et al., 2002; Bae and Phillips, 2004; Altermark et al., 2008; Michaux et al., 2008; De Santi et al., 2016), changes in loop extension, amino acid content, and flexibility (Bauvois et al., 2008; Helland et al., 2009; Zhang et al., 2011; Fu et al., 2013; Miao et al., 2016; Zheng et al., 2016), introduction or loss of disulfide bonds to modulate local stability (Violot et al., 2005; Helland et al., 2006; Wang et al., 2007), differential flexibility of domains in multidomain enzymes (Watanabe et al., 2005; Bauvois et al., 2008; Angelaccio et al., 2014), and enhanced protein solvation due to increased exposure of hydrophobic residues to the solvent (Aghajari et al., 1998; Russell et al., 1998; Maes et al., 1999; Bell et al., 2002; Van Petegem et al., 2003; Zhao Y. et al., 2012; Zheng et al., 2016). A summary of the most usual modifications responsible for cold-adaptation are shown in Figure 8. It is worth noting that not all of these mechanisms are required to explain the cold-adaptation of a given enzyme (De Maayer et al., 2014), although several proteins exhibit more than one of these mechanisms occurring in parallel (Coquelle et al., 2007), which suggest that comparative analysis within protein families might be better suited to solve the sequence-structure factors that explain the evolutionary adaptations of an enzyme of interest. Although it is rare to find proteins showing other mechanisms of cold adaptation, more extensive changes in protein topology (Tsuruta et al., 2005) or modifications of the oligomerization state that allows to increase the flexibility of solvent-exposed hydrophobic regions while simultaneously stabilizing the native fold of the enzyme (Skalova et al., 2005; Zanphorlin et al., 2016) have been also observed. However, these should be considered as evolutionary alternatives rather than as general mechanisms for enhanced flexibility in cold environments.

### Experimental and computational approaches to study the global and localized conformational flexibility of cold adapted enzymes

While solving the structures of these enzymes helped to deepen our understanding of the molecular mechanisms behind adaptation to cold temperatures, they only provide a static view of the position of these sequence changes within the three-dimensional space, thus lacking of an exploration of protein dynamics, with the only exception of those structures solved by NMR (Jaremko et al., 2011). Therefore, the combination of this structural information with experiments that assess the conformational flexibility of cold-adapted enzymes and the direct use of these structures as inputs for molecular dynamics is crucial to provide a solid framework for further experimental and computational protein engineering approaches.

Among the experiments performed to demonstrate the increased flexibility of cold-adapted enzymes, dynamic quenching of tryptophan fluorescence by increasing concentrations of acrylamide (Eftink and Ghiron, 1975) is commonly used. Acrylamide ascertains the accessibility of tryptophan residues within a protein as a decrease in fluorescence by means of physical contact (Eftink and Ghiron, 1976), thus reflecting the ability of the quencher to penetrate the protein structure and providing information of its permeability (D'Amico et al., 2003b). Typically, the fluorescence quenching constants (as reported by the Stern-Volmer constant) of psychrophilic enzymes are higher than for mesophilic proteins at both low and warm temperatures, thus indicating a more permeable structure (Huston et al., 2008; Tang et al., 2012), and the variation of fluorescence quenching (i.e., the change in the Stern-Volmer constant) within a temperature range where the native state prevails decreases in the order psychrophilic > mesophilic > thermophilic (D'Amico et al., 2003a; Georlette et al., 2003, 2004; Cipolla et al., 2012), thus indicating that cold-adapted enzymes possess higher flexibility. These experiments can also be combined with mutational analysis to explore the interplay between sequence variation, protein flexibility, and catalytic activity (Cipolla et al., 2011; Sigtryggsdóttir et al., 2014; Truongvan et al., 2016).

Further identification of the spots responsible for increased flexibility within a psychrophilic protein requires assessment of local regions of the protein. A successful approach for such task consists of the use of chimeric enzymes, in which a gene encoding for a given psychrophilic protein is divided into several regions that are then replaced by similar regions from a mesophilic homolog (Yoneta et al., 2004; Watanabe et al., 2005). This strategy allows not only to confirm that in some cases the entire protein does not necessarily need to be flexible to achieve high catalytic activity in at low temperatures, but also to identify which protein regions are responsible for the increased flexibility (Yoneta et al., 2004), which can be further combined with mutational analysis to identify the key residues responsible for cold-adaptation (Hayashi et al., 2014). These experiments allowed the identification of the C-terminal region of the cold-adapted isocitrate dehydronease from *Colwellia maris* as responsible for its psychrophilic characteristics (Yoneta et al., 2004). Another powerful strategy corresponds to amide hydrogen/deuterium exchange mass spectrometry (Balasubramaniam and Komives, 2013), in which the exchange between backbone amide protons and the deuterium from the surrounding solvent is used as a mass probe for the solvent accessibility of a protein, whereas quenching and pepsin digestion of the protein followed by mass spectrometry analysis of the resulting peptides allows to localize the sites of exchange within the protein. In these experiments, highly flexible regions become fully deuterated in a few minutes, whereas well-packed regions such as the hydrophobic core exhibit a low extent of exchange. The advantages of this strategy is that it can be applied to proteins of any size (Balasubramaniam and Komives, 2013), under varying temperature (Ramírez-Sarmiento et al., 2013), and solvent conditions (Medina et al., 2016) and in the absence and presence of ligands (Chalmers et al., 2011). Comparative analysis of deuterium incorporations of local regions of a psychrophilic and a thermophilic alcohol dehydrogenase led to strengthen the notion that only those functional regions related to substrate binding exhibit greater flexibility in the cold-active enzyme than in the warm-adapted homolog, suggesting that local flexibility can be uncoupled from thermal stability (Liang et al., 2004).

The use of solved or homology modeled psychrophilic protein structures in molecular dynamics allows the assessment of dynamical features relevant for cold adaptation with atomistic resolution. Constant temperature simulations of psychrophilic enzymes at several temperatures within the range 10–45°C enabled the identification of loops near active sites that exhibit higher flexibility in comparison with their mesophilic homologs, as in the case of Uracil-DNA glycosylases (Olufsen et al., 2005), elastases (Papaleo et al., 2006), and β-glucosidases (Zanphorlin et al., 2016), and the optimization of ion-pair networks near the active sites of elastases (Papaleo et al., 2007) and serine proteases (Tiberti and Papaleo, 2011). In some cases, these simulations show good correlation between the optimal temperature of catalytic activity and the increased flexibility of functional regions of the protein (Aurilia et al., 2009) and also highlight other loops distant from the active site that exhibit preservation of similar flexibilities between psychro-, meso-, and thermophilic enzymes at their optimal temperature for catalysis (Kovacic et al., 2016). Other quasi-harmonic entropy approximations have been used for comparative analysis of simulations of psychrophilic enzymes in their free and substrate-bound forms, allowing the identification of key determinants of structural flexibility at the residue-level (Kosugi and Hayashi, 2011). More recently, complex molecular simulation strategies have allowed bridging the reduction of the activation energies of enzymatic reactions with the increased flexibility of cold-adapted enzymes. Hybrid quantum-mechanics/molecular-mechanics simulations on a psychrophilic α-amylase from *Pseudoalteromonas haloplanctis* revealed that formation of the transition state of the enzymatic reaction is accompanied by a rearrangement of a loop neighboring the active site, such that it interacts with the substrate via water-mediated and direct interactions, and is crucial for the reduction of the free energy barrier of the hydrolysis reaction (Kosugi and Hayashi, 2012). Moreover, energetic estimations of peptide hydrolysis by psychrophilic trypsins calculated using free energy perturbation simulations in which the flexibility of the protein surface is systematically reduced through position restraints of different strengths, showed that this protein rigidity is sufficient to increase the activation energy as in mesophilic enzymes, thus strongly suggesting that softness of the protein-water surface is what tunes the temperature adaptation of catalytic rates (Isaksen et al., 2016).

The molecular mechanisms of cold-adaptation and the hotspots of conformational flexibility captured through the application of these experimental and computational approaches are not only compelling evolutionary and theoretical challenges to pursue, but also provide paramount information to integrate in protein engineering and design endeavors. As we will see below, a vast number of rational design and directed evolution approaches used to improve catalysis at low temperatures are proposed based on localized conformational flexibility spots revealed by these types of analysis.

## Protein engineering of cold-active enzymes

Protein engineering has emerged as a strategy to optimize a specific property of an enzyme *in vitro*, such as their thermal stability, substrate specificity and activity at extreme temperatures. This is performed through the introduction of mutations into a protein sequence in order to allow “evolution” toward a target feature. Rational design and directed evolution are the two most general approaches to attempt protein engineering. Rational design is based on site-specific mutagenesis, therefore the structure, function, and catalytic mechanisms of the protein must be known (Arnold, 2001; Tang and Zhao, 2009; Bornscheuer et al., 2012; Reetz, 2013). When no detailed structural information of the enzyme is available, the typically applied strategies are error-prone PCR (epPCR) (Leung et al., 1989) and DNA shuffling (Stemmer, 1994). An actual trend of directed evolution is the creation of “smarter,” high-quality libraries, with a reduced library size and fast in reaching beneficial mutations (Kazlauskas and Lutz, 2009; Bornscheuer et al., 2012; Kille et al., 2013; Parra et al., 2013; Wijma et al., 2013).

As we have largely stressed in this review, the temperature adaptability of the catalytic properties exhibited by enzymes obtained from organisms adapted to extreme environments, makes them interesting biocatalysts for biotechnological and commercial applications. However, further improvements to the activity, substrate specificity, or stability of cold-adapted enzymes are often needed to better suit specific industrial applications. In this regard, the elucidation of the molecular mechanisms and the trade-off between thermostability and activity underpinning the cold-adaptation of enzymes have been crucial for the application of protein engineering strategies that either enhance some of the properties of cold-adapted enzymes or modify meso- and thermophilic enzymes to be able to catalyze reactions at low temperatures. Here, we discuss some examples of successful applications of several protein-engineering approaches for achieving these goals.

Rational design has been used to improve the thermal stability and activity of cold-adapted citrate synthases by introduction of residue substitutions and loop insertions that reduce the accessibility of the active site in hyperthermophilic homologs, leading to an enzyme with increased thermal stability and lower optimal temperatures of activity (Gerike et al., 2001). Other rational designs focused on increasing the flexibility due to the introduction of more flexible residues, such as the single-point mutation I137M in the mesophilic *Bacillus subtilis* lipase LipJ, which led to a 17°C downshift of the optimal temperature of activity and to cold adaptation (Goomber et al., 2016b). A triple mutant of a psychrophilic alkaline phosphatase in which the bulky aromatic residues on the substrate binding sites were replaced by more flexible amino acids, led to an enzyme with increased stability that retains the psychrophilic character of the wild-type enzyme (Tsigos et al., 2001). Other attempts of increasing the rigidity of the active site of these enzymes through engineering of disulfide bonds have led to more stable proteins but accompanied by a large reduction of their catalytic rates (Ásgeirsson et al., 2007). Site-directed mutagenesis of cold-adapted endo-1,5-α-L-arabinanase has also allowed to shift the optimum pH of activity toward acidic conditions for their use in pectin extraction and juice clarification (Wang S. et al., 2014). Finally, computational analysis of residue packing and atomic displacement parameters in structures of cold-active lipases allowed identification of highly flexible regions within a protein, whose residues were experimentally manipulated via rational design and site saturation mutagenesis to obtain variants with seven-fold increased thermal stability without loss of their cold-adapted properties (Cesarini et al., 2012).

Site saturation mutagenesis has been used to identify substitutions that affect enzyme activity and are not easily predicted by rational approaches. Site saturation mutagenesis on a cold-active β-galactosidase, which was unexpectedly inactivated by a rationally designed mutation, led to the identification of a double mutation within the active site that increased the catalytic activity in 2.5-fold and showed faster hydrolysis of skim milk's lactose at low temperature than the wild-type enzyme (Coker and Brenchley, 2006). The same strategy of saturation was exhaustively applied onto all the 88 loop residues of a mesophilic lipase from *Bacillus subtilis*, finding 5 substitutions within loops around the enzyme's active site that increased their conformational flexibility and, when combined into a 5-residue mutant, led to a lipase with a seven-fold catalytic activity enhancement at 10°C and increased catalytic activity within the range 5–60°C when compared to the wild-type enzyme (Kumar et al., 2014).

The most successful strategy for engineering novel cold-adapted enzymes and optimizing the properties of enzymes extracted from organisms inhabiting cold environments has been the use of directed evolution. Results from this strategy usually illustrate that cold adaptation of enzymes can be achieved through multiple routes. One of the first examples corresponds to random chemically-induced mutagenesis and low-temperature activity screening assays on mesophilic alkaline serine protease subtilisin, which led to obtain two different triple mutants, whose substitutions were located in different regions of the protein, with each one leading to improved catalytic activity at 10°C due to either a decrease in *K*_m_ (Taguchi et al., 1998) or an increase in *k*_cat_ (Taguchi et al., 1999). A similar strategy based on error-prone PCR was applied on psychrophilic lipases from *C. antarctica* (Zhang et al., 2003) and *Pseudomonas fragi* (Gatti-Lafranconi et al., 2008), leading to enzyme with increased half-life times against thermal inactivation, and on a metagenomically isolated mesophilic *Bacillus* lipase, generating a single mutation that conferred optimal activity at 10°C due to increased localized flexibility and reduced thermal stability (Goomber et al., 2016a). Combination of directed evolution with subsequent rounds of rationally designed site-directed mutagenesis, led to 6 substitutions within structured and unstructured regions near the active site of a thermophilic subtilase, which were not related to the substitutions found in naturally occurring cold-adapted homologs but enhanced casein hydrolysis at low temperatures, due to a downshift both in the thermal stability and the optimal catalytic temperature (Zhong et al., 2009). The same strategy was applied onto a xylanase from *Paenibacillus campinasensis* to improve its resistance to high alkaline and temperature conditions for their potential use in pulp and paper industry (Zheng et al., 2014).

A more coarse approximation consists of DNA shuffling for either generating chimeric enzymes of psychrophilic and warm-adapted homologs or allowing combinatorial extension of variants generated by mutagenesis. Transferring a highly flexible 12-residue region of a psychrophilic subtilisin into a mesophilic homolog from *Bacillus lentus* generated a chimeric enzyme with cold adaptation characteristics (Tindbaek et al., 2004). Also, the combination of directed evolution with DNA shuffling on a glycine oxidase from *Bacillus licheniformis* led to engineering a cold-adapted enzyme with increased catalytic activity against the herbicide glyphosate, which can be potentially used to confer resistance on genetically modified crops (Zhang et al., 2016).

It is worth noting that, although most of the attempts for unleashing the full biotechnological potential of cold-adapted enzymes as biocatalysts rely on protein engineering strategies, the application of chemical modification strategies, such as protein immobilization, have been also successful in improving the stability of cold-adapted enzymes for industrial processes and also to enable the removal and recovery of these enzymes for continuous use. One of these few examples corresponds to the immobilization of a cold-adapted pullulanase “extremozyme” from *Exiguobacterium* sp. on epoxy-functionalized silica particles, which significantly improved the thermal stability after hour-long incubations at 60 and 70°C in comparison to the fast inactivation of the free enzyme after 5 min incubation at the same temperatures, thus constituting a potential candidate for starch hydrolysis at low temperatures (Rajaei et al., 2015).

## Potential biotechnological applications of the reviewed enzymes

The use of cold-adapted enzymes in chemical processes not only allows energy saving, but also performing chemical reactions at low temperatures in order to avoid chemical side-reactions that can occur at higher temperatures (Siddiqui, 2015). Moreover, the rapid inactivation of cold-active enzymes at moderate temperature because of their heat-lability is a good option for food industry, fine-chemical synthesis, and molecular biology applications (Cavicchioli et al., 2011). The biotechnological potential of cold adapted enzymes is very broad and have been extensively reviewed (Gerday et al., 2000; Cavicchioli et al., 2002, 2011; Gomes and Steiner, 2004; Marx et al., 2004, 2007; Margesin et al., 2007; Huston, 2008; Margesin and Feller, 2010; Nevalainen et al., 2012; Feller, 2013; Elleuche et al., 2014; Sarmiento et al., 2015; Siddiqui, 2015). Specific examples of biocatalysis using cold-active enzymes isolated from bacteria (Morita et al., 1997; Russell, 1998), yeast (Buzzini et al., 2012; Alcaíno et al., 2015), and fungi (Nevalainen et al., 2012) have also been documented, as well as specific reviews of the biotechnological potential of pectinases for food industry (Adapa et al., 2014), lipases (Joseph et al., 2008; López-López et al., 2014; Maiangwa et al., 2015), and xylanases (Collins et al., 2005; Dornez et al., 2011). Here, we summarize the biotechnological potential of some of the enzymes from Table 1.

### Testing cold-active enzymes under additives or industrial-like conditions

Enzymes used in chemical processes need to be active in the presence of other additives required for these reactions. Organic solvents are widely used, either pure or mixed with aqueous solvents. In general, to obtain some information about cold-active enzymes after purification, residual enzymatic activity was studied in the presence of different additives, such as metal ions, EDTA, DTT, β-mercaptoethanol, and protease inhibitors. In addition, enzyme stability upon addition of organic solvents and salt was also studied for some cold-active enzymes. The effect of different additives was assessed for 76% of the cold-active enzymes examined in Table 1.

In reaction mixtures, organic solvents are used to increase the solubility of hydrophobic substrates, as in biodiesel production through transesterification reactions using lipases and esterases. However, enzyme activity is reduced in organic solvents because water molecules are lost (Doukyu and Ogino, 2010). At low temperatures, cold-active enzymes are able to grasp more tightly to available water molecules because they have a low inherent surface hydrophobicity (Karan et al., 2012). For this reason, cold-active enzymes preserve their catalytic activity in organic solvents because they are able to maintain a tight hydration shell. One example of a cold-active esterase that is active under high concentrations of different solvents and additives is the esterase LipA from *Sorangium cellulosum* (Cheng et al., 2011). The enzyme retained high level of activity in the presence of 0.1-1% of the commercially available detergents (Tween 20, Tween 80, Triton X-100). Also, the activity was tested after incubation with 16 different solvents. Diethylether, chloroform, benzene, toluene, *p*-xylene, cyclohexane, *n*-hexane, *n*-heptane, and isooctane increase LipA activity and the others have minor negatives effects, conserving always more than 50% activity. Another study of a cold-active enzyme stable in organic solvent was reported for lipase AT2 from *S. epidermidis* (Kamarudin et al., 2014). This cold-active enzyme was found stable in both hydrophilic and hydrophobic organic solvents. The enzyme displayed stability not only in methanol, ethanol and acetone but the lipolytic activity was also enhanced in the presence of DMSO and diethyl ether. In addition, the enzyme was catalytically active in toluene and n-hexane mixture, which is the preferred solvent in most of the transesterification reactions. One last example of lipases/esterases active in non-aqueous solvent systems is the lipase ReLipA from *R. endophyticus* (Yan et al., 2016) which exhibited excellent ability to catalyze the synthesis of methyl oleate, ethyl oleate, and butyl oleate in isooctane solvent system with a maximum yield of 82.2%. In addition, the enzyme is stable in different organic solvents.

Since an important number of cold-active enzymes are isolated from marine environments, some of them are also halophiles. One example is a salt-tolerant esterase, Est12 from *Psychrobacter celer*, which catalyze reactions and degrade organic matters under high salt concentrations (Wu et al., 2013a,b). Est12 was isolated from deep-sea sediments and showed enhanced activity and stability in 4.5 M NaCl, with *Km* decreasing from 0.069 to 0.033 mM *p*-NB and *kcat* doubled to around 9.21 s^−1^ compared to the enzyme without salt. Moreover 0.5 and 1% (v/v) non-ionic detergents (Tween 20, Tween 80, Triton 100 and CHAPS) significantly enhanced the activity, in some cases up to 200%. After incubation with 5-30% (v/v) ethanediol, methanol, DMSO as well as 5-20% isopropanol and ethanol for 1 h, Est12 retained more than half of its activity.

More in depth, based on the remarkable activity of Pul-SH3 in the presence of SDS, two commercial detergents, Rika (7.5% v/v) and Fadisheh (2.5% w/v), were used to assess the potential application of the enzyme for washing purposes. The results showed that the enzyme was highly active in the presence of these detergents by 80.4 and 93.7%, respectively. In addition, the stability of the enzyme against the commercial detergents was interestingly high, so that the remaining activity after a 10-day holding at room temperature with Rika (7.5% v/v) and Fadisheh (2.5% w/v) was about 54.5 and 85%, respectively (Rajaei et al., 2015).

From the cold-active enzymes reviewed in Table 1, most of them claim potential uses in industrial processes. However, only a few went a step further and made at least a small trial in a real application, commonly for the food industry. These examples include three β-galactosidases for the hydrolysis of lactose in milk, one methylesterase for fruit firming, one polygalacturonase for juice industry, and a glycogen branching enzyme with biotechnological potential in bread production.

The first example was previously mentioned in this review and reported by Dong and coworkers (Dong et al., 2014) and corresponds to a cold-active but thermostable β-galactosidase. The enzyme was expressed as both a soluble protein and in the form of inclusion bodies. The active inclusion bodies of β-galactosidase were easily isolated by nonionic detergent treatment and directly used for lactose conversion in a repetitive batch mode. The enzyme lost ~5% (90°C) or 1% (10°C) activity after each reaction cycle. More than 54% (90°C) or 88% (10°C) of the original enzyme activity was retained after 10 conversion cycles under optimum conditions. These results suggest that the recombinant thermostable β-galactosidase may be suitable for the hydrolysis of lactose in milk processing, with the advantages of being active at low temperatures and cost-convenient. The second example is the attractive activity of *Lactococcus lactis* β-galactosidase at low temperatures, for which its efficiency as biocatalyst to bioconvert lactose within milk during storage was explored. For this purpose, they performed lactose hydrolysis in milk at 4 and 10°C. Using the free enzyme or immobilized cells, bioconversion rates of nearly 98% were achieved after 7 and 6 h of incubation, respectively. The immobilized cells were recycled and used several times, followed by enzyme activity measurements. Using immobilized *E. coli* NovaBlue cells expressing the β-galactosidase, more of the 96% of the initial activity was retained after 10 cycles of use at 4°C (Vincent et al., 2013). One last example of a β-galactosidase with potential applications in milk and dairy product industry is the enzyme from *Antarctic Arthrobacter* sp. 32cB, which has the capacity to hydrolyzed 90% of the lactose in 1 mL of milk at 10°C in 24 h (Pawlak-Szukalska et al., 2014).

Another example of enzymes with potential applications in food industry is an acidic and cold-active pectin methylesterase PE8F46 that was identified from *P. chrysogenum* and successfully expressed in *P. pastoris* (Pan et al., 2014). This enzyme was shown to significantly improve the firmness of pineapple dices in combination with calcium lactate, compared with a commercial pectinase complex. Thus, it represents an excellent candidate for food processing in the fruit and vegetable industry, considering the requirement of low-temperature to keep fruit quality.

An example concerning the juice industry is polygalacturonase, Endo-PG I, which was shown to reduce the viscosity of papaya juice by 17.6%, and increased its transmittance by 59.1% (Tu et al., 2013). When combined with a commercial pectin methylesterase, it showed higher efficiency with a synergy degree of more than 1.25. Currently, the widely used polygalacturonase has a pH optimum of 3.5, which is lower than the papaya juice pH (5.7). Endo-PG I have a slightly acid pH optimum (6.0), is cold active and stable in a large range of temperatures, properties required for potential applications in the juice industry.

The last example of application in food industry is a glycogen branching enzyme (RmGBE) from the thermophilic fungus *Rhizomucor miehei* that showed interesting cold-adapted characteristics (Wu et al., 2014). Addition of RmGBE to wheat bread resulted in a 26% increase in specific volume and a 38% decrease in crumb firmness in comparison with the control. Besides, the retrogradation, determined by measuring the crumb firmness and chewiness of bread, was significantly retarded along with the enzyme reaction. These properties make RmGBE highly useful in the food and starch industries.

Two nice examples of cold-active enzymes with potential uses in biomedicine were also reported for a α-galactosidase and a nitroreductase. The possibility to generate a universal blood type from B-type blood for application in transfusion therapy has been studied using enzymes. Some α-galactosidases are capable of removing the antigenic component from surface carbohydrates of group B red blood cells. One example is the cold-active α-galactosidase from *Pseudoalteromonas* sp. strain KMM 701 that showed to convert B red blood cells into blood type O cells at neutral pH (Balabanova et al., 2010). The activity of the enzyme was first observed when it was purified from its natural host producer. In view of its application, this cold-active enzyme was then overproduced in a heterologous host (Bakunina et al., 2014). An example of a potential cold-active enzyme for prodrug therapy was described using a cold-active nitroreductase, Ssap-NtrB (Çelik and Yetis, 2012). Despite Ssap-NtrB derived from a mesophilic bacterium, it showed optimal activity at 20°C against cancer prodrugs. Authors comment that the cold-activity of this novel enzyme will be useful for therapies in combination with crymotherapy, exposing the target tissue to low temperatures in order to trigger the enzyme activity to activate the drug only where is required. Moreover, the enzyme could also be used for bioremediation of compounds of explosive and volatile nature in regions where high activity at low temperatures is needed.

## Conclusions and perspectives

In this article, we have reviewed cold-active enzymes discovered between 2010 and June 2016 from culture-dependent bioprospecting and also some few enzymes discovered by genome mining of psychrophilic microorganisms, aspects that have not been reviewed elsewhere. Interestingly, these cold-active enzymes were isolated not only from microorganisms living in cold environments, but also from mesophilic and even thermophilic microbes. By far, hydrolases were the most frequent class of enzymes isolated, probably because of the vast potential applications that this type of cold-active enzymes might have, due to their significant activities in diverse reactions and their potential catalysis of novel hydrolytic transformations (López-Iglesias and Gotor-Fernández, 2015). Lipases and esterases covered together 42% of the hydrolases from Table 1, which is consistent with the worldwide use of lipases due to their features, as they are easy to handle, active in non-aqueous medium and are able to catalyze chemo-, regio-, and enantio-selective transformations (Kumar et al., 2016). The second largest class was oxidoreductases, but only with four representatives compared to the 84 hydrolases. A similar trend was observed for the representation of different types of cold-adapted enzymes for which their structures have been solved, as shown in Table 2. Therefore, there is a big opportunity for the isolation of novel cold-active enzymes from members of other classes, which have been less explored. The majority of the enzymes were isolated from microorganisms living in diverse places of the Polar Regions and oceans, and most of these microorganisms were bacteria. For enzyme production, the most used host was *E. coli* (85%) followed by *P. pastori* (10%). Concerning expression vectors, the common pET vectors were the choice. Is surprising that despite the existence of special designed hosts and expression vectors for the recombinant production of cold-active enzymes, still classic *E. coli*/pET systems are preferable. We argue that more studies comparing different expression systems for cold-active enzymes are needed, in order to give more evidences of the advantages of using other hosts and expression plasmids. Indeed, we have also addressed the progress made in the overexpression and purification of cold-adapted enzymes, giving examples of enzymes that were only obtained soluble when using special expression systems and fusion partners.

We have also covered the evolutionary and molecular origins of the temperature adaptations exhibited by these enzymes, as well as diverse computational and experimental techniques to ascertain these adaptations. The value of understanding the molecular mechanism of these adaptations comes from their potential use in protein engineering strategies, some of which we also covered in this review. While the most used technique for protein engineering corresponds to directed evolution and the most straightforward example of the use of these insights were rational design strategies, the identification of rigid and flexible regions within proteins allows establishment of potential hotspots for the modification of the structural properties of these localized regions by site saturation mutagenesis.

Given the extensive literature on the applications of cold-active enzymes in biocatalysis, we only cover specific examples of potential applications given for enzymes reviewed in Table 1. However, only a few of the characterized enzymes were studied for a real industrial application and most of them in the food industry. It will be interesting to see more original articles covering other examples of a concrete use of these remarkable enzymes in the future, which are known to be very relevant for various industrial processes and whose applications will be potentially widespread in the following years.

## Author contributions

All authors listed, have made substantial, direct and intellectual contribution to the work, and approved it for publication.

## Funding

Pontificia Universidad Católica de Chile.

### Conflict of interest statement

The authors declare that the research was conducted in the absence of any commercial or financial relationships that could be construed as a potential conflict of interest.

## References

[B1] Acevedo J. P. Rodriguez V. Saavedra M. Muñoz M. Salazar O. Asenjo J. A.. (2013). Cloning, expression and decoding of the cold adaptation of a new widely represented thermolabile subtilisin-like protease. J. Appl. Microbiol. 114, 352–363. 10.1111/jam.12033 23043619

[B2] Adapa V. Ramya L. Pulicherla K. Rao K. S. (2014). Cold active pectinases: advancing the food industry to the next generation. Appl. Biochem. Biotechnol. 172, 2324–2337. 10.1007/s12010-013-0685-1 24390855

[B3] Aghajari N. Feller G. Gerday C. Haser R. (1998). Structures of the psychrophilic *Alteromonas haloplanctis* α-amylase give insights into cold adaptation at a molecular level. Structure 6, 1503–1516. 10.1016/S0969-2126(98)00149-X 9862804

[B4] Aghajari N. Van Petegem F. Villeret V. Chessa J. Gerday C. Haser R.. (2003). Crystal structures of a psychrophilic metalloprotease reveal new insights into catalysis by cold−adapted proteases. Proteins 50, 636–647. 10.1002/prot.10264 12577270

[B5] Albino A. Marco S. Di Maro A. Chambery A. Masullo M. De Vendittis E. (2012). Characterization of a cold-adapted glutathione synthetase from the psychrophile *Pseudoalteromonas haloplanktis*. Mol. Biosyst. 8, 2405–2414. 10.1039/c2mb25116g 22777241

[B6] Alcaíno J. Cifuentes V. Baeza M. (2015). Physiological adaptations of yeasts living in cold environments and their potential applications. World J. Microbiol. Biotechnol. 31, 1467–1473. 10.1007/s11274-015-1900-8 26160010

[B7] Almog O. González A. Godin N. de Leeuw M. Mekel M. J. Klein D.. (2009). The crystal structures of the psychrophilic subtilisin S41 and the mesophilic subtilisin Sph reveal the same calcium−loaded state. Proteins 74, 489–496. 10.1002/prot.22175 18655058

[B8] Alterio V. Aurilia V. Romanelli A. Parracino A. Saviano M. D'Auria S.. (2010). Crystal structure of an S−formylglutathione hydrolase from *Pseudoalteromonas haloplanktis* TAC1251. Biopolymers 93, 669–677. 10.1002/bip.21420 20209484

[B9] Altermark B. Helland R. Moe E. Willassen N. P. Smalås A. (2008). Structural adaptation of endonuclease I from the cold-adapted and halophilic bacterium *Vibrio salmonicida*. Acta Crystallogr. D Biol. Crystallogr. 64, 368–376. 10.1107/S0907444908000097 18391403

[B10] Alvarez M. Zeelen J. P. Mainfroid V. Rentier-Delrue F. Martial J. A. Wyns L.. (1998). Triose-phosphate isomerase (TIM) of the psychrophilic bacterium *Vibrio marinus*. Kinetic and structural properties. J. Biol. Chem. 273, 2199–2206. 10.1074/jbc.273.4.2199 9442062

[B11] Angelaccio S. Dworkowski F. Bello A. Milano T. Capitani G. Pascarella S. (2014). Conformational transitions driven by pyridoxal−5′−phosphate uptake in the psychrophilic serine hydroxymethyltransferase from *Psychromonas ingrahamii*. Proteins 82, 2831–2841. 10.1002/prot.24646 25044250

[B12] Angelaccio S. Florio R. Consalvi V. Festa G. Pascarella S. (2012). Serine hydroxymethyltransferase from the cold adapted microorganism *Psychromonas ingrahamii*: a low temperature active enzyme with broad substrate specificity. Int. J. Mol. Sci. 13, 1314–1326. 10.3390/ijms13021314 22408393 PMC3291962

[B13] Arimori T. Ito A. Nakazawa M. Ueda M. Tamada T. (2013). Crystal structure of endo-1, 4-β-glucanase from *Eisenia fetida*. J. Synchrotron Radiat. 20, 884–889. 10.1107/S0909049513021110 24121333 PMC3795549

[B14] Arnold F. H. (2001). Combinatorial and computational challenges for biocatalyst design. Nature 409, 253–257. 10.1038/35051731 11196654

[B15] Arnold F. H. Wintrode P. L. Miyazaki K. Gershenson A. (2001). How enzymes adapt: lessons from directed evolution. Trends Biochem. Sci. 26, 100–106. 10.1016/S0968-0004(00)01755-2 11166567

[B16] Arnórsdóttir J. Kristjánsson M. M. Ficner R. (2005). Crystal structure of a subtilisin−like serine proteinase from a psychrotrophic Vibrio species reveals structural aspects of cold adaptation. FEBS J. 272, 832–845. 10.1111/j.1742-4658.2005.04523.x 15670163

[B17] Aurilia V. Rioux-Dubé J. Marabotti A. Pézolet M. D'Auria S. (2009). Structure and dynamics of cold-adapted enzymes as investigated by FT-IR spectroscopy and MD. The case of an esterase from *Pseudoalteromonas haloplanktis*. J. Phys. Chem. B 113, 7753–7761. 10.1021/jp901921r 19435327

[B18] Ásgeirsson B. Adalbjörnsson B. V. Gylfason G. A. (2007). Engineered disulfide bonds increase active-site local stability and reduce catalytic activity of a cold-adapted alkaline phosphatase. Biochim. Biophys. Acta 1774, 679–687. 10.1016/j.bbapap.2007.03.016 17493882

[B19] Bae E. Phillips G. N. Jr. (2004). Structures and analysis of highly homologous psychrophilic, mesophilic, and thermophilic adenylate kinases. J. Biol. Chem. 279, 28202–28208. 10.1074/jbc.M401865200 15100224

[B20] Bai W. Xue Y. Zhou C. Ma Y. (2012). Cloning, expression and characterization of a novel salt-tolerant xylanase from *Bacillus* sp. SN5. Biotechnol. Lett. 34, 2093–2099. 10.1007/s10529-012-1011-7 22864505

[B21] Bakunina I. Y. Balabanova L. A. Golotin V. A. Slepchenko L. V. Isakov V. V. Rasskazov V. A. (2014). Stereochemical course of hydrolytic reaction catalyzed by alpha-galactosidase from cold adaptable marine bacterium of genus Pseudoalteromonas. Front. Chem. 2:89. 10.3389/fchem.2014.00089 25353020 PMC4195319

[B22] Balabanova L. A. Bakunina I. Y. Nedashkovskaya O. I. Makarenkova I. D. Zaporozhets T. S. Besednova N. N.. (2010). Molecular characterization and therapeutic potential of a marine bacterium *Pseudoalteromonas* sp. KMM 701 alpha-galactosidase. Mar. Biotechnol. (NY) 12, 111–120. 10.1007/s10126-009-9205-2 19629597

[B23] Balasubramaniam D. Komives E. A. (2013). Hydrogen-exchange mass spectrometry for the study of intrinsic disorder in proteins. Biochim. Biophys. Acta 1834, 1202–1209. 10.1016/j.bbapap.2012.10.009 23099262 PMC3600394

[B24] Bauvois C. Jacquamet L. Huston A. L. Borel F. Feller G. Ferrer J. L. (2008). Crystal structure of the cold-active aminopeptidase from *Colwellia psychrerythraea*, a close structural homologue of the human bifunctional leukotriene A4 hydrolase. J. Biol. Chem. 283, 23315–23325. 10.1074/jbc.M802158200 18539590

[B25] Bell G. S. Russell R. J. Connaris H. Hough D. W. Danson M. J. Taylor G. L. (2002). Stepwise adaptations of citrate synthase to survival at life's extremes. Eur. J. Biochem. 269, 6250–6260. 10.1046/j.1432-1033.2002.03344.x 12473121

[B26] Bentahir M. Feller G. Aittaleb M. Lamotte-Brasseur J. Himri T. Chessa J. P.. (2000). Structural, kinetic, and calorimetric characterization of the cold-active phosphoglycerate kinase from the antarctic *Pseudomonas* sp. TACII18. J. Biol. Chem. 275, 11147–11153. 10.1074/jbc.275.15.11147 10753921

[B27] Berglund G. I. Willassen N. Hordvik A. Smalås A. (1995). Structure of native pancreatic elastase from North Atlantic salmon at 1.61 Å resolution. Acta Crystallogr. D Biol. Crystallogr. 51, 925–937. 10.1107/S0907444995004835 15299762

[B28] Berman H. M. Westbrook J. Feng Z. Gilliland G. Bhat T. N. Weissig H.. (2000). The protein data bank. Nucleic Acids Res. 28, 235–242. 10.1093/nar/28.1.235 10592235 PMC102472

[B29] Bjerga G. E. K. Lale R. Williamson A. K. (2016). Engineering low-temperature expression systems for heterologous production of cold-adapted enzymes. Bioengineered 7, 33–38. 10.1080/21655979.2015.1128589 26710170 PMC4878266

[B30] Bjerga G. E. K. Williamson A. K. (2015). Cold shock induction of recombinant Arctic environmental genes. BMC Biotechnol. 15:1. 10.1186/s12896-015-0185-1 26286037 PMC4544801

[B31] Borgi M. A. Khila M. Boudebbouze S. Aghajari N. Szukala F. Pons N.. (2014). The attractive recombinant phytase from *Bacillus licheniformi*s: biochemical and molecular characterization. Appl. Microbiol. Biotechnol. 98, 5937–5947. 10.1007/s00253-013-5421-9 24337251

[B32] Bornscheuer U. T. Huisman G. W. Kazlauskas R. J. Lutz S. Moore J. C. Robins K. (2012). Engineering the third wave of biocatalysis. Nature 485, 185–194. 10.1038/nature11117 22575958

[B33] Brault G. Shareck F. Hurtubise Y. Lepine F. Doucet N. (2012). Isolation and characterization of EstC, a new cold-active esterase from *Streptomyces coelicolor* A3(2). PLoS ONE 7:e32041. 10.1371/journal.pone.0032041 22396747 PMC3292560

[B34] Brunialti E. A. Gatti-Lafranconi P. Lotti M. (2011). Promiscuity, stability and cold adaptation of a newly isolated acylaminoacyl peptidase. Biochimie 93, 1543–1554. 10.1016/j.biochi.2011.05.010 21635934

[B35] Brzuszkiewicz A. Nowak E. Dauter Z. Dauter M. Cieœliñski H. Długołȩcka A.. (2009). Structure of EstA esterase from psychrotrophic *Pseudoalteromonas* sp. 643A covalently inhibited by monoethylphosphonate. Acta Crystallogr. Sect. F Struct. Biol. Cryst. Commun. 65, 862–865. 10.1107/S1744309109030826 19724118 PMC2795586

[B36] Bujacz A. Rutkiewicz-Krotewicz M. Nowakowska-Sapota K. Turkiewicz M. (2015). Crystal structure and enzymatic properties of a broad substrate-specificity psychrophilic aminotransferase from the Antarctic soil bacterium *Psychrobacter* sp. B6. Acta Crystallogr. D Biol. Crystallogr. 71, 632–645. 10.1107/S1399004714028016 25760611

[B37] Bukau B. Deuerling E. Pfund C. Craig E. A. (2000). Getting newly synthesized proteins into shape. Cell 101, 119–122. 10.1016/S0092-8674(00)80806-5 10786831

[B38] Buzzini P. Branda E. Goretti M. Turchetti B. (2012). Psychrophilic yeasts from worldwide glacial habitats: diversity, adaptation strategies and biotechnological potential. FEMS Microbiol. Ecol. 82, 217–241. 10.1111/j.1574-6941.2012.01348.x 22385361

[B39] Cavicchioli R. Charlton T. Ertan H. Omar S. M. Siddiqui K. Williams T. (2011). Biotechnological uses of enzymes from psychrophiles. Microb. Biotechnol. 4, 449–460. 10.1111/j.1751-7915.2011.00258.x 21733127 PMC3815257

[B40] Cavicchioli R. Siddiqui K. S. Andrews D. Sowers K. R. (2002). Low-temperature extremophiles and their applications. Curr. Opin. Biotechnol. 13, 253–261. 10.1016/S0958-1669(02)00317-8 12180102

[B41] Çelik A. Yetis G. (2012). An unusually cold active nitroreductase for prodrug activations. Bioorg. Med. Chem. 20, 3540–3550. 10.1016/j.bmc.2012.04.004 22546205

[B42] Cesarini S. Bofill C. Pastor F. J. Reetz M. T. Diaz P. (2012). A thermostable variant of *P. aeruginosa* cold-adapted LipC obtained by rational design and saturation mutagenesis. Process Biochem. 47, 2064–2071. 10.1016/j.procbio.2012.07.023

[B43] Chalmers M. J. Busby S. A. Pascal B. D. West G. M. Griffin P. R. (2011). Differential hydrogen/deuterium exchange mass spectrometry analysis of protein–ligand interactions. Expert Rev. Proteomics 8, 43–59. 10.1586/epr.10.109 21329427 PMC3113475

[B44] Chen R. Guo L. Dang H. (2010). Gene cloning, expression and characterization of a cold-adapted lipase from a psychrophilic deep-sea bacterium *Psychrobacter* sp. C18. World J. Microbiol. Biotechnol. 27, 431–441. 10.1007/s11274-010-0475-7

[B45] Chen S. Kaufman M. G. Miazgowicz K. L. Bagdasarian M. Walker E. D. (2013). Molecular characterization of a cold-active recombinant xylanase from *Flavobacterium johnsoniae* and its applicability in xylan hydrolysis. Bioresour. Technol. 128, 145–155. 10.1016/j.biortech.2012.10.087 23196234 PMC4106359

[B46] Cheng Y. Y. Qian Y. K. Li Z. F. Wu Z. H. Liu H. Li Y. Z. (2011). A novel cold-adapted lipase from *Sorangium cellulosum* strain So0157-2: gene cloning, expression, and enzymatic characterization. Int. J. Mol. Sci. 12, 6765–6780. 10.3390/ijms12106765 22072918 PMC3211009

[B47] Cipolla A. D'Amico S. Barumandzadeh R. Matagne A. Feller G. (2011). Stepwise adaptations to low temperature as revealed by multiple mutants of psychrophilic alpha-amylase from Antarctic Bacterium. J. Biol. Chem. 286, 38348–38355. 10.1074/jbc.M111.274423 21900238 PMC3207396

[B48] Cipolla A. Delbrassine F. Da Lage J. Feller G. (2012). Temperature adaptations in psychrophilic, mesophilic and thermophilic chloride-dependent alpha-amylases. Biochimie 94, 1943–1950. 10.1016/j.biochi.2012.05.013 22634328

[B49] Coker J. A. Brenchley J. E. (2006). Protein engineering of a cold-active β-galactosidase from *Arthrobacter* sp. SB to increase lactose hydrolysis reveals new sites affecting low temperature activity. Extremophiles 10, 515–524. 10.1007/s00792-006-0526-z 16736094

[B50] Collins T. Gerday C. Feller G. (2005). Xylanases, xylanase families and extremophilic xylanases. FEMS Microbiol. Rev. 29, 3–23. 10.1016/j.femsre.2004.06.005 15652973

[B51] Coquelle N. Fioravanti E. Weik M. Vellieux F. Madern D. (2007). Activity, stability and structural studies of lactate dehydrogenases adapted to extreme thermal environments. J. Mol. Biol. 374, 547–562. 10.1016/j.jmb.2007.09.049 17936781

[B52] Cowan D. A. Casanueva A. Stafford W. (2007). Ecology and biodiversity of cold-adapted microorganisms, in Physiology and Biochemistry of Extremophiles, eds Gerday C. Glansdorff N. (Washington, DC: American Society of Microbiology), 119–132.

[B53] Crespim E. Zanphorlin L. M. de Souza F. H. Diogo J. A. Gazolla A. C. Machado C. B.. (2016). A novel cold-adapted and glucose-tolerant GH1 beta-glucosidase from *Exiguobacterium antarcticum* B7. Int. J. Biol. Macromol. 82, 375–380. 10.1016/j.ijbiomac.2015.09.018 26475230

[B54] Cusano A. M. Parrilli E. Duilio A. Sannia G. Marino G. Tutino M. L. (2006b). Secretion of psychrophilic alpha-amylase deletion mutants in *Pseudoalteromonas haloplanktis* TAC125. FEMS Microbiol. Lett. 258, 67–71. 10.1111/j.1574-6968.2006.00193.x 16630257

[B55] Cusano A. M. Parrilli E. Marino G. Tutino M. L. (2006a). A novel genetic system for recombinant protein secretion in the Antarctic *Pseudoalteromonas haloplanktis* TAC125.Microb. Cell Fact. 5, 1. 10.1186/1475-2859-5-40 17169153 PMC1766363

[B56] D'Amico S. Claverie P. Collins T. Georlette D. Gratia E. Hoyoux A.. (2002a). Molecular basis of cold adaptation. Philos. Trans. R. Soc. Lond. B Biol. Sci. 357, 917–925. 10.1098/rstb.2002.1105 12171655 PMC1692995

[B57] D'Amico S. Gerday C. Feller G. (2002b). Dual effects of an extra disulfide bond on the activity and stability of a cold-adapted alpha-amylase. J. Biol. Chem. 277, 46110–46115. 10.1074/jbc.M207253200 12324460

[B58] D'Amico S. Gerday C. Feller G. (2003a). Temperature adaptation of proteins: engineering mesophilic-like activity and stability in a cold-adapted α-amylase. J. Mol. Biol. 332, 981–988. 10.1016/j.jmb.2003.07.014 14499602

[B59] D'Amico S. Marx J. C. Gerday C. Feller G. (2003b). Activity-stability relationships in extremophilic enzymes. J. Biol. Chem. 278, 7891–7896. 10.1074/jbc.M212508200 12511577

[B60] Davail S. Feller G. Narinx E. Gerday C. (1994). Cold adaptation of proteins. Purification, characterization, and sequence of the heat-labile subtilisin from the antarctic psychrophile *Bacillus* TA41. J. Biol. Chem. 269, 17448–17453. 8021248

[B61] Davlieva M. Shamoo Y. (2009). Structure and biochemical characterization of an adenylate kinase originating from the psychrophilic organism *Marinibacillus marinus*. Acta Crystallogr. Sect. F Struct. Biol. Cryst. Commun. 65, 751–756. 10.1107/S1744309109024348 19652331 PMC2720325

[B62] de Backer M. McSweeney S. Rasmussen H. B. Riise B. W. Lindley P. Hough E. (2002). The 1.9 Å crystal structure of heat-labile shrimp alkaline phosphatase. J. Mol. Biol. 318, 1265–1274. 10.1016/S0022-2836(02)00035-9 12083516

[B63] De Maayer P. Anderson D. Cary C. Cowan D. A. (2014). Some like it cold: understanding the survival strategies of psychrophiles. EMBO Rep. 15, 508–517. 10.1002/embr.201338170 24671034 PMC4210084

[B64] de Marco A. (2007). Protocol for preparing proteins with improved solubility by co-expressing with molecular chaperones in *Escherichia coli.* Nat. Protoc. 2, 2632–2639. 10.1038/nprot.2007.400 17948006

[B65] Deming J. W. (2002). Psychrophiles and polar regions. Curr. Opin. Microbiol. 5, 301–309. 10.1016/S1369-5274(02)00329-6 12057685

[B66] Deng A. Wu J. Zhang G. Wen T. (2011). Molecular and structural characterization of a surfactant-stable high-alkaline protease AprB with a novel structural feature unique to subtilisin family. Biochimie 93, 783–791. 10.1016/j.biochi.2011.01.011 21281692

[B67] de Pascale D. Giuliani M. De Santi C. Bergamasco N. Amoresano A. Carpentieri A. (2010). PhAP protease from *Pseudoalteromonas haloplanktis* TAC125: gene cloning, recombinant production in *E. coli* and enzyme characterization. Polar Sci. 4, 285–294. 10.1016/j.polar.2010.03.009

[B68] De Santi C. Leiros H. K. Di Scala A. de Pascale D. Altermark B. Willassen N. P. (2016). Biochemical characterization and structural analysis of a new cold-active and salt-tolerant esterase from the marine bacterium *Thalassospira* sp. Extremophiles 20, 323–336. 10.1007/s00792-016-0824-z 27016194

[B69] De Santi C. Tedesco P. Ambrosino L. Altermark B. Willassen N. P. de Pascale D. (2014). A new alkaliphilic cold-active esterase from the psychrophilic marine bacterium *Rhodococcus* sp.: functional and structural studies and biotechnological potential. Appl. Biochem. Biotechnol. 172, 3054–3068. 10.1007/s12010-013-0713-1 24488777

[B70] De Vos D. Xu Y. Hulpiau P. Vergauwen B. Van Beeumen J. J. (2007). Structural investigation of cold activity and regulation of aspartate carbamoyltransferase from the extreme psychrophilic bacterium *Moritella profunda*. J. Mol. Biol. 365, 379–395. 10.1016/j.jmb.2006.09.064 17070547

[B71] Dhar H. Kasana R. C. Dutt S. Gulati A. (2015). Cloning and expression of low temperature active endoglucanase EG5C from *Paenibacillus s*p. IHB B 3084. Int. J. Biol. Macromol. 81, 259–266. 10.1016/j.ijbiomac.2015.07.060 26234579

[B72] Do H. Kim S. J. Lee C. W. Kim H. Park H. H. Kim H. M.. (2015a). Crystal structure of UbiX, an aromatic acid decarboxylase from the psychrophilic bacterium *Colwellia psychrerythraea* that undergoes FMN-induced conformational changes. Sci. Rep. 5:8196. 10.1038/srep08196 25645665 PMC4316190

[B73] Do H. Yun J. S. Lee C. W. Choi Y. J. Kim H. Y. Kim Y. J.. (2015b). Crystal structure and comparative sequence analysis of GmhA from *Colwellia psychrerythraea* strain 34H provides insight into functional similarity with DiaA. Mol. Cells 38, 1086–1095. 10.14348/molcells.2015.0191 26612680 PMC4697000

[B74] Dong Q. Yan X. Zheng M. Yang Z. (2014). Characterization of an extremely thermostable but cold-adaptive beta-galactosidase from the hyperthermophilic archaeon *Pyrococcus furiosus* for use as a recombinant aggregation for batch lactose degradation at high temperature. J. Biosci. Bioeng. 117, 706–710. 10.1016/j.jbiosc.2013.12.002 24462527

[B75] Dornez E. Verjans P. Arnaut F. Delcour J. A. Courtin C. M. (2011). Use of psychrophilic xylanases provides insight into the xylanase functionality in bread making. J. Agric. Food Chem. 59, 9553–9562. 10.1021/jf201752g 21806059

[B76] Doukyu N. Ogino H. (2010). Organic solvent-tolerant enzymes. Biochem. Eng. J. 48, 270–282. 10.1016/j.bej.2009.09.009

[B77] Dunn P. J. (2012). The importance of green chemistry in process research and development. Chem. Soc. Rev. 41, 1452–1461. 10.1039/C1CS15041C 21562677

[B78] Eftink M. R. Ghiron C. (1976). Exposure of tryptophanyl residues in proteins. Quantitative determination by fluorescence quenching studies. Biochemistry 15, 672–680. 10.1021/bi00648a035 1252418

[B79] Eftink M. R. Ghiron C. A. (1975). Dynamics of a protein matrix revealed by fluorescence quenching. Proc. Natl. Acad. Sci. U.S.A. 72, 3290–3294. 10.1073/pnas.72.9.3290 810800 PMC432977

[B80] Ekkers D. M. Cretoiu M. S. Kielak A. M. Elsas J. D. (2012). The great screen anomaly–a new frontier in product discovery through functional metagenomics. Appl. Microbiol. Biotechnol. 93, 1005–1020. 10.1007/s00253-011-3804-3 22189864 PMC3264863

[B81] Elleuche S. Qoura F. M. Lorenz U. Rehn T. Brück T. Antranikian G. (2015). Cloning, expression and characterization of the recombinant cold-active type-I pullulanase from Shewanella arctica. J. Mol. Catal. B Enzym. 116, 70–77. 10.1016/j.molcatb.2015.03.001

[B82] Elleuche S. Schröder C. Sahm K. Antranikian G. (2014). Extremozymes—biocatalysts with unique properties from extremophilic microorganisms. Curr. Opin. Biotechnol. 29, 116–123. 10.1016/j.copbio.2014.04.003 24780224

[B83] Esteban-Torres M. Mancheno J. M. de las Rivas B. Munoz R. (2014a). Characterization of a cold-active esterase from *Lactobacillus plantarum* suitable for food fermentations. J. Agric. Food Chem. 62, 5126–5132. 10.1021/jf501493z 24856291

[B84] Esteban-Torres M. Santamaría L. de las Rivas B. Muñoz R. (2014b). Characterisation of a cold-active and salt-tolerant esterase from *Lactobacillus plantarum* with potential application during cheese ripening. Int. Dairy J. 39, 312–315. 10.1016/j.idairyj.2014.08.004

[B85] Evstigneeva Z. Solov'eva N. Sidel'nikova L. (2001). Structures and functions of chaperones and chaperonins (review). Appl. Biochem. Microbiol. 37, 1–13. 10.1023/A:1002835921817 11234405

[B86] Eyring H. (1935). The activated complex and the absolute rate of chemical reactions. Chem. Rev. 17, 65–77. 10.1021/cr60056a006

[B87] Fan Y. Hua X. Zhang Y. Feng Y. Shen Q. Dong J.. (2015). Cloning, expression and structural stability of a cold-adapted beta-galactosidase from *Rahnella* sp. R3. Protein Expr. Purif. 115, 158–164. 10.1016/j.pep.2015.07.001 26145832

[B88] Fedøy A. Yang N. Martinez A. Leiros H. S. Steen I. H. (2007). Structural and functional properties of isocitrate dehydrogenase from the psychrophilic bacterium *Desulfotalea psychrophila* reveal a cold-active enzyme with an unusual high thermal stability. J. Mol. Biol. 372, 130–149. 10.1016/j.jmb.2007.06.040 17632124

[B89] Feller G. (2003). Molecular adaptations to cold in psychrophilic enzymes. Cell. Mol. Life Sci. 60, 648–662. 10.1007/s00018-003-2155-3 12785714 PMC11138853

[B90] Feller G. (2010). Protein stability and enzyme activity at extreme biological temperatures. J. Phys. Condens. Matter 22:323101. 10.1088/0953-8984/22/32/323101 21386475

[B91] Feller G. (2013). Psychrophilic enzymes: from folding to function and biotechnology. Scientifica 2013:512840. 10.1155/2013/512840 24278781 PMC3820357

[B92] Feller G. Gerday C. (1997). Psychrophilic enzymes: molecular basis of cold adaptation. Cell. Mol. Life Sci. 53, 830–841. 10.1007/s000180050103 9413552 PMC11147173

[B93] Feller G. Gerday C. (2003). Psychrophilic enzymes: hot topics in cold adaptation. Nat. Rev. Microbiol. 1, 200–208. 10.1038/nrmicro773 15035024

[B94] Feller G. Le Bussy O. Gerday C. (1998). Expression of psychrophilic genes in mesophilic hosts: assessment of the folding state of a recombinant alpha-amylase. Appl. Environ. Microbiol. 64, 1163–1165. 9501457 10.1128/aem.64.3.1163-1165.1998 PMC106386

[B95] Feller G. Payan F. Theys F. Qian M. Haser R. Gerday C. (1994). Stability and structural analysis of α−amylase from the antarctic psychrophile *Alteromonas haloplanctis* A23. Eur. J. Biochem. 222, 441–447. 10.1111/j.1432-1033.1994.tb18883.x 8020481

[B96] Ferrer M. Chernikova T. N. Timmis K. N. Golyshin P. N. (2004b). Expression of a temperature-sensitive esterase in a novel chaperone-based *Escherichia coli* strain. Appl. Environ. Microbiol. 70, 4499–4504. 10.1128/AEM.70.8.4499-4504.2004 15294778 PMC492381

[B97] Ferrer M. Chernikova T. N. Yakimov M. M. Golyshin P. N. Timmis K. N. (2003). Chaperonins govern growth of *Escherichia coli* at low temperatures. Nat. Biotechnol. 21, 1266–1267. 10.1038/nbt1103-1266 14595348

[B98] Ferrer M. Golyshina O. Beloqui A. Golyshin P. N. (2007). Mining enzymes from extreme environments. Curr. Opin. Microbiol. 10, 207–214. 10.1016/j.mib.2007.05.004 17548239

[B99] Ferrer M. Lünsdorf H. Chernikova T. N. Yakimov M. Timmis K. N. Golyshin P. N. (2004a). Functional consequences of single: double ring transitions in chaperonins: life in the cold. Mol. Microbiol. 53, 167–182. 10.1111/j.1365-2958.2004.04077.x 15225312

[B100] Fields P. A. Somero G. N. (1998). Hot spots in cold adaptation: localized increases in conformational flexibility in lactate dehydrogenase A4 orthologs of Antarctic notothenioid fishes. Proc. Natl. Acad. Sci. U.S.A. 95, 11476–11481. 10.1073/pnas.95.19.11476 9736762 PMC21668

[B101] Florczak T. Daroch M. Wilkinson M. C. Bialkowska A. Bates A. D. Turkiewicz M.. (2013). Purification, characterisation and expression in *Saccharomyces cerevisiae* of LipG7 an enantioselective, cold-adapted lipase from the Antarctic filamentous fungus *Geomyces* sp. P7 with unusual thermostability characteristics. Enzyme Microb. Technol. 53, 18–24. 10.1016/j.enzmictec.2013.03.021 23683700

[B102] Fu J. Leiros H. S. de Pascale D. Johnson K. A. Blencke H. Landfald B. (2013). Functional and structural studies of a novel cold-adapted esterase from an Arctic intertidal metagenomic library. Appl. Microbiol. Biotechnol. 97, 3965–3978. 10.1007/s00253-012-4276-9 22832985

[B103] Garsoux G. Lamotte J. Gerday C. Feller G. (2004). Kinetic and structural optimization to catalysis at low temperatures in a psychrophilic cellulase from the Antarctic bacterium *Pseudoalteromonas haloplanktis*. Biochem. J. 384, 247–253. 10.1042/BJ20040325 15287848 PMC1134107

[B104] Gatti-Lafranconi P. Caldarazzo S. M. Villa A. Alberghina L. Lotti M. (2008). Unscrambling thermal stability and temperature adaptation in evolved variants of a cold−active lipase. FEBS Lett. 582, 2313–2318. 10.1016/j.febslet.2008.05.037 18534193

[B105] Georlette D. Blaise V. Collins T. D'Amico S. Gratia E. Hoyoux A.. (2004). Some like it cold: biocatalysis at low temperatures. FEMS Microbiol. Rev. 28, 25–42. 10.1016/j.femsre.2003.07.003 14975528

[B106] Georlette D. Damien B. Blaise V. Depiereux E. Uversky V. N. Gerday C.. (2003). Structural and functional adaptations to extreme temperatures in psychrophilic, mesophilic, and thermophilic DNA ligases. J. Biol. Chem. 278, 37015–37023. 10.1074/jbc.M305142200 12857762

[B107] Gerday C. Aittaleb M. Arpigny J. L. Baise E. Chessa J. P. Garsoux G.. (1997). Psychrophilic enzymes: a thermodynamic challenge. Biochim. Biophys. Acta 1342, 119–131. 10.1016/S0167-4838(97)00093-9 9392521

[B108] Gerday C. Aittaleb M. Bentahir M. Chessa J. P. Claverie P. Collins T.. (2000). Cold-adapted enzymes: from fundamentals to biotechnology. Trends Biotechnol. 18, 103–107. 10.1016/S0167-7799(99)01413-4 10675897

[B109] Gerike U. Danson M. J. Hough D. W. (2001). Cold-active citrate synthase: mutagenesis of active-site residues. Protein Eng. 14, 655–661. 10.1093/protein/14.9.655 11707611

[B110] Gianese G. Argos P. Pascarella S. (2001). Structural adaptation of enzymes to low temperatures. Protein Eng. 14, 141–148. 10.1093/protein/14.3.141 11342709

[B111] Gianese G. Bossa F. Pascarella S. (2002). Comparative structural analysis of psychrophilic and meso−and thermophilic enzymes. Proteins 47, 236–249. 10.1002/prot.10084 11933070

[B112] Golotin V. Balabanova L. Likhatskaya G. Rasskazov V. (2015). Recombinant production and characterization of a highly active alkaline phosphatase from marine bacterium *Cobetia marina*. Mar. Biotechnol. 17, 130–143. 10.1007/s10126-014-9601-0 25260971

[B113] Gomes J. Steiner W. (2004). The biocatalytic potential of extremophiles and extremozymes. Food Technol. Biotechnol. 42, 223–235.

[B114] Gong J. Lu Z. Li H. Zhou Z. Shi J. Xu Z. (2013). Metagenomic technology and genome mining: emerging areas for exploring novel nitrilases. Appl. Microbiol. Biotechnol. 97, 6603–6611. 10.1007/s00253-013-4932-8 23801047

[B115] Goomber S. Kumar A. Kaur J. (2016a). Disruption of N terminus long range non covalent interactions shifted temp. opt 25°C to cold: evolution of point mutant Bacillus lipase by error prone PCR. Gene 576, 237–243. 10.1016/j.gene.2015.10.006 26456196

[B116] Goomber S. Kumar A. Singh R. Kaur J. (2016b). Point mutation ile137-Met near surface conferred psychrophilic behaviour and improved catalytic efficiency to bacillus lipase of 1.4 subfamily. Appl. Biochem. Biotechnol. 178, 753–765. 10.1007/s12010-015-1907-5 26520838

[B117] Guo B. Li P. Y. Yue Y. S. Zhao H. L. Dong S. Song X. Y.. (2013). Gene cloning, expression and characterization of a novel xylanase from the marine bacterium, *Glaciecola mesophila* KMM241. Mar. Drugs 11, 1173–1187. 10.3390/md11041173 23567318 PMC3705397

[B118] Hartl F. U. Bracher A. Hayer-Hartl M. (2011). Molecular chaperones in protein folding and proteostasis. Nature 475, 324–332. 10.1038/nature10317 21776078

[B119] Hayashi K. Kojima C. (2008). pCold-GST vector: a novel cold-shock vector containing GST tag for soluble protein production. Protein Expr. Purif. 62, 120–127. 10.1016/j.pep.2008.07.007 18694833

[B120] Hayashi K. Kojima C. (2010). Efficient protein production method for NMR using soluble protein tags with cold shock expression vector. J. Biomol. NMR 48, 147–155. 10.1007/s10858-010-9445-5 20844927

[B121] Hayashi T. Matsuzaki W. Takada Y. (2014). Characterization of chimeric and mutated isocitrate lyases of a mesophilic nitrogen-fixing bacterium, Azotobacter vinelandii, and a psychrophilic bacterium, *Colwellia maris. Biosci. Biotechnol*. Biochem. 78, 195–201. 10.1080/09168451.2014.882744 25036671

[B122] Helland R. Larsen A. N. Smalås A. O. Willassen N. P. (2006). The 1.8 Å crystal structure of a proteinase K−like enzyme from a psychrotroph Serratia species. FEBS J. 273, 61–71. 10.1111/j.1742-4658.2005.05040.x 16367748

[B123] Helland R. Larsen R. L. Ásgeirsson B. (2009). The 1.4 Å crystal structure of the large and cold-active *Vibrio* sp. alkaline phosphatase. Biochim. Biophys. Acta 1794, 297–308. 10.1016/j.bbapap.2008.09.020 18977465

[B124] Höppner A. Widderich N. Lenders M. Bremer E. Smits S. H. (2014). Crystal structure of the ectoine hydroxylase, a snapshot of the active site. J. Biol. Chem. 289, 29570–29583. 10.1074/jbc.M114.576769 25172507 PMC4207974

[B125] Hoyoux A. Jennes I. Dubois P. Genicot S. Dubail F. Francois J. M.. (2001). Cold-adapted beta-galactosidase from the Antarctic psychrophile *Pseudoalteromonas haloplanktis*. Appl. Environ. Microbiol. 67, 1529–1535. 10.1128/AEM.67.4.1529-1535.2001 11282601 PMC92765

[B126] Huang J. L. Bao L. X. Zou H. Y. Che S. G. Wang G. X. (2012). High-level production of a cold-active B-mannanase from *Bacillus subtilis* Bs5 and its molecular cloning and expression. Mol. Genet. Microbiol. Virol. 27, 147–153. 10.3103/S0891416812040039 23248847

[B127] Huston A. L. (2008). Biotechnological aspects of cold-adapted enzymes, in Psychrophiles: From Biodiversity to Biotechnology, eds Margesin R. Schinner F. Marx J. C. Gerday C.(Berlin; Heidelberg: Springer), 347–363.

[B128] Huston A. L. Haeggström J. Z. Feller G. (2008). Cold adaptation of enzymes: structural, kinetic and microcalorimetric characterizations of an aminopeptidase from the Arctic psychrophile *Colwellia psychrerythraea* and of human leukotriene A 4 hydrolase. Biochim. Biophys. Acta 1784, 1865–1872. 10.1016/j.bbapap.2008.06.002 18599387

[B129] Illias R. M. Ramli A. N. M. Low K. O. Mahadi N. M. Murad A. M. A. Rabu A. (2014). Heterologous expression of proteins from cold-adapted yeasts in suitable hosts: methods and applications, in Cold-Adapted Yeasts, eds Buzzini P. Margesin R.(Berlin; Heidelberg: Springer), 481–496.

[B130] Isaksen G. V. Åqvist J. Brandsdal B. O. (2016). Enzyme surface rigidity tunes the temperature dependence of catalytic rates. Proc. Natl. Acad. Sci. U.S.A. 113, 7822–7827. 10.1073/pnas.1605237113 27354533 PMC4948340

[B131] Jaremko L. Jaremko M. Elfaki I. Mueller J. W. Ejchart A. Bayer P.. (2011). Structure and dynamics of the first archaeal parvulin reveal a new functionally important loop in parvulin-type prolyl isomerases. J. Biol. Chem. 286, 6554–6565. 10.1074/jbc.M110.160713 21138844 PMC3057832

[B132] Jiang H. Zhang S. Gao H. Hu N. (2016). Characterization of a cold-active esterase from *Serratia* sp. and improvement of thermostability by directed evolution. BMC Biotechnol. 16:7. 10.1186/s12896-016-0235-3 26800680 PMC4722774

[B133] Johnsen M. G. Hansen O. C. Stougaard P. (2010). Isolation, characterization and heterologous expression of a novel chitosanase from *Janthinobacterium* sp. strain 4239. Microb. Cell Fact. 9, 1–9. 10.1186/1475-2859-9-5 20096097 PMC2835661

[B134] Joseph B. Ramteke P. W. Thomas G. (2008). Cold active microbial lipases: some hot issues and recent developments. Biotechnol. Adv. 26, 457–470. 10.1016/j.biotechadv.2008.05.003 18571355

[B135] Jung S. Jeong D. G. Lee M. S. Lee J. Kim H. Ryu S. E.. (2008). Structural basis for the cold adaptation of psychrophilic M37 lipase from *Photobacterium lipolyticum*. Proteins 71, 476–484. 10.1002/prot.21884 18186467

[B136] Kamarudin N. H. Rahman R. N. Ali M. S. Leow T. C. Basri M. Salleh A. B. (2014). A new cold-adapted, organic solvent stable lipase from mesophilic *Staphylococcus epidermidis* AT2. Protein J. 33, 296–307. 10.1007/s10930-014-9560-3 24777627

[B137] Karan R. Capes M. D. DasSarma P. DasSarma S. (2013). Cloning, overexpression, purification, and characterization of a polyextremophilic β-galactosidase from the Antarctic haloarchaeon Halorubrum lacusprofundi. BMC Biotechnol. 13:3. 10.1186/1472-6750-13-3 23320757 PMC3556326

[B138] Karan R. Capes M. D. DasSarma S. (2012). Function and biotechnology of extremophilic enzymes in low water activity. Aquat. Biosyst. 8, 1. 10.1186/2046-9063-8-4 22480329 PMC3310334

[B139] Karlsen S. Hough E. Olsen R. L. (1998). Structure and proposed amino-acid sequence of a pepsin from Atlantic cod (*Gadus morhua*). Acta Crystallogr D Biol. Crystallogr. 54, 32–46. 10.1107/S090744499700810X 9761815

[B140] Kazlauskas R. Lutz S. (2009). Engineering enzymes by ‘intelligent’ design. Curr. Opin. Chem. Biol. 13, 1–2. 10.1016/j.cbpa.2009.02.022 19272831 PMC2695408

[B141] Khurana J. Kumar R. Kumar A. Singh K. Singh R. Kaur J. (2015). New insight into old bacillus lipase: solvent stable mesophilic lipase demonstrating enzyme activity towards cold. J. Mol. Microbiol. Biotechnol. 25, 340–348. 10.1159/000439276 26488405

[B142] Kille S. Acevedo-Rocha C. Parra L. P. Zhang Z. Opperman D. J. Reetz M. T.. (2013). Reducing codon redundancy and screening effort of combinatorial protein libraries created by saturation mutagenesis. ACS Synth. Biol. 2, 83–92. 10.1021/sb300037w 23656371

[B143] Kim H. Wi A. R. Jeon B. W. Lee J. H. Shin S. C. Park H.. (2015). Cold adaptation of a psychrophilic chaperonin from *Psychrobacter* sp. and its application for heterologous protein expression. Biotechnol. Lett. 37, 1887–1893. 10.1007/s10529-015-1860-y 26003095

[B144] Kim S. Y. Hwang K. Y. Kim S. H. Sung H. C. Han Y. S. Cho Y. (1999). Structural basis for cold adaptation. Sequence, biochemical properties, and crystal structure of malate dehydrogenase from a psychrophile Aquaspirillium arcticum. J. Biol. Chem. 274, 11761–11767. 10.1074/jbc.274.17.11761 10206992

[B145] Kim Y. (2012). Gene cloning and characterization of a cold-adapted esterase from *Acinetobacter venetianus* V28. J. Microbiol. Biotechnol. 22, 1245–1252. 10.4014/jmb.1201.01045 22814499

[B146] Kim Y. Heo Y. L. Nam B. Kim D. Jee Y. Lee S. (2013). Molecular cloning, purification, and characterization of a cold-adapted esterase from *Photobacterium* sp. MA1-3. Fish. Aquat. Sci. 16, 311–318. 10.5657/FAS.2013.0311

[B147] Kim Y. Park I. Kim H. Nam B. Jeong Kong H. Kim W.. (2011). A novel cold-adapted esterase from *Salinisphaera* sp. P7-4: gene cloning, overproduction, and characterization. J. Gen. Appl. Microbiol. 57, 357–364. 22353741 10.2323/jgam.57.357

[B148] Knoblauch C. Jorgensen B. B. Harder J. (1999). Community size and metabolic rates of psychrophilic sulfate-reducing bacteria in Arctic marine sediments. Appl. Environ. Microbiol. 65, 4230–4233. 10473441 10.1128/aem.65.9.4230-4233.1999 PMC99766

[B149] Ko J. K. Ko H. Kim K. H. Choi I. G. (2016). Characterization of the biochemical properties of recombinant Xyn10C from a marine bacterium, *Saccharophagus degradans* 2-40. Bioprocess Biosyst. Eng. 39, 677–684. 10.1007/s00449-016-1548-2 26809714

[B150] Korman T. P. Bowie J. U. (2012). Crystal structure of Proteus mirabilis lipase, a novel lipase from the Proteus/psychrophilic subfamily of lipase family I. 1. PLoS One 7:e52890. 10.1371/journal.pone.0052890 23300806 PMC3530535

[B151] Kosugi T. Hayashi S. (2011). Local entropy difference upon a substrate binding of a psychrophilic α-amylase and a mesophilic homologue. Chem. Phys. Lett. 501, 517–522. 10.1016/j.cplett.2010.11.059

[B152] Kosugi T. Hayashi S. (2012). Crucial role of protein flexibility in formation of a stable reaction transition state in an α-amylase catalysis. J. Am. Chem. Soc. 134, 7045–7055. 10.1021/ja212117m 22468622

[B153] Kovacic F. Mandrysch A. Poojari C. Strodel B. Jaeger K. E. (2016). Structural features determining thermal adaptation of esterases. Protein Eng. Des. Sel. 29, 65–76. 10.1093/protein/gzv061 26647400 PMC5943684

[B154] Kube M. Chernikova T. N. Al-Ramahi Y. Beloqui A. Lopez-Cortez N. Guazzaroni M.. (2013). Genome sequence and functional genomic analysis of the oil-degrading bacterium *Oleispira antarctica*. Nat. Commun. 4:2156. 10.1038/ncomms3156 23877221 PMC3759055

[B155] Kumar A. Dhar K. Kanwar S. S. Arora P. K. (2016). Lipase catalysis in organic solvents: advantages and applications. Biol. Proced. Online 18, 1. 10.1186/s12575-016-0033-2 26766927 PMC4711063

[B156] Kumar S. Nussinov R. (2004). Different roles of electrostatics in heat and in cold: adaptation by citrate synthase. Chembiochem 5, 280–290. 10.1002/cbic.200300627 14997520

[B157] Kumar V. Yedavalli P. Gupta V. Rao N. M. (2014). Engineering lipase A from mesophilic *Bacillus subtilis* for activity at low temperatures. Protein Eng. Des. Sel. 27, 73–82. 10.1093/protein/gzt064 24402332

[B158] Laidler K. J. (1984). The development of the Arrhenius equation. J. Chem. Educ. 61:494 10.1021/ed061p494

[B159] Lan D. M. Yang N. Wang W. K. Shen Y. F. Yang B. Wang Y. H. (2011). A novel cold-active lipase from *Candida albicans*: cloning, expression and characterization of the recombinant enzyme. Int. J. Mol. Sci. 12, 3950–3965. 10.3390/ijms12063950 21747717 PMC3131601

[B160] Lee C. Kim J. Hong S. Goo B. Lee S. Jang S. H. (2013). Cloning, expression, and characterization of a recombinant esterase from cold-adapted *Pseudomonas mandelii*. Appl. Biochem. Biotechnol. 169, 29–40. 10.1007/s12010-012-9947-6 23117417

[B161] Lee Y. S. (2016). Isolation and characterization of a novel cold-adapted esterase, MtEst45, from *Microbulbifer thermotolerans* DAU221. Front. Microbiol. 7:218. 10.3389/fmicb.2016.00218 26973604 PMC4773448

[B162] Leiros H. K. Pey A. L. Innselset M. Moe E. Leiros I. Steen I. H.. (2007). Structure of phenylalanine hydroxylase from *Colwellia psychrerythraea* 34H, a monomeric cold active enzyme with local flexibility around the active site and high overall stability. J. Biol. Chem. 282, 21973–21986. 10.1074/jbc.M610174200 17537732

[B163] Leiros I. Moe E. Lanes O. Smalås A. O. Willassen N. P. (2003). The structure of uracil-DNA glycosylase from Atlantic cod (*Gadus morhua*) reveals cold-adaptation features. Acta Crystallogr. D Biol. Crystallogr. 59, 1357–1365. 10.1107/S0907444903011144 12876336

[B164] Lemak S. Tchigvintsev A. Petit P. Flick R. Singer A. U. Brown G.. (2012). Structure and activity of the cold-active and anion-activated carboxyl esterase OLEI01171 from the oil-degrading marine bacterium *Oleispira antarctica*. Biochem. J. 445, 193–203. 10.1042/BJ20112113 22519667 PMC4127636

[B165] Leung D. W. Chen E. Goeddel D. V. (1989). A method for random mutagenesis of a defined DNA segment using a modified polymerase chain reaction. 1, 11–15.

[B166] Li M. Yang L. R. Xu G. Wu J. P. (2016a). Cloning and characterization of a novel lipase from *Stenotrophomonas maltophilia* GS11: the first member of a new bacterial lipase family XVI. J. Biotechnol. 228, 30–36. 10.1016/j.jbiotec.2016.04.034 27117245

[B167] Li W. Xue Y. Li J. Yuan J. Wang X. Fang W.. (2016b). A cold-adapted and glucose-stimulated type II alpha-glucosidase from a deep-sea bacterium *Pseudoalteromonas* sp. K8. Biotechnol. Lett. 38, 345–349. 10.1007/s10529-015-1987-x 26564409

[B168] Lian K. Leiros H. K. Moe E. (2015). MutT from the fish pathogen Aliivibrio salmonicida is a cold-active nucleotide-pool sanitization enzyme with unexpectedly high thermostability. FEBS Open Bio 5, 107–116. 10.1016/j.fob.2015.01.006 25737836 PMC4338371

[B169] Liang Z. Tsigos I. Lee T. Bouriotis V. Resing K. A. Ahn N. G.. (2004). Evidence for increased local flexibility in psychrophilic alcohol dehydrogenase relative to its thermophilic homologue. Biochemistry 43, 14676–14683. 10.1021/bi049004x 15544338

[B170] Liu Q. Wang Y. Luo H. Wang L. Shi P. Huang H.. (2015). Isolation of a novel cold-active family 11 Xylanase from the filamentous fungus *Bispora antennata* and deletion of its N-terminal amino acids on thermostability. Appl. Biochem. Biotechnol. 175, 925–936. 10.1007/s12010-014-1344-x 25351632

[B171] Liu X. Huang Z. Zhang X. Shao Z. Liu Z. (2014). Cloning, expression and characterization of a novel cold-active and halophilic xylanase from *Zunongwangia profunda*. Extremophiles 18, 441–450. 10.1007/s00792-014-0629-x 24464289

[B172] Lonhienne T. Gerday C. Feller G. (2000). Psychrophilic enzymes: revisiting the thermodynamic parameters of activation may explain local flexibility. Biochim. Biophys. Acta 1543, 1–10. 10.1016/S0167-4838(00)00210-7 11087936

[B173] Lonhienne T. Zoidakis J. Vorgias C. E. Feller G. Gerday C. Bouriotis V. (2001). Modular structure, local flexibility and cold-activity of a novel chitobiase from a psychrophilic Antarctic bacterium. J. Mol. Biol. 310, 291–297. 10.1006/jmbi.2001.4774 11428890

[B174] López-Iglesias M. Gotor-Fernández V. (2015). Recent advances in biocatalytic promiscuity: hydrolase-catalyzed reactions for nonconventional transformations. Chem. Rec. 15, 743–759. 10.1002/tcr.201500008 26147872

[B175] López-López O. E. Cerdan M. I. Gonzalez Siso M. (2014). New extremophilic lipases and esterases from metagenomics. Curr. Protein Pept. Sci. 15, 445–455. 10.2174/1389203715666140228153801 24588890 PMC4093774

[B176] Maes D. Zeelen J. P. Thanki N. Beaucamp N. Alvarez M. Thi M. H. D.. (1999). The crystal structure of triosephosphate isomerase (TIM) from *Thermotoga maritima*: a comparative thermostability structural analysis of ten different TIM structures. Proteins 37, 441–453. 10591103

[B177] Maiangwa J. Ali M. S. M. Salleh A. B. Rahman R. N. Shariff F. M. Leow T. C. (2015). Adaptational properties and applications of cold-active lipases from psychrophilic bacteria. Extremophiles 19, 235–247. 10.1007/s00792-014-0710-5 25472009

[B178] Malecki P. H. Raczynska J. E. Vorgias C. E. Rypniewski W. (2013). Structure of a complete four-domain chitinase from *Moritella marina*, a marine psychrophilic bacterium. Acta Crystallogr. D Biol. Crystallogr. 69, 821–829. 10.1107/S0907444913002011 23633591

[B179] Mao Y. Yin Y. Zhang L. Alias S. A. Gao B. Wei D. (2015). Development of a novel Aspergillus uracil deficient expression system and its application in expressing a cold-adapted α-amylase gene from Antarctic fungi *Geomyces pannorum*. Process Biochem. 50, 1581–1590. 10.1016/j.procbio.2015.06.016

[B180] Margesin R. Feller G. (2010). Biotechnological applications of psychrophiles. Environ. Technol. 31, 835–844. 10.1080/09593331003663328 20662375

[B181] Margesin R. Schinner F. Marx J. Gerday C. (2007). Psychrophiles: From Biodiversity to Biotechnology. Berlin: Springer Science & Business Media.

[B182] Marx J. C. Blaise V. Collins T. D'Amico S. Delille D. Gratia E.. (2004). A perspective on cold enzymes: current knowledge and frequently asked questions. Cell. Mol. Biol. (Noisy-le-grand) 50, 643–655. 15559980

[B183] Marx J. C. Collins T. D'Amico S. Feller G. Gerday C. (2007). Cold-adapted enzymes from marine Antarctic microorganisms. Mar. Biotechnol. (NY) 9, 293–304. 10.1007/s10126-006-6103-8 17195087

[B184] Matsuura A. Yao M. Aizawa T. Koganesawa N. Masaki K. Miyazawa M.. (2002). Structural analysis of an insect lysozyme exhibiting catalytic efficiency at low temperatures. Biochemistry 41, 12086–12092. 10.1021/bi016099j 12356308

[B185] Mavromatis K. Feller G. Kokkinidis M. Bouriotis V. (2003). Cold adaptation of a psychrophilic chitinase: a mutagenesis study. Protein Eng. 16, 497–503. 10.1093/protein/gzg069 12915727

[B186] Medina E. Córdova C. Villalobos P. Reyes J. Komives E. A. Ramírez-Sarmiento C. A.. (2016). Three-dimensional domain swapping changes the folding mechanism of the forkhead domain of foxP1. Biophys. J. 110, 2349–2360. 10.1016/j.bpj.2016.04.043 27276253 PMC4922571

[B187] Merlino A. Krauss I. R. Castellano I. De Vendittis E. Rossi B. Conte M.. (2010). Structure and flexibility in cold-adapted iron superoxide dismutases: the case of the enzyme isolated from *Pseudoalteromonas haloplanktis*. J. Struct. Biol. 172, 343–352. 10.1016/j.jsb.2010.08.008 20732427

[B188] Metpally R. P. R. Reddy B. V. B. (2009). Comparative proteome analysis of psychrophilic versus mesophilic bacterial species: insights into the molecular basis of cold adaptation of proteins. BMC Genomics 10:11. 10.1186/1471-2164-10-11 19133128 PMC2653534

[B189] Miao L. L. Hou Y. J. Fan H. X. Qu J. Qi C. Liu Y.. (2016). Molecular structural basis for the cold adaptedness of the psychrophilic beta-glucosidase BglU in *Micrococcus antarcticus*. Appl. Environ. Microbiol. 82, 2021–2030. 10.1128/AEM.03158-15 26801571 PMC4807509

[B190] Michaux C. Massant J. Kerff F. Frère J. Docquier J. Vandenberghe I.. (2008). Crystal structure of a cold−adapted class C β−lactamase. FEBS J. 275, 1687–1697. 10.1111/j.1742-4658.2008.06324.x 18312599

[B191] Miteva V. (2008). Bacteria in snow and glacier ice, in Psychrophiles: From Biodiversity to Biotechnology, eds Margesin R. Schinner F. Marx J.-C. Gerday C.(Berlin; Heilderberg: Springer), 31–50.

[B192] Mitsuya D. Tanaka S. Matsumura H. Urano N. Takano K. Ogasahara K.. (2014). Strategy for cold adaptation of the tryptophan synthase alpha subunit from the psychrophile *Shewanella frigidimarina* K14-2: crystal structure and physicochemical properties. J. Biochem. 155, 73–82. 10.1093/jb/mvt098 24163283

[B193] Mohammed S. Te'o J. Nevalainen H. (2013). A gene encoding a new cold-active lipase from an Antarctic isolate of *Penicillium expansum*. Curr. Genet. 59, 129–137. 10.1007/s00294-013-0394-x 23779196

[B194] Mohr P. W. Krawiec S. (1980). Temperature characteristics and Arrhenius plots for nominal psychrophiles, mesophiles and thermophiles. J. Gen. Microbiol. 121, 311–317. 10.1099/00221287-121-2-311 7264599

[B195] Monroe J. D. Storm A. R. Badley E. M. Lehman M. D. Platt S. M. Saunders L. K.. (2014). Beta-Amylase1 and beta-amylase3 are plastidic starch hydrolases in Arabidopsis that seem to be adapted for different thermal, pH, and stress conditions. Plant Physiol. 166, 1748–1763. 10.1104/pp.114.246421 25293962 PMC4256876

[B196] Morita R. Y. (1975). Psychrophilic bacteria. Bacteriol. Rev. 39, 144–167. 1095004 10.1128/br.39.2.144-167.1975 PMC413900

[B197] Morita Y. Nakamura T. Hasan Q. Murakami Y. Yokoyama K. Tamiya E. (1997). Cold-active enzymes from cold-adapted bacteria. J. Am. Oil Chem. Soc. 74, 441–444. 10.1007/s11746-997-0103-3

[B198] Nakagawa T. Ikehata R. Myoda T. Miyaji T. Tomizuka N. (2007). Overexpression and functional analysis of cold-active β-galactosidase from *Arthrobacter psychrolactophilus* strain F2. Protein Expr. Purif. 54, 295–299. 10.1016/j.pep.2007.03.010 17459724

[B199] Neang P. M. Subileau M. Perrier V. Dubreucq E. (2014). Homologous yeast lipases/acyltransferases exhibit remarkable cold-active properties. Appl. Microbiol. Biotechnol. 98, 8927–8936. 10.1007/s00253-014-5776-6 24770385

[B200] Nel A. J. Tuffin I. M. Sewell B. T. Cowan D. A. (2011). Unique aliphatic amidase from a psychrotrophic and haloalkaliphilic nesterenkonia isolate. Appl. Environ. Microbiol. 77, 3696–3702. 10.1128/AEM.02726-10 21498772 PMC3127607

[B201] Nevalainen H. Bradner R. Wadud S. Mohammed S. McRae C. Te'o J. (2012). Enzyme activities and biotechnological applications of cold-active microfungi, in Extremophiles: Microbiology and Biotechnology, ed Anitori R. P.(Norfolk, UK:Horizon Scientific Press), 89–108.

[B202] Niiranen L. Espelid S. Karlsen C. R. Mustonen M. Paulsen S. M. Heikinheimo P.. (2007). Comparative expression study to increase the solubility of cold adapted Vibrio proteins in *Escherichia coli*. Protein Expr. Purif. 52, 210–218. 10.1016/j.pep.2006.09.005 17064934

[B203] Nobeli I. Favia A. D. Thornton J. M. (2009). Protein promiscuity and its implications for biotechnology. Nat. Biotechnol. 27, 157–167. 10.1038/nbt1519 19204698

[B204] Novototskaya-Vlasova K. Petrovskaya L. Kryukova E. Rivkina E. Dolgikh D. Kirpichnikov M. (2013a). Expression and chaperone-assisted refolding of a new cold-active lipase from *Psychrobacter cryohalolentis* K5(T). Protein Expr. Purif. 91, 96–103. 10.1016/j.pep.2013.07.011 23891837

[B205] Novototskaya-Vlasova K. Petrovskaya L. E. Rivkina E. M. Dolgikh D. A. Kirpichnikov M. P. (2013b). Characterization of a cold-active lipase from *Psychrobacter cryohalolentis* K5(T) and its deletion mutants. Biochemistry (Mosc) 78, 385–394. 10.1134/S000629791304007X 23590441

[B206] Novototskaya-Vlasova K. Petrovskaya L. Yakimov S. Gilichinsky D. (2012). Cloning, purification, and characterization of a cold-adapted esterase produced by *Psychrobacter cryohalolentis* K5T from Siberian cryopeg. FEMS Microbiol. Ecol. 82, 367–375. 10.1111/j.1574-6941.2012.01385.x 22486752

[B207] Olivera-Nappa A. Reyes F. Andrews B. A. Asenjo J. A. (2013). Cold adaptation, Ca^2+^ dependency and autolytic stability are related features in a highly active cold-adapted trypsin resistant to autoproteolysis engineered for biotechnological applications. PLoS ONE 8:e72355. 10.1371/journal.pone.0072355 23951314 PMC3741176

[B208] Olufsen M. Smalås A. O. Moe E. Brandsdal B. O. (2005). Increased flexibility as a strategy for cold adaptation: a comparative molecular dynamics study of cold- and warm-active uracil DNA glycosylase. J. Biol. Chem. 280, 18042–18048. 10.1074/jbc.M500948200 15749696

[B209] Pan X. Tu T. Wang L. Luo H. Ma R. Shi P.. (2014). A novel low-temperature-active pectin methylesterase from *Penicillium chrysogenum* F46 with high efficiency in fruit firming. Food Chem. 162, 229–234. 10.1016/j.foodchem.2014.04.069 24874380

[B210] Papaleo E. Olufsen M. De Gioia L. Brandsdal B. O. (2007). Optimization of electrostatics as a strategy for cold-adaptation: a case study of cold-and warm-active elastases. J. Mol. Graph. Model. 26, 93–103. 10.1016/j.jmgm.2006.09.012 17084098

[B211] Papaleo E. Riccardi L. Villa C. Fantucci P. De Gioia L. (2006). Flexibility and enzymatic cold-adaptation: a comparative molecular dynamics investigation of the elastase family. Biochim. Biophys. Acta 1764, 1397–1406. 10.1016/j.bbapap.2006.06.005 16920043

[B212] Park D. J. Lee Y. S. Choi Y. L. (2013). Characterization of a cold-active beta-glucosidase from *Paenibacillus xylanilyticus* KJ-03 capable of hydrolyzing isoflavones daidzin and genistin. Protein J. 32, 579–584. 10.1007/s10930-013-9520-3 24141566

[B213] Parra L. P. Agudo R. Reetz M. T. (2013). Directed evolution by using iterative saturation mutagenesis based on multiresidue sites. Chembiochem 14, 2301–2309. 10.1002/cbic.201300486 24136881

[B214] Parra L. P. Espina G. Devia J. Salazar O. Andrews B. Asenjo J. A. (2015). Identification of lipase encoding genes from Antarctic seawater bacteria using degenerate primers: expression of a cold-active lipase with high specific activity. Enzyme Microb. Technol. 68, 56–61. 10.1016/j.enzmictec.2014.10.004 25435506

[B215] Parra L. P. Reyes F. Acevedo J. P. Salazar O. Andrews B. A. Asenjo J. A. (2008). Cloning and fusion expression of a cold-active lipase from marine Antarctic origin. Enzyme Microb. Technol. 42, 371–377. 10.1016/j.enzmictec.2007.11.003

[B216] Parrilli E. De Vizio D. Cirulli C. Tutino M. L. (2008a). Development of an improved *Pseudoalteromonas haloplanktis* TAC125 strain for recombinant protein secretion at low temperature. Microb. Cell Fact. 7, 1. 10.1186/1475-2859-7-2 18257924 PMC2275215

[B217] Parrilli E. Duilio A. Tutino M. L. (2008b). Heterologous protein expression in psychrophilic hosts, in Psychrophiles: From Biodiversity to Biotechnology, eds Margesin R. Schinner F. Marx J.-C. Gerday C. (Berlin; Heidelberg: Springer), 365–379.

[B218] Pawlak-Szukalska A. Wanarska M. Popinigis A. T. Kur J. (2014). A novel cold-active β-galactosidase with transglycosylation activity from the Antarctic *Arthrobacter sp*. 20B “ Gene cloning, purification and characterization. Process Biochem. 49, 2122–2133. 10.1016/j.procbio.2014.09.018

[B219] Pedersen H. L. Willassen N. P. Leiros I. (2009). The first structure of a cold-adapted superoxide dismutase (SOD): biochemical and structural characterization of iron SOD from *Aliivibrio salmonicida. Acta Crystallogr. Sect*. F Struct. Biol. Cryst. Commun. 65, 84–92. 10.1107/s1744309109001110 PMC2635881 19193992

[B220] Petrovskaya L. E. Novototskaya-Vlasova K. Kryukova E. A. Rivkina E. M. Dolgikh D. A. Kirpichnikov M. P. (2015). Cell surface display of cold-active esterase EstPc with the use of a new autotransporter from *Psychrobacter cryohalolentis* K5(T). Extremophiles 19, 161–170. 10.1007/s00792-014-0695-0 25253411

[B221] Pikuta E. V. Hoover R. B. Tang J. (2007). Microbial extremophiles at the limits of life. Crit. Rev. Microbiol. 33, 183–209. 10.1080/10408410701451948 17653987

[B222] Qin Y. Huang Z. Liu Z. (2014). A novel cold-active and salt-tolerant alpha-amylase from marine bacterium *Zunongwangia profunda*: molecular cloning, heterologous expression and biochemical characterization. Extremophiles 18, 271–281. 10.1007/s00792-013-0614-9 24318109

[B223] Qing G. Ma L. Khorchid A. Swapna G. Mal T. K. Takayama M. M.. (2004). Cold-shock induced high-yield protein production in *Escherichia coli*. Nat. Biotechnol. 22, 877–882. 10.1038/nbt984 15195104

[B224] Qoura F. Kassab E. Reisse S. Antranikian G. Brueck T. (2015). Characterization of a new, recombinant thermo-active subtilisin-like serine protease derived from *Shewanella arctica*. J. Mol. Catal. B Enzym. 116, 16–23. 10.1016/j.molcatb.2015.02.015

[B225] Rahman M. A. Culsum U. Tang W. Zhang S. W. Wu G. Liu Z. (2016). Characterization of a novel cold active and salt tolerant esterase from *Zunongwangia profunda*. Enzyme Microb. Technol. 85, 1–11. 10.1016/j.enzmictec.2015.12.013 26920474

[B226] Rajaei S. Noghabi K. A. Sadeghizadeh M. Zahiri H. S. (2015). Characterization of a pH and detergent-tolerant, cold-adapted type I pullulanase from *Exiguobacterium* sp. SH3. Extremophiles 19, 1145–1155. 10.1007/s00792-015-0786-6 26349928

[B227] Ramírez-Sarmiento C. A. Baez M. Wilson C. A. Babul J. Komives E. A. Guixé V. (2013). Observation of solvent penetration during cold denaturation of *E. coli* phosphofructokinase-2. Biophys. J. 104, 2254–2263. 10.1016/j.bpj.2013.04.024 23708365 PMC3660636

[B228] Reetz M. T. (2013). The importance of additive and non-additive mutational effects in protein engineering. Angew. Chem. Int. Ed. Engl. 52, 2658–2666. 10.1002/anie.201207842 23382001

[B229] Rhimi M. Bajic G. Ilhammami R. Boudebbouze S. Maguin E. Haser R.. (2011). The acid-tolerant L-arabinose isomerase from the mesophilic *Shewanella sp*. ANA-3 is highly active at low temperatures. Microb. Cell Fact. 10, 1–11. 10.1186/1475-2859-10-96 22074172 PMC3248863

[B230] Riise E. K. Lorentzen M. S. Helland R. Smalås A. Leiros H. Willassen N. P. (2007). The first structure of a cold-active catalase from *Vibrio salmonicida* at 1.96 Å reveals structural aspects of cold adaptation. Acta Crystallogr. D Biol. Crystallogr. 63, 135–148. 10.1107/S0907444906043812 17242507

[B231] Rivkina E. Laurinavichius K. McGrath J. Tiedje J. Shcherbakova V. Gilichinsky D. (2004). Microbial life in permafrost. Adv. Space Res. 33, 1215–1221. 10.1016/j.asr.2003.06.024 15806703

[B232] Rivkina E. M. Friedmann E. I. McKay C. P. Gilichinsky D. A. (2000). Metabolic activity of permafrost bacteria below the freezing point. Appl. Environ. Microbiol. 66, 3230–3233. 10.1128/AEM.66.8.3230-3233.2000 10919774 PMC92138

[B233] Rojas-Contreras J. de la Rosa A. P. De Leon-Rodriguez A. (2015). Expression and characterization of a recombinant psychrophilic Cu/Zn superoxide dismutase from *Deschampsia antarctica* E. Desv. [Poaceae]. Appl. Biochem. Biotechnol. 175, 3287–3296. 10.1007/s12010-015-1496-3 25638267

[B234] Russell N. Fukunaga N. (1990). A comparison of thermal adaptation of membrane lipids in psychrophilic and thermophilic bacteria. FEMS Microbiol. Lett. 75, 171–182. 10.1111/j.1574-6968.1990.tb04093.x

[B235] Russell N. J. (1998). Molecular adaptations in psychrophilic bacteria: potential for biotechnological applications. Adv. Biochem. Eng. Biotechnol. 61, 1–21. 10.1007/BFb0102287 9670796

[B236] Russell N. J. (2000). Toward a molecular understanding of cold activity of enzymes from psychrophiles. Extremophiles 4, 83–90. 10.1007/s007920050141 10805562

[B237] Russell R. J. Gerike U. Danson M. J. Hough D. W. Taylor G. L. (1998). Structural adaptations of the cold-active citrate synthase from an Antarctic bacterium. Structure 6, 351–361. 10.1016/S0969-2126(98)00037-9 9551556

[B238] Saha B. C. Demirjian D. C. (eds.). (2001). Advances in enzyme development and applied industrial biocatalysis, in *ACS Symposium Series* (Washington, DC: American Chemical Society), 2–12.

[B239] Sarmiento F. Peralta R. Blamey J. M. (2015). Cold and hot extremozymes: industrial relevance and current trends. Front. Bioeng. Biotechnol. 3:148. 10.3389/fbioe.2015.00148 26539430 PMC4611823

[B240] Saunders N. F. Thomas T. Curmi P. M. Mattick J. S. Kuczek E. Slade R.. (2003). Mechanisms of thermal adaptation revealed from the genomes of the Antarctic Archaea *Methanogenium frigidum* and Methanococcoides burtonii. Genome Res. 13, 1580–1588. 10.1101/gr.1180903 12805271 PMC403754

[B241] Schmidt M. Stougaard P. (2010). Identification, cloning and expression of a cold-active beta-galactosidase from a novel Arctic bacterium, *Alkalilactibacillus ikkense*. Environ. Technol. 31, 1107–1114. 10.1080/09593331003677872 20718293

[B242] Schrøder Leiros H. Willassen N. P. Smalås A. O. (2000). Structural comparison of psychrophilic and mesophilic trypsins. Eur. J. Biochem. 267, 1039–1049. 10.1046/j.1432-1327.2000.01098.x 10672012

[B243] Shakiba M. H. Ali M. S. Rahman R. N. Salleh A. B. Leow T. C. (2016). Cloning, expression and characterization of a novel coldadapted GDSL family esterase from *Photobacterium sp*. strain J15. Extremophiles 20, 44–55. 10.1007/s00792-015-0796-4 26475626

[B244] Shi Y. Wang Q. Hou Y. Hong Y. Han X. Yi J.. (2014). Molecular cloning, expression and enzymatic characterization of glutathione S-transferase from Antarctic sea-ice bacteria *Pseudoalteromonas sp*. ANT506. Microbiol. Res. 169, 179–184. 10.1016/j.micres.2013.06.012 23890723

[B245] Shuo-shuo C. Xue-zheng L. Ji-hong S. (2011). Effects of co-expression of molecular chaperones on heterologous soluble expression of the cold-active lipase Lip-948. Protein Expr. Purif. 77, 166–172. 10.1016/j.pep.2011.01.009 21272645

[B246] Siddiqui K. S. (2015). Some like it hot, some like it cold: temperature dependent biotechnological applications and improvements in extremophilic enzymes. Biotechnol. Adv. 33, 1912–1922. 10.1016/j.biotechadv.2015.11.001 26585268

[B247] Siddiqui K. S. Cavicchioli R. (2006). Cold-adapted enzymes. Annu. Rev. Biochem. 75, 403–433. 10.1146/annurev.biochem.75.103004.142723 16756497

[B248] Sigtryggsdóttir Á. R. Papaleo E. Thorbjarnardóttir S. H. Kristjánsson M. M. (2014). Flexibility of cold-and heat-adapted subtilisin-like serine proteinases evaluated with fluorescence quenching and molecular dynamics. Biochim. Biophys. Acta 1844, 705–712. 10.1016/j.bbapap.2014.02.009 24561657

[B249] Skalova T. Dohnálek J. Spiwok V. Lipovová P. Vondráèková E. Petroková H.. (2005). Cold-active β-galactosidase from *Arthrobacter* sp. C2-2 forms compact 660kDa hexamers: crystal structure at 1.9 Å resolution. J. Mol. Biol. 353, 282–294. 10.1016/j.jmb.2005.08.028 16171818

[B250] Smalås A. O. Heimstad E. S. Hordvik A. Willassen N. P. Male R. (1994). Cold adaption of enzymes: structural comparison between salmon and bovine trypsins. Proteins 20, 149–166. 10.1002/prot.340200205 7846025

[B251] Stemmer W. P. (1994). Rapid evolution of a protein *in vitro* by DNA shuffling. Nature 370, 389–391. 10.1038/370389a0 8047147

[B252] Struvay C. Feller G. (2012). Optimization to low temperature activity in psychrophilic enzymes. Int. J. Mol. Sci. 13, 11643–11665. 10.3390/ijms130911643 23109875 PMC3472767

[B253] Taguchi S. Ozaki A. Momose H. (1998). Engineering of a cold-adapted protease by sequential random mutagenesis and a screening system. Appl. Environ. Microbiol. 64, 492–495. 9464383 10.1128/aem.64.2.492-495.1998 PMC106071

[B254] Taguchi S. Ozaki A. Nonaka T. Mitsui Y. Momose H. (1999). A cold-adapted protease engineered by experimental evolution system. J. Biochem. 126, 689–693. 10.1093/oxfordjournals.jbchem.a022504 10502676

[B255] Tanaka D. Yoneda S. Yamashiro Y. Sakatoku A. Kayashima T. Yamakawa K.. (2012). Characterization of a new cold-adapted lipase from *Pseudomonas* sp. TK-3. Appl. Biochem. Biotechnol. 168, 327–338. 10.1007/s12010-012-9776-7 22870801

[B256] Tang M. A. K. Motoshima H. Watanabe K. (2012). Fluorescence studies on the stability, flexibility and substrate-induced conformational changes of acetate kinases from psychrophilic and mesophilic bacteria. Protein J. 31, 337–344. 10.1007/s10930-012-9408-7 22481532

[B257] Tang W. L. Zhao H. (2009). Industrial biotechnology: tools and applications. Biotechnol. J. 4, 1725–1739. 10.1002/biot.200900127 19844915

[B258] Tattersall G. J. Sinclair B. J. Withers P. C. Fields P. A. Seebacher F. Cooper C. E.. (2012). Coping with thermal challenges: physiological adaptations to environmental temperatures. Compr. Physiol. 3, 2151–2202. 10.1002/cphy.c110055 23723035

[B259] Temperton B. Giovannoni S. J. (2012). Metagenomics: microbial diversity through a scratched lens. Curr. Opin. Microbiol. 15, 605–612. 10.1016/j.mib.2012.07.001 22831844

[B260] Tiberti M. Papaleo E. (2011). Dynamic properties of extremophilic subtilisin-like serine-proteases. J. Struct. Biol. 174, 69–83. 10.1016/j.jsb.2011.01.006 21276854

[B261] Tindbaek N. Svendsen A. Oestergaard P. R. Draborg H. (2004). Engineering a substrate-specific cold-adapted subtilisin. Protein Eng. Des. Sel. 17, 149–156. 10.1093/protein/gzh019 15047911

[B262] Toyota E. Ng K. K. Kuninaga S. Sekizaki H. Itoh K. Tanizawa K.. (2002). Crystal structure and nucleotide sequence of an anionic trypsin from chum salmon (*Oncorhynchus keta*) in comparison with Atlantic salmon (*Salmo salar*) and bovine trypsin. J. Mol. Biol. 324, 391–397. 10.1016/S0022-2836(02)01097-5 12445776

[B263] Truongvan N. Jang S. Lee C. (2016). Flexibility and stability trade-off in active site of cold-adapted *Pseudomonas mandelii* Esterase EstK. Biochemistry 55, 3542–3549. 10.1021/acs.biochem.6b00177 27259687

[B264] Tsigos I. Mavromatis K. Tzanodaskalaki M. Pozidis C. Kokkinidis M. Bouriotis V. (2001). Engineering the properties of a cold active enzyme through rational redesign of the active site. Eur. J. Biochem. 268, 5074–5080. 10.1046/j.0014-2956.2001.02432.x 11589698

[B265] Tsuruta H. Mikami B. Aizono Y. (2005). Crystal structure of cold-active protein-tyrosine phosphatase from a psychrophile, *Shewanella sp.* J. Biochem. 137, 69–77. 10.1093/jb/mvi010 15713885

[B266] Tsuruta H. Mikami B. Higashi T. Aizono Y. (2010). Crystal structure of cold-active alkaline phosphatase from the psychrophile *Shewanella sp*. Biosci. Biotechnol. Biochem. 74, 69–74. 10.1271/bbb.90563 20057143

[B267] Tsuruta H. Mikami B. Yamamoto C. Yamagata H. (2008). The role of group bulkiness in the catalytic activity of psychrophile cold−active protein tyrosine phosphatase. FEBS J. 275, 4317–4328. 10.1111/j.1742-4658.2008.06575.x 18647345

[B268] Tu T. Meng K. Bai Y. Shi P. Luo H. Wang Y.. (2013). High-yield production of a low-temperature-active polygalacturonase for papaya juice clarification. Food Chem. 141, 2974–2981. 10.1016/j.foodchem.2013.05.132 23871048

[B269] Ueda M. Ito A. Nakazawa M. Miyatake K. Sakaguchi M. Inouye K. (2014). Cloning and expression of the cold-adapted endo-1,4-beta-glucanase gene from *Eisenia fetida*. Carbohydr. Polym. 101, 511–516. 10.1016/j.carbpol.2013.09.057 24299806

[B270] Van Petegem F. Collins T. Meuwis M. A. Gerday C. Feller G. Van Beeumen J. (2003). The structure of a cold-adapted family 8 xylanase at 1.3 A resolution. Structural adaptations to cold and investigation of the active site. J. Biol. Chem. 278, 7531–7539. 10.1074/jbc.M206862200 12475991

[B271] Vester J. K. Glaring M. A. Stougaard P. (2015). Improved cultivation and metagenomics as new tools for bioprospecting in cold environments. Extremophiles 19, 17–29. 10.1007/s00792-014-0704-3 25399309 PMC4272415

[B272] Vincent V. Aghajari N. Pollet N. Boisson A. Boudebbouze S. Haser R.. (2013). The acid tolerant and cold-active beta-galactosidase from *Lactococcus lactis* strain is an attractive biocatalyst for lactose hydrolysis. Antonie Van Leeuwenhoek 103, 701–712. 10.1007/s10482-012-9852-6 23180374

[B273] Violot S. Aghajari N. Czjzek M. Feller G. Sonan G. K. Gouet P.. (2005). Structure of a full length psychrophilic cellulase from *Pseudoalteromonas haloplanktis* revealed by X-ray diffraction and small angle X-ray scattering. J. Mol. Biol. 348, 1211–1224. 10.1016/j.jmb.2005.03.026 15854656

[B274] Wang E. Koutsioulis D. Leiros H. S. Andersen O. A. Bouriotis V. Hough E.. (2007). Crystal structure of alkaline phosphatase from the Antarctic bacterium TAB5. J. Mol. Biol. 366, 1318–1331. 10.1016/j.jmb.2006.11.079 17198711

[B275] Wang G. Luo H. Wang Y. Huang H. Shi P. Yang P.. (2011). A novel cold-active xylanase gene from the environmental DNA of goat rumen contents: direct cloning, expression and enzyme characterization. Bioresour. Technol. 102, 3330–3336. 10.1016/j.biortech.2010.11.004 21106368

[B276] Wang G. Wang Q. Lin X. Ng T. B. Yan R. Lin J.. (2016). A novel cold-adapted and highly salt-tolerant esterase from *Alkalibacterium* sp. SL3 from the sediment of a soda lake. Sci. Rep. 6:19494. 10.1038/srep19494 26915906 PMC4768246

[B277] Wang M. Si T. Zhao H. (2012). Biocatalyst development by directed evolution. Bioresour. Technol. 115, 117–125. 10.1016/j.biortech.2012.01.054 22310212 PMC3351540

[B278] Wang Q. Hou Y. Shi Y. Han X. Chen Q. Hu Z.. (2014). Cloning, expression, purification, and characterization of glutaredoxin from Antarctic sea-ice bacterium *Pseudoalteromonas* sp. AN178. Biomed Res. Int. 2014:246871. 10.1155/2014/246871 25110664 PMC4109671

[B279] Wang S. Yang Y. Yang R. Zhang J. Chen M. Matsukawa S.. (2014). Cloning and characterization of a cold-adapted endo-1, 5-α-l-arabinanase from *Paenibacillus polymyxa* and rational design for acidic applicability. J. Agric. Food Chem. 62, 8460–8469. 10.1021/jf501328n 25077565

[B280] Wang S. Y. Hu W. Lin X. Y. Wu Z. H. Li Y. Z. (2012). A novel cold-active xylanase from the cellulolytic myxobacterium *Sorangium cellulosum* So9733-1: gene cloning, expression, and enzymatic characterization. Appl. Microbiol. Biotechnol. 93, 1503–1512. 10.1007/s00253-011-3480-3 21792591

[B281] Watanabe S. Yasutake Y. Tanaka I. Takada Y. (2005). Elucidation of stability determinants of cold-adapted monomeric isocitrate dehydrogenase from a psychrophilic bacterium, *Colwellia maris*, by construction of chimeric enzymes. Microbiology 151, 1083–1094. 10.1099/mic.0.27667-0 15817777

[B282] Wei W. Ma J. Chen S. Q. Cai X. H. Wei D. Z. (2015). A novel cold-adapted type I pullulanase of *Paenibacillus polymyxa* Nws-pp2: *in vivo* functional expression and biochemical characterization of glucans hydrolyzates analysis. BMC Biotechnol. 15:96. 10.1186/s12896-015-0215-z 26481143 PMC4615870

[B283] Whitley D. Goldberg S. P. Jordan W. D. (1999). Heat shock proteins: a review of the molecular chaperones. J. Vasc. Surg. 29, 748–751. 10.1016/S0741-5214(99)70329-0 10194511

[B284] Wicka M. Wanarska M. Krajewska E. Pawlak-Szukalska A. Kur J. Cieslinski H. (2016). Cloning, expression, and biochemical characterization of a cold-active GDSL-esterase of a *Pseudomonas* sp. S9 isolated from Spitsbergen island soil. Acta Biochim. Pol. 63:1074. 10.18388/abp.2015_1074 26824293

[B285] Widderich N. Kobus S. Höppner A. Riclea R. Seubert A. Dickschat J. S.. (2016). Biochemistry and crystal structure of ectoine synthase: a metal-containing member of the cupin superfamily. PLoS ONE 11:e0151285. 10.1371/journal.pone.0151285 26986827 PMC4795551

[B286] Wierzbicka-Wos A. Cieslinski H. Wanarska M. Kozowska-Tylingo K. Hildebrandt P. Kur J. (2011). A novel cold-active β-D-galactosidase from the *Paracoccus* sp. 32d - gene cloning, purification and characterization. Microb. Cell Fact. 10, 1–12. 10.1186/1475-2859-10-108 22166118 PMC3268748

[B287] Wijma H. J. Floor R. J. Janssen D. B. (2013). Structure- and sequence-analysis inspired engineering of proteins for enhanced thermostability. Curr. Opin. Struct. Biol. 23, 588–594. 10.1016/j.sbi.2013.04.008 23683520

[B288] Wohlgemuth R. (2010). Biocatalysis - key to sustainable industrial chemistry. Curr. Opin. Biotechnol. 21, 713–724. 10.1016/j.copbio.2010.09.016 21030244

[B289] Wu G. Wu G. Zhan T. Shao Z. Liu Z. (2013a). Characterization of a cold-adapted and salt-tolerant esterase from a psychrotrophic bacterium *Psychrobacter pacificensis*. Extremophiles 17, 809–819. 10.1007/s00792-013-0562-4 23868329

[B290] Wu G. Zhang S. Zhang H. Zhang S. Liu Z. (2013b). A novel esterase from a psychrotrophic bacterium *Psychrobacter celer* 3Pb1 showed cold-adaptation and salt-tolerance. J. Mol. Catal. B Enzym. 98, 119–126. 10.1016/j.molcatb.2013.10.012

[B291] Wu G. Zhang X. Wei L. Wu G. Kumar A. Mao T.. (2015). A cold-adapted, solvent and salt tolerant esterase from marine *bacterium Psychrobacter pacificensis*. Int. J. Biol. Macromol. 81, 180–187. 10.1016/j.ijbiomac.2015.07.045 26231332

[B292] Wu S. Liu Y. Yan Q. Jiang Z. (2014). Gene cloning, functional expression and characterisation of a novel glycogen branching enzyme from *Rhizomucor miehei* and its application in wheat breadmaking. Food Chem. 159, 85–94. 10.1016/j.foodchem.2014.02.161 24767030

[B293] Xu H. Lan D. Yang B. Wang Y. (2015). Biochemical properties and structure analysis of a DAG-Like lipase from *Malassezia globosa*. Int. J. Mol. Sci. 16, 4865–4879. 10.3390/ijms16034865 25749469 PMC4394454

[B294] Yan Q. Duan X. Liu Y. Jiang Z. Yang S. (2016). Expression and characterization of a novel 1,3-regioselective cold-adapted lipase from *Rhizomucor endophyticus* suitable for biodiesel synthesis. Biotechnol. Biofuels 9, 86. 10.1186/s13068-016-0501-6 27081399 PMC4831154

[B295] Yang G. Ding Y. (2014). Recent advances in biocatalyst discovery, development and applications. Bioorg. Med. Chem. 22, 5604–5612. 10.1016/j.bmc.2014.06.033 25042559

[B296] Yang J. Dang H. (2011). Cloning and characterization of a novel cold-active endoglucanase establishing a new subfamily of glycosyl hydrolase family 5 from a psychrophilic deep-sea bacterium. FEMS Microbiol. Lett. 325, 71–76. 10.1111/j.1574-6968.2011.02413.x 22092864

[B297] Yoneda K. Sakuraba H. Muraoka I. Oikawa T. Ohshima T. (2010). Crystal structure of UDP−galactose 4−epimerase−like l−threonine dehydrogenase belonging to the intermediate short−chain dehydrogenase−reductase superfamily. FEBS J. 277, 5124–5132. 10.1111/j.1742-4658.2010.07916.x 21078123

[B298] Yoneta M. Sahara T. Nitta K. Takada Y. (2004). Characterization of chimeric isocitrate dehydrogenases of a mesophilic nitrogen-fixing bacterium, *Azotobacter vinelandii*, and a psychrophilic bacterium, Colwellia maris. Curr. Microbiol. 48, 383–388. 10.1007/s00284-003-4203-5 15060737

[B299] Yu Z. Tang B. Zhao D. Pang X. Qin Q. Zhou B.. (2015). Development of a cold-adapted pseudoalteromonas expression system for the pseudoalteromonas proteins intractable for the *Escherichia coli* system. PLoS ONE 10:e0137384. 10.1371/journal.pone.0137384 26333173 PMC4557933

[B300] Yumoto I. (2013). Cold-Adapted Microorganisms. Norfolk, UK: Horizon Scientific Press.

[B301] Zanphorlin L. M. de Giuseppe P. O. Honorato R. V. Tonoli C. C. Fattori J. Crespim E.. (2016). Oligomerization as a strategy for cold adaptation: structure and dynamics of the GH1 beta-glucosidase from *Exiguobacterium antarcticum* B7. Sci. Rep. 6, 23776. 10.1038/srep23776 27029646 PMC4815018

[B302] Závodszky P. Kardos J. Svingor Petsko, G. A. (1998). Adjustment of conformational flexibility is a key event in the thermal adaptation of proteins. Proc. Natl. Acad. Sci. U.S.A. 95, 7406–7411. 10.1073/pnas.95.13.7406 9636162 PMC22632

[B303] Zhang K. Guo Y. Yao P. Lin Y. Kumar A. Liu Z.. (2016). Characterization and directed evolution of BliGO, a novel glycine oxidase from *Bacillus licheniformis*. Enzyme Microb. Technol. 85, 12–18. 10.1016/j.enzmictec.2015.12.012 26920475

[B304] Zhang N. Suen W. C. Windsor W. Xiao L. Madison V. Zaks A. (2003). Improving tolerance of *Candida antarctica* lipase B towards irreversible thermal inactivation through directed evolution. Protein Eng. 16, 599–605. 10.1093/protein/gzg074 12968077

[B305] Zhang S. Sun M. Li T. Wang Q. Hao J. Han Y.. (2011). Structure analysis of a new psychrophilic marine protease. PLoS ONE 6:e26939. 10.1371/journal.pone.0026939 22132082 PMC3223159

[B306] Zhao D. Yu Z. Li P. Wu Z. Chen X. Shi M. (2011). Characterization of a cryptic plasmid pSM429 and its application for heterologous expression in psychrophilic Pseudoalteromonas. *Microb*. Cell Fact. 10, 1 10.1186/1475-2859-10-30 PMC3112385 21542941

[B307] Zhao W. Peng R. Xiong A. Fu X. Tian Y. Yao Q. (2012). Expression and characterization of a cold-active and xylose-stimulated beta-glucosidase from *Marinomonas* MWYL1 in *Escherichia coli*. Mol. Biol. Rep. 39, 2937–2943. 10.1007/s11033-011-1055-0 21681424

[B308] Zhao W. Zheng J. Zhou H. B. (2011). A thermotolerant and cold-active mannan endo-1,4-beta-mannosidase from *Aspergillus niger* CBS 513.88: constitutive overexpression and high-density fermentation in *Pichia pastoris*. Bioresour. Technol. 102, 7538–7547. 10.1016/j.biortech.2011.04.070 21632240

[B309] Zhao Y. Wakamatsu T. Doi K. Sakuraba H. Ohshima T. (2012). A psychrophilic leucine dehydrogenase from *Sporosarcina psychrophila*: purification, characterization, gene sequencing and crystal structure analysis. J. Mol. Catal. B Enzym. 83, 65–72. 10.1016/j.molcatb.2012.06.018

[B310] Zheng H. Liu Y. Sun M. Han Y. Wang J. Sun J.. (2014). Improvement of alkali stability and thermostability of *Paenibacillus campinasensis* Family-11 xylanase by directed evolution and site-directed mutagenesis. J. Ind. Microbiol. Biotechnol. 41, 153–162. 10.1007/s10295-013-1363-6 24212471

[B311] Zheng X. Chu X. Zhang W. Wu N. Fan Y. (2011). A novel cold-adapted lipase from *Acinetobacter sp.* XMZ-26: gene cloning and characterisation. Appl. Microbiol. Biotechnol. 90, 971–980. 10.1007/s00253-011-3154-1 21336927

[B312] Zheng Y. Li Y. Liu W. Chen C. Ko T. He M.. (2016). Structural insight into potential cold adaptation mechanism through a psychrophilic glycoside hydrolase family 10 endo-β-1, 4-xylanase. J. Struct. Biol. 193, 206–211. 10.1016/j.jsb.2015.12.010 26719223

[B313] Zhong C. Song S. Fang N. Liang X. Zhu H. Tang X.. (2009). Improvement of low−temperature caseinolytic activity of a thermophilic subtilase by directed evolution and site−directed mutagenesis. Biotechnol. Bioeng. 104, 862–870. 10.1002/bit.22473 19609954

[B314] Zhou J. Lu Q. Peng M. Zhang R. Mo M. Tang X.. (2015). Cold-active and NaCl-tolerant exo-inulinase from a cold-adapted *Arthrobacter sp*. MN8 and its potential for use in the production of fructose at low temperatures. J. Biosci. Bioeng. 119, 267–274. 10.1016/j.jbiosc.2014.08.003 25266375

[B315] Zhou J. Zhang R. Gao Y. Li J. Tang X. Mu Y.. (2012). Novel low-temperature-active, salt-tolerant and proteases-resistant endo-1,4-beta-mannanase from a new *Sphingomonas* strain. J. Biosci. Bioeng. 113, 568–574. 10.1016/j.jbiosc.2011.12.011 22265897

[B316] Zhou J. Zhang R. Shi P. Huang H. Meng K. Yuan T.. (2011). A novel low-temperature-active beta-glucosidase from symbiotic *Serratia sp*. TN49 reveals four essential positions for substrate accommodation. Appl. Microbiol. Biotechnol. 92, 305–315. 10.1007/s00253-011-3323-2 21559826

